# Total synthesis of *ent*-pavettamine

**DOI:** 10.3762/bjoc.17.99

**Published:** 2021-06-10

**Authors:** Memory Zimuwandeyi, Manuel A Fernandes, Amanda L Rousseau, Moira L Bode

**Affiliations:** 1 Molecular Sciences Institute, School of Chemistry, University of the Witwatersrand, Private Bag 3, PO WITS, 2050, South Africa

**Keywords:** chiral sulfoxide, *ent*-pavettamine, pavettamine, polyamine

## Abstract

Pavettamine, a plant toxin first isolated from *Pavetta harborii* in 1995, was previously identified as a polyamine with *C*_2_ symmetry and a 1,3-*syn*-diol moiety on a C_10_ carbon backbone – one of very few substituted polyamines to be isolated from nature. Its absolute configuration was later established by our first reported total synthesis in 2010. Herein we report the first total synthesis of the enantiomer of pavettamine, *ent*-pavettamine. The symmetrical structure of the molecule allows for the synthesis of a common C_5_ fragment that can be divergently transformed into two synthons for later convergent coupling to furnish the target carbon framework. Based on the success of the protocol we employed for the synthesis of the naturally occurring pavettamine, (*S*)-malic acid was again the starting material of choice for the synthesis of the two individual C_5_ fragments, with strategic differences in terminal-group manipulation allowing for the synthesis of *ent*-pavettamine rather than pavettamine. Chain extension and stereoselective ketone reduction were achieved using the (*R*)-methyl *p*-tolyl sulfoxide chiral auxiliary to give the desired 1,3-*syn*-diol C_5_ unit. A protecting-group strategy was also developed for the orthogonal protection of the alcohol and amine functional groups as they were unveiled. The functionalized C_5_ fragments were coupled via reductive amination revealing the C_10_ carbon backbone. Deprotection of the alcohol and amine functional groups successfully provided *ent*-pavettamine as a TFA salt.

## Introduction

**Figure 1:**
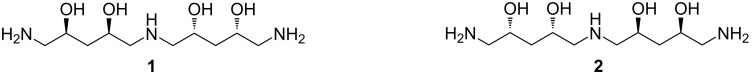
Structure of pavettamine **1** and its enantiomer **2**.

The identification and first reported synthesis of pavettamine (**1**) heralded the arrival of a novel and uniquely hydroxylated polyamine (PA) (Figure 1) [1]. In general, polyamines are described as aliphatic organic compounds with two or more primary amine substituents, connected by one or more unsubstituted methylene linkages, within their structure [2]. The interest in polyamines arises from the diverse roles and functions they play in biological systems [3–4]; for example, as stabilizers of RNA and DNA, secondary messengers, nutrients, antioxidants, growth factors, and metabolic regulators [5]. In addition to this, some PAs are currently being used as therapeutic drugs, being incorporated as drug conjugates, or are under investigation for other applications [6–12].

The elucidation of the structure of pavettamine revealed a new class of polyamines with a substituted methylene linkage. Biological studies have shown that this toxin is responsible for “quick disease” (gousiekte) in ruminant animals, which causes inhibition of protein synthesis in the cardiovascular organs [13]. The unique structure coupled with the biological effects of this polyamine prompted the current study, which aimed to establish a method for the synthesis of *ent*-pavettamine (**2**) so as to contribute towards a comprehensive structure–activity relationship study of pavettamine. With the absolute stereochemistry of pavettamine having been established previously [1], this study focused on developing a route for the synthesis of enantiomer **2** by modification of the established protocol used for the synthesis of **1**. The plan was that the alternative strategy would lead to an improvement in the overall yield whilst eliminating some of the troublesome steps.

## Results and Discussion

The synthetic protocol we originally used for **1** was adapted for the synthesis of **2** owing to the excellent stereochemical control achieved using this route [1]. A modular approach was employed as it was envisioned that using this protocol would allow for the synthesis of the desired compound in a reasonable number of steps. As before, (*S*)*-*malic acid (**3**) was chosen as the starting material because its inherent chirality reduced the complexity of the synthetic process. Given the fact that the target molecule has *C*_2_ symmetry, it was prudent that a synthetic route starting from a common C_5_ subunit be chosen, with subsequent functionalization giving rise to two C_5_ units with compatible groups required for linking: in this case an amine and an aldehyde. In order for the synthesis to yield *ent*-pavettamine instead of pavettamine, the key was to functionalize the “opposite end” of each of the C_5_ units required for linking, when compared to our original pavettamine synthesis [1]. This opposite functionalization ensured that the enantiomer would result from the synthesis.

**Scheme 1:**
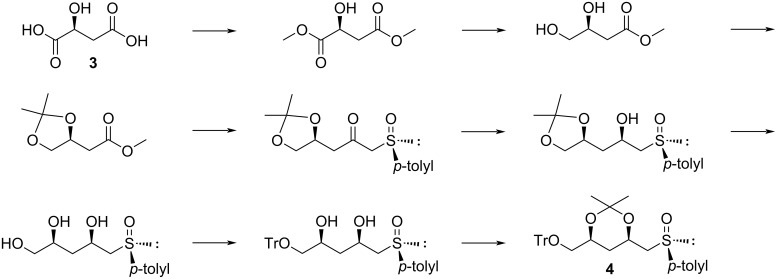
Established route for the synthesis of intermediate **4** [1].

For purposes of comparison between the original route and the route described here, our efforts commenced with the synthesis of the common C_5_ unit **4**, using our previously published methodology in an overall yield of 8% over 8 steps (Scheme 1) [1].

**Scheme 2:**
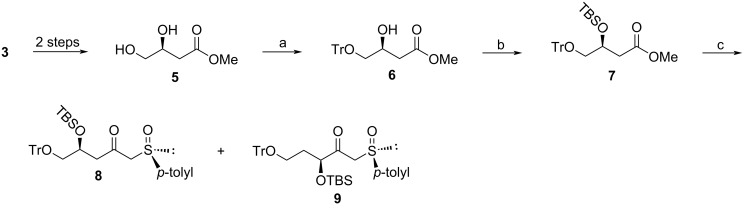
Alternative route. Reaction conditions: a) TrCl, pyridine, rt, overnight, 100%; b) DMAP, imidazole, TBS, DCM, rt, 12 h, 65%; c) LDA, (*R*)-methyl *p*-tolyl sulfoxide, THF, −78 °C to rt, 2.5 h, 55% yield of **8**.

An alternative strategy for the synthesis of **4** was developed so as to avoid a cumbersome continuous extraction step of a triol associated with the established method, in addition to improving the overall yield. The first step involved esterification of malic acid using methanol, followed by selective reduction of the resulting dimethyl ester using borane dimethyl sulfide (BMS) yielding diol **5** (Scheme 2) [1,14–15].

The step achieved chemo-differentiation of the carboxyl groups present in **3** whilst retaining the stereogenic center. Divergence from the original route to pavettamine occurred after recovery of diol **5**; and a different protecting group strategy was applied to arrive at synthon **4**. The primary alcohol of the diol **5** was regioselectively protected using the trityl group under basic conditions yielding **6** in quantitative yield. Our choice of the trityl protecting group was based on the fact that it could be selectively added in the presence of the secondary alcohol and selectively removed at a later stage in the presence of an orthogonally-protected secondary alcohol [16]. At this stage, the selective functionalization and protection of the primary and secondary hydroxy groups (and later amino groups) was critical for the successful synthesis of the target compound. Unfortunately, each protecting group incorporated increased the synthesis by two non-productive steps; nonetheless our aim to achieve an improved yield of intermediate **4** was met.

Subsequent TBS protection of the secondary alcohol **6** gave rise to compound **7** in a moderate yield of 65%. Once the ester precursor **7** had been successfully synthesized, the next step involved conversion of the ester into its β-keto sulfoxide via substitution with the anion of (*R*)-methyl *p*-tolyl sulfoxide according to published procedures [1,17]. The desired product **8** was recovered in a yield of 55% and a significant amount of side product **9** was recovered in a yield of 24%. The identity of the side product was not apparent at first because both products were light yellow solids with the same melting point of 115–116 °C and both had the same measured mass when analyzed by HRMS. The differences were that the specific optical rotation was measured to be +67.0° for compound **8** and +91.4° for compound **9** and their ^1^H NMR spectra differed for the signals close to the OTBS center.

**Figure 2:**
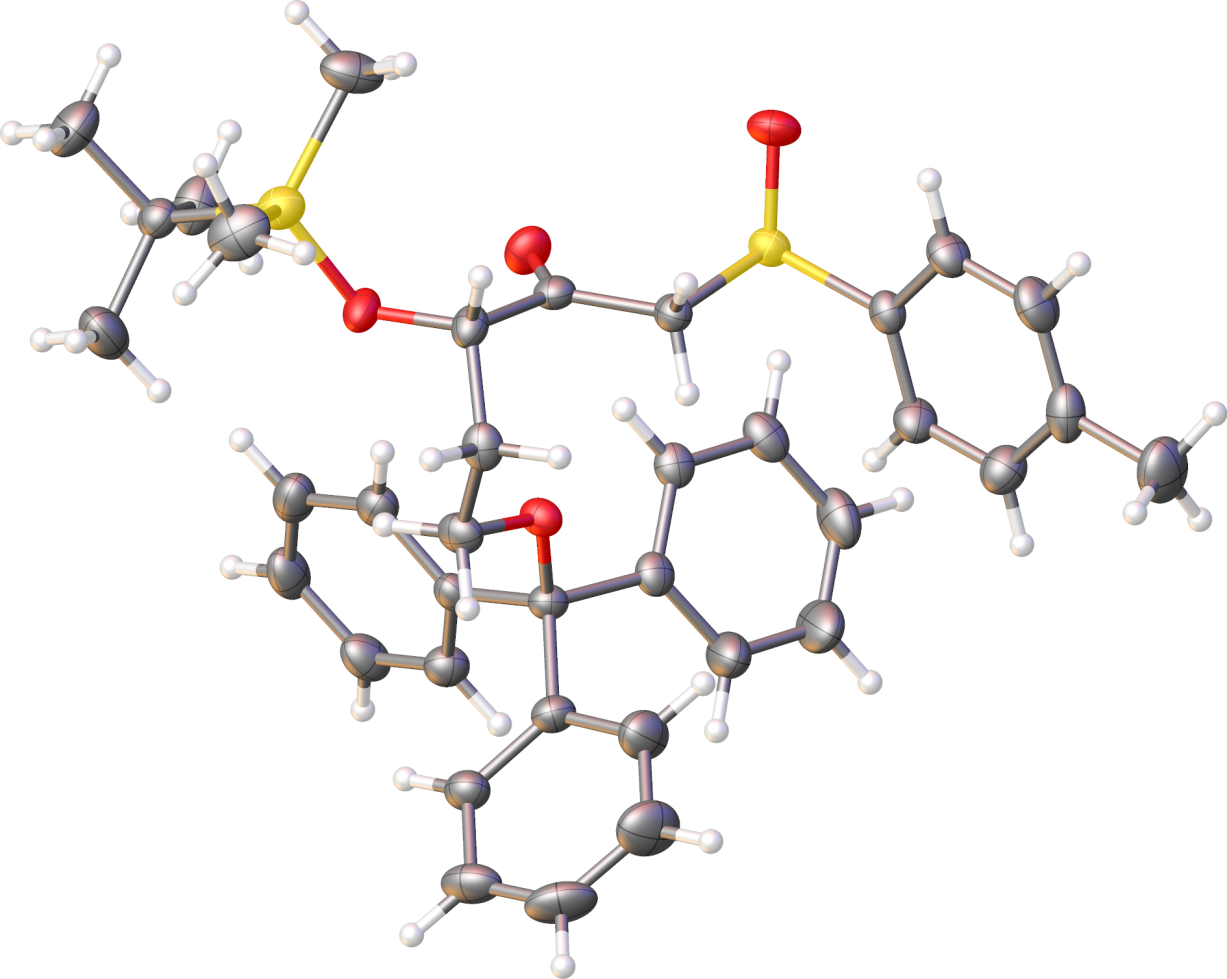
Crystal structure of compound **9**.

At this point, it was crucial to conclusively characterize the unexpected product **9**. Fortuitously, we were able to obtain crystals for this compound after several recrystallization attempts. The crystal structure for **9** (Figure 2) very surprisingly showed the OTBS group on the carbon atom α to the carbonyl group, instead of β to the carbonyl carbon for the desired compound **8**. These results were puzzling, and how this product could have been formed from reaction of compound **7** with (*R*)-methyl *p*-tolyl sulfoxide was not obvious.

**Scheme 3:**
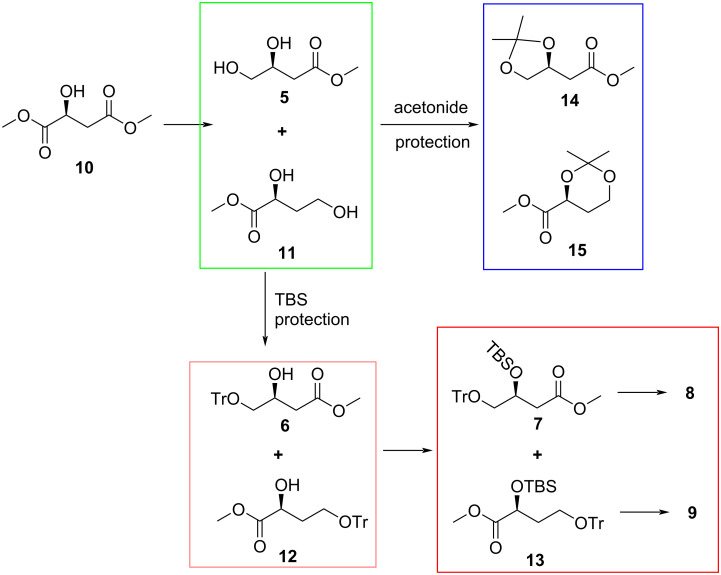
Sequence showing the source of compound **9**.

After investigating several possibilities coupled with careful examination of the preceding steps it became clear that compound **9** was not formed in the reaction of **7** with the sulfoxide auxiliary. Unbeknownst to us at the time, the formation of diol **5** was not as highly selective as expected and this reaction produced two inseparable diols, the desired product **5** and unwanted side product **11** (Scheme 3) which were not distinguishable by ^1^H NMR spectroscopy. Carrying side product **11** through the subsequent synthetic steps resulted in compounds **12** and **13** and ultimately product **9** (Scheme 2), the first point in the sequence where the isomeric products were separable and clearly distinguishable by ^1^H NMR spectroscopy.

The formation of **11** was not apparent when using an acetonide protection strategy (Scheme 1) because the desired product **14** was easily separated by column chromatography from the undesired compound **15**, emanating from diol **11** (Scheme 3). However, compound **12** was not separable from **6** by column chromatography and TLC visualization only showed a single spot. The presence of the unwanted side product **12** only became evident retrospectively when a close examination of the ^1^H NMR spectrum revealed a small extra methoxy signal, showing a ratio of between 21:1 and 9:1 of the desired product **6** to the unwanted material **12**. The product ratios changed with each subsequent synthetic step, which suggests different reaction rates occurred for each of the two isomers. Ultimately, a ratio of 55:24 was observed for compounds **8** and **9**. We attempted to eliminate or minimize the formation of diol **11** by performing the reduction step at temperatures lower than 15 °C but this significantly reduced the yield of the desired product, even with longer reaction times. Therefore, we had to be satisfied with separation of desired product **8** from unwanted **9** after the addition of the chiral auxiliary.

**Scheme 4:**
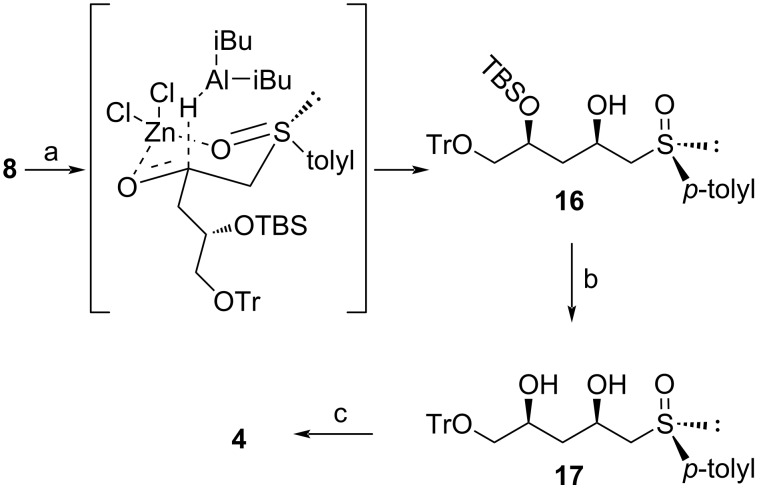
Stereoselective reduction of intermediate **8** as key step towards intermediate **4**. Reaction conditions: a) i) ZnCl_2_, rt, 2 h; ii) DIBALH, THF, −78 °C to rt, 2 h, 87%; b) TBAF, THF, 0 °C, 4 h, 87%; c) 5% *p*-TsOH, 2,2-dimethoxypropane, acetone, rt, 1 h, 86%.

Our attention then turned to the stereoselective reduction of **8**. Chelation-controlled reduction of **8** using ZnCl_2_ and DIBALH allowed for the successful formation of **16** as a single diastereomer (Scheme 4), as evidenced by ^1^H NMR spectroscopy [18].

**Figure 3:**
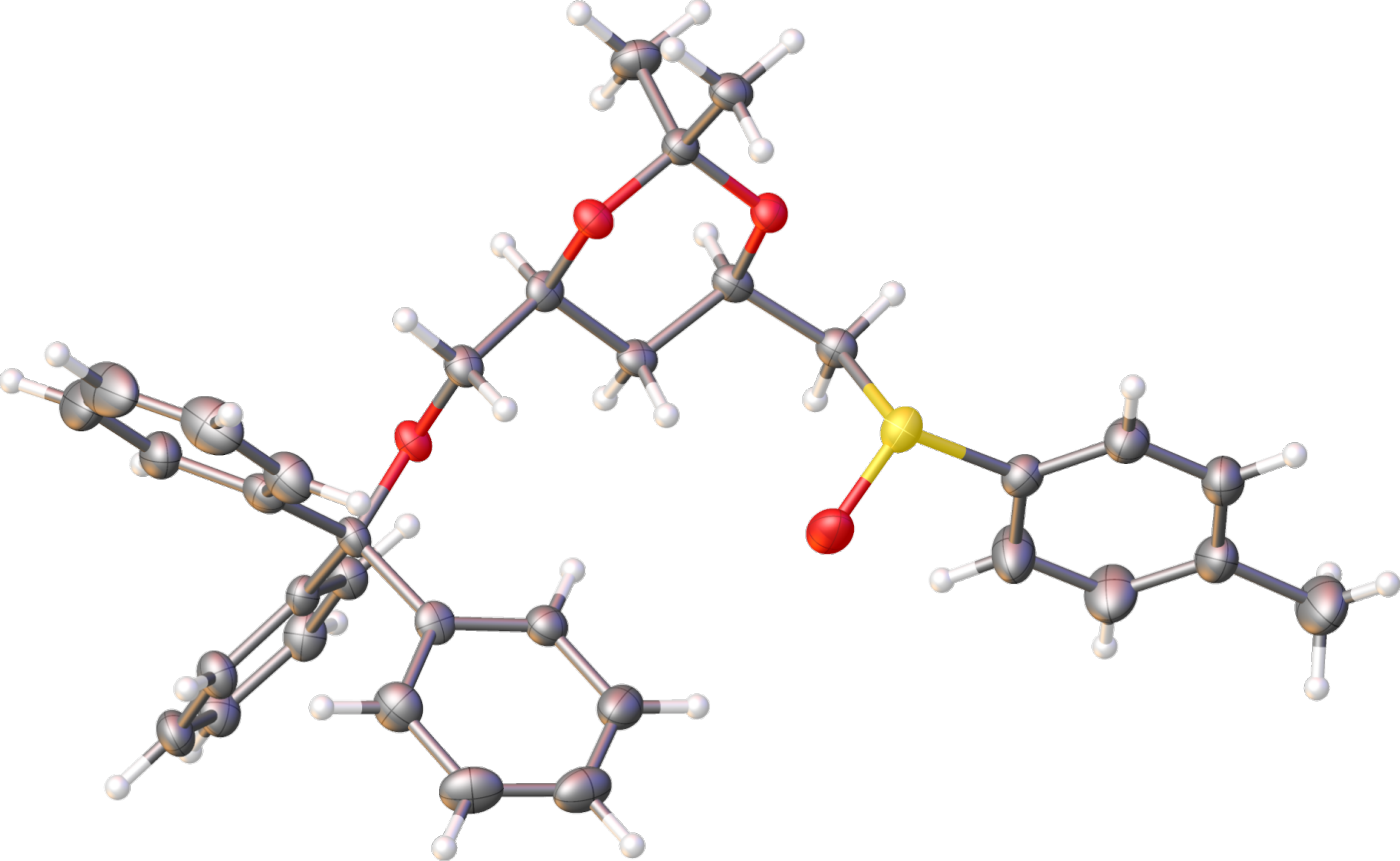
Single crystal X-ray structure of compound **4**.

TBS deprotection furnished **17**, followed by acetonide protection of the 1,3-diol functionality to yield the previously synthesized intermediate **4**. The acetonide protecting group was chosen because of its stability under the future reaction conditions in addition to it being a convenient monitor of the relative configuration of the 1,3-diol motif [19]. The group is also associated with ease of deprotection, which is well documented in carbohydrate and sugar chemistry [20]. Comparison of our new route involving trityl and TBS protections with our previously published acetonide protection route for the synthesis of **4**, showed that the new route doubled the overall yield to 16% for a similar number of steps. The specific optical rotation for **4** of +28.1° in acetone was comparable to that obtained previously [1]. Single crystal X-ray analysis of compound **4** (Figure 3), further confirmed the identity and stereochemistry of the compound.

**Scheme 5:**
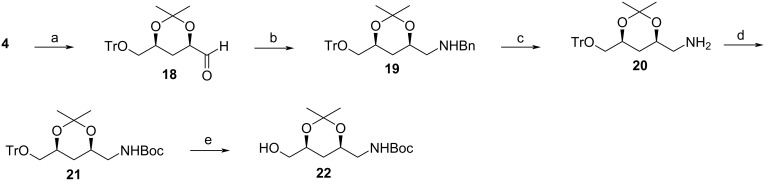
Synthesis of the C_5_ fragments from intermediate **4**. Reaction conditions: a) i) TFAA, collidine, 0 °C, 30 min; ii) K_2_CO_3_, rt, 30 min, 99%; b) benzylamine, DCE, THF, sodium triacetoxyborohydride, N_2_, rt, 24 h, 60%; c) ethanol, 10% Pd/C, H_2_, rt, 24 h, 84%; d) Boc anhydride, DMAP, THF, rt, overnight, 100%; e) Na, NH_3_, THF, -60 °C, 1 h, 81%.

Once the crucial 1,3-*syn*-diol moiety had been obtained, attention shifted to functionalization and differentiation of the terminal carbon atoms for the synthesis of the two C_5_ fragments. Intermediate **4** was subjected to the Pummerer rearrangement by treatment with TFAA in collidine at 0 °C, followed by hydrolysis with solid K_2_CO_3_ and water for 30 min (Scheme 5). The desired aldehyde **18** was recovered in an excellent yield of 99%, and product epimerization was not detected based on ^13^C NMR spectroscopic analysis. This was confirmed by analysis of the ^13^C chemical shifts of the acetonide group, whose values differ for the *syn*-1,3 diol when compared to the *anti*-1,3-diol as described after an extensive study by Rychnovsky et al*.* [21].

The reductive amination of the aldehyde with benzylamine allowed for the introduction of a protected terminal amine group to the C_5_ fragment, yielding **19** in a modest yield of 60%. The next step involved the selective removal of the trityl group so as to facilitate the synthesis of the two separate C_5_ fragments which would be coupled to yield the desired product. Unfortunately, the anticipated selective removal of the trityl group in the presence of an *N*-benzyl group did not yield the desired product [22–26]. Several selective trityl deprotection attempts gave different results: either the reaction did not proceed at all yielding starting material, or both the trityl and benzyl group were removed or all three protecting groups were removed. Based on these results, it was evident that the selective removal of the trityl group in the presence of the benzyl group is substrate specific. Eventually, hydrogenation under neutral conditions at atmospheric pressure for 24 h allowed for the selective removal of the benzyl group, affording amine **20** in a yield of 84%. The Boc group was chosen for the re-protection of the amine, furnishing **21** in quantitative yield. Due to the significant difference between the trityl and the Boc groups, selective trityl removal was now possible under Birch reduction conditions. The primary alcohol **22** was recovered in an excellent yield of 81%.

**Scheme 6:**
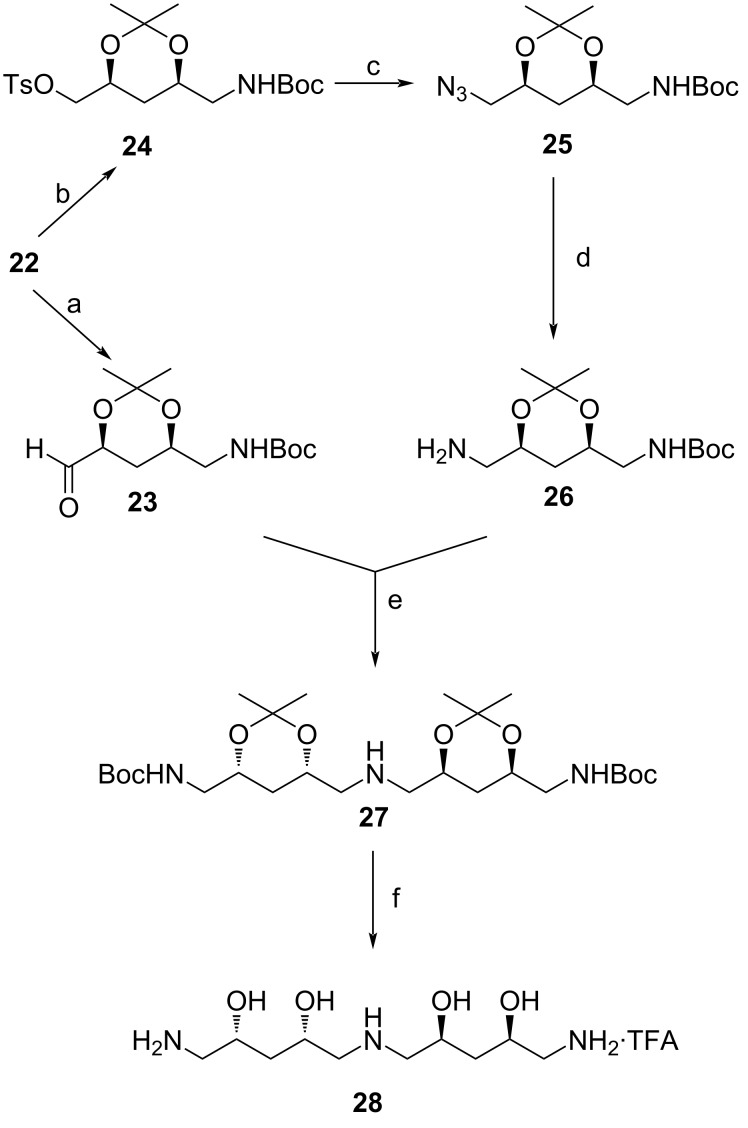
Synthesis of *ent*-pavettamine as the TFA salt **28**. Reaction conditions: a) IBX, DMSO, rt, overnight, quantitative yield; b) TsCl, DMAP, DCM, rt, 24 h, 83%; c) NaN_3_, DMF, 80 °C, 3 h, 75% yield; d) 10% Pd/C, H_2_, ethanol, rt, 12 h, quantitative yield; e) sodium triacetoxyborohydride, 1,2-DCE, THF, rt, 24 h, 95%; f) 95% TFA, 2.5% H_2_O, 2.5% triisopropylamine, rt, overnight, quantitative yield.

The alcohol was apportioned into two parts to synthesize the two required C_5_ fragments separately, by converting the alcohol to an amine for one fragment and an aldehyde for the second fragment. The conversion of the alcohol to the aldehyde was first attempted using MnO_2_ but only the starting material was recovered. Use of PCC resulted in product epimerization and so the method was abandoned. Success was achieved by use of IBX in DMSO, overnight, resulting in the recovery of a quantitative yield of **23** (Scheme 6). The amine was synthesized by first converting the primary alcohol to a tosylate under basic conditions affording **24** in a yield of 83%. The displacement of the tosyl group with an azide whilst heating the reaction at 80 °C allowed for the isolation of azide **25** in a good yield of 75%. Heating at higher temperatures resulted in product decomposition. Hydrogenation of the azide **25** at 4 atm whilst incorporating Pd/C as a catalyst furnished the desired amine **26** in quantitative yield. Performing the reaction at a lower pressure resulted in the recovery of starting material only.

With the two C_5_ fragments in hand, convergent coupling of aldehyde **23** and amine **26** was achieved via reductive amination employing sodium triacetoxyborohydride as the reducing agent. The desired product precursor **27** was successfully recovered as a yellow oil in a yield of 95%. HRMS showed the desired mass whilst NMR spectroscopy showing a single set of signals for each half of the structure, confirmed the desired *C*_2_ symmetry and stereochemical integrity. The ultimate deprotection step proved to be more difficult than expected. Ordinarily, there are a number of methods that can be employed to simultaneously deprotect the Boc and acetonide groups [27–29], but they could not effect the desired transformation in this case. After several failed attempts, a procedure typically used for Boc deprotection in peptide synthesis utilizing TFA and triisopropylsilane was successfully used. Product purification furnished the desired enantiomer of pavettamine as a TFA salt (**28**). The presence of TFA was evident in the ^13^C NMR spectrum due to the observed distorted quartet at δ 163.7 for the C=O group displaying ^2^*J*_CF_ coupling and a quartet at δ 120.3 with a ^1^*J*_CF_ coupling constant of 291 Hz. ^19^F NMR and HRMS further confirmed the presence of the recovered TFA salt of *ent*-pavettamine (**28**). However, attempts to neutralize the TFA salt to obtain the free amine were unsuccessful, most often resulting in product decomposition. Also the attempted product purification by recrystallization from methanol did not improve the purity of the product. Spectroscopic data of the synthesized *ent*-pavettamine and the pavettamine originally synthesized could unfortunately not be directly compared because the enantiomer was recovered as a TFA salt, whilst pavettamine was recovered as a neutral polyamine.

## Conclusion

*ent*-Pavettamine, isolated as the TFA salt, was successfully synthesized for the first time. Key steps in the route were the synthesis of the C_5_ intermediate **4** in twice the yield previously achieved, and the effective use of reductive amination for coupling of the two C_5_ units. The successful strategy employed was to functionalize the two C_5_ units at the opposite end to that previously described for the synthesis of pavettamine, in order to obtain the enantiomer. The final deprotection step proved particularly efficient, with two protecting groups being removed simultaneously to unveil the desired target.

## Supporting Information

Detailed experimental methods and NMR spectra for all compounds prepared and crystal structure data for compounds **4** and **9** is available as supporting information.

File 1 NMR data of all compounds.

File 2 Crystal structure data for compound **4**.

File 3 Crystal structure data for compound **9**.
