# Supplementary material for: Total synthesis of ent-pavettamine
Source: Beilstein J Org Chem. 2021 Jun 10;17:1440–6. doi: 10.3762/bjoc.17.99 (PMC8204129; doi:10.3762/bjoc.17.99)

BEILSTEIN JOURNAL OF ORGANIC CHEMISTRY

Supporting Information

for

Total synthesis of *ent*-pavettamine

Memory Zimuwandeyi, Manuel A. Fernandes, Amanda L. Rousseau and Moira L. Bode

Beilstein J. Org. Chem. **2021,** *17,* 1440–1446. doi:10.3762/bjoc.17.99

NMR data of all compounds

License and Terms: This is a supporting information file under the terms of the Creative Commons Attribution License (https://creativecommons.org/licenses/by/4.0). Please note that the reuse, redistribution and reproduction in particular requires that the author(s) and source are credited and that individual graphics may be subject to special legal provisions.
The license is subject to the *Beilstein Journal of Organic Chemistry* terms and conditions: (https://www.beilstein-journals.org/bjoc/terms)

Supporting Information

1. Experimental methods S2

2. NMR spectra

Compound **4** S16

Compound **5** S19

Compound **6** S21

Compound **7** S22

Compound **8** S23

Compound **9** S25

Compound **16** S27

Compound **17** S29

Compound **18** S32

Compound **19** S34

Compound **20** S36

Compound **21** S39

Compound **22** S41

Compound **23** S42

Compound **24** S43

Compound **25** S45

Compound **26** S47

Compound **27** S49

Compound **28** S51

Experimental

General

Reagents were purchased from Sigma-Aldrich and used without purification. Infrared spectra were recorded neat using a Bruker TENSOR 27 single channel infrared spectrometer. Melting points are uncorrected and were collected on a Stuart SMP 10 melting point apparatus. ^{1}H and ^{13}C NMR spectra were recorded using either a Bruker AVANCE 111 300, 400; or 500 MHz spectrometer in deuterated chloroform ($CDCl_3$) with trimethylsilane (TMS) as internal standard (δ = 0) for ^{1}H NMR, and $CDCl_3$ (δ = 77.0 ppm) for ^{13}C NMR. The chemical shift (δ) is reported in ppm and the coupling constants (*J*) in Hz. High resolution mass spectral data was collected on a Waters Synapt G2 using an ESI positive source and a cone voltage of 15 V. TLC was performed on aluminium-backed Merck silica gel 60 F_{254} plates. The purification of compounds was carried out using gravity column chromatography on silica gel 60 (particle size 0.063–0.200 mm) purchased from Merck. An Agilent 3000 instrument was used for all preparative HPLC on an Agilent prep C18 reverse phase column (21.2 × 20 mm) with a binary solvent system using gradient elution of CH_3CN/H_2O and a flow rate of 20 mL/min. For all analytical HPLC a Dionex Ultimate 3000 instrument was used, employing a Luna C18 reverseD phase column with gradient elution, at a flow rate of 1 mL/min and with UV detection at 217 nm.

Synthetic methods

(4*R*,6*S*)-2,2-Dimethyl-4-(((*S*)-*p*-tolylsulfinyl)methyl)-6-((trityloxy)methyl)-1,3-dioxane (4) [1]

Compound **17** (4.40 g, 8.79 mmol), 2,2-dimethoxypropane (30 mL, 19.2 mmol) and *p*-toluene sulfonic acid (125 mg) were dissolved in acetone (40 mL). The mixture was then left stirring for 1 h after which a few drops of Et_3N were added to neutralise the acid. Excess solvent was removed *in vacuo*, and a white precipitate was recovered. Purification of the desired product by column chromatography (EtOAc/Hex, 1:1) afforded intermediate **4** (4.08 g, 86%) as a white solid. **R*f*** = 0.46 (EtOAc/Hex, 1:1); **[α]** $_D^{21}$ = +28.1 (c 1.09, acetone); **M.p.** = 164 – 165 °C (Lit[1], **M.p.** = 167 – 168 °C); **IR:** ν_{max}(cm^{-1}): 3036 (C=C-H), 2925 (C-H), 1597 (C=C), 1161, 1100 and 1078 (C-O), 1047 (S=O); **^{1}H NMR (500 MHz, $CDCl_3$)** δ 7.55 – 7.52 (m, 2H), 7.45 – 7.41 (m, 6H),

7.33 – 7.19 (m, 11H), 4.13 – 4.06 (m, 1H), 3.97 (dtd, *J* = 11.4, 5.6, 2.4 Hz, 1H), 3.23 (dd, *J* = 9.3, 5.2 Hz, 1H), 3.15 (dd, *J* = 13.1, 6.8 Hz, 1H), 2.96 (dd, *J* = 9.3, 6.0 Hz, 1H), 2.76 (dd, *J* = 13.1, 5.4 Hz, 1H), 2.41 (s, 3H), 1.75 (dt, *J* = 12.8, 2.5 Hz, 1H), 1.40 – 1.34 (m, 1H), 1.33 (s, 3H), 1.30 (s, 3H); **^{13}C NMR (126 MHz, $CDCl_3$)** δ 143.9, 141.6, 140.3, 129.8, 128.7, 127.8, 127.0, 124.4, 98.9, 86.5, 68.2, 67.0, 63.9, 63.1, 33.6, 29.7, 21.4, 19.6.

(*S*)-Methyl 3,4-dihydroxybutanoate (5)

To a stirring solution of (*S*)-dimethyl 2-hydroxysuccinate (17.75 g, 0.109 mol) in THF (300 mL) was added BMS (12 mL, 0.120 mol) dropwise over 4 h. The reaction mixture was cooled to 0 °C in an ice bath, followed by the addition of $NaBH_4$ (53.90 mg, 1.425 mmol). After stirring for 10 min, the reaction was warmed to rt and stirred for an additional 1 h. Upon completion, the dropwise addition of MeOH (100 mL) and solid *p*-TsOH quenched the reaction. The reaction mixture was concentrated and purified by column chromatography (EtOAc), yielding diol **5** (13.34 g, 91%), as a colourless oil. **R_f** = 0.30 (EtOAc); **$[\alpha]_D^{20}$** -23.7 (c 1.83, $CHCl_3$); **IR:** ν_{max}(cm^{-1}): 3372 (OH), 2955 (C-H), 1719 (C=O), 1167 (C-O); **^{1}H NMR (500 MHz, $CDCl_3$)** δ 4.28 (d, *J* = 4.7 Hz, 1H), 4.16 – 4.09 (m, 1H), 4.01 (t, *J* = 5.9 Hz, 1H) 3.71 (s, 3H), 3.66 – 3.60 (m, 1H), 3.54 – 3.47 (m, 1H), 2.56 – 2.46 (m, 2H); **^{13}C NMR (126 MHz, $CDCl_3$)** δ 172.8, 68.8, 65.8, 51.9, 37.9.

(*S*)-Methyl 3-hydroxy-4-(trityloxy)butanoate (6) [2]

Diol **5** (3.77 g, 28.1 mmol) was dissolved in pyridine (9 mL) and DCM (30 mL) followed by the addition of DMAP (1.72 g, 14.1 mmol) and trityl chloride (8.62 g, 30.9 mmol), and the reaction was left stirring overnight. TLC analysis showed total consumption of the starting material. The reaction mixture was washed with 1 M HCl (4 × 100 mL) followed by saturated brine (200 mL). The organic layer was dried over Na_2SO_4 and excess solvent was removed *in vacuo*. Purification of the product by column chromatography (EtOAc/Hex, 3:7) afforded compound **6** (10.55 g, 100%) as a yellow oil. **R_f** = 0.42 (EtOAc/Hex, 3:7); **$[\alpha]_D^{20}$** = -15.0 (c 1.05, $CHCl_3$); **M.p**: 129 – 130 °C **IR**: ν_{max}(cm^{-1}): 3469 (OH), 3058 (C=C-H), 2951 (C-H), 1732 (C=O), 1597 (C=C), 1216 (C-O); **^{1}H NMR (300 MHz, $CDCl_3$)** δ 7.47 – 7.39 (m, 6H), 7.33 – 7.16 (m, 9H), 4.28 – 4.16 (m,

1H), 3.63 (s, 3H), 3.16 (d, *J* = 5.4 Hz, 2H), 3.02 (d, *J* = 4.6 Hz, 1H), 2.64 – 2.43 (m, 2H); **^{13}C NMR (75 MHz, $CDCl_3$)** δ 172.5, 143.7, 128.4, 127.7, 126.9, 86.7, 67.5, 66.6, 51.7, 38.4; **HRMS** (m/z), calculated for $C_{24}H_{24}NaO_4$: 399.1567, found $(M+Na)^+$: 399.1561.

(*S*)-Methyl 3-((*tert*-butyldimethylsilyl)oxy)-4-(trityloxy)butanoate (7) [2]

To a stirring solution of compound **6** (10.40 g, 27.8 mmol) in DCM was added, 1.5 equiv of TBS (5.03 g, 33.4 mmol), imidazole (1.5 equiv, 2.84 g, 41.7 mmol) and DMAP (5.12 g, 41.7 mmol) sequentially. TLC showed total consumption of the starting material after 12 h. Work up of the reaction by washing the reaction mixture with H_2O (300 mL), saturated brine (300 mL), and drying the organic layer over Na_2SO_4, followed by the removal of excess solvent *in vacuo* afforded the crude product. Purification was achieved by column chromatography (EtOAc/Hex, 1:9) yielding compound **7** (8.82 g, 65%) as a white precipitate. **R_f** = 0.38 (EtOAc/Hex, 1:9); **$[\alpha]_D^{20}$** = -13.4 (c 1.00, $CHCl_3$); **M.p**: 70 – 71 °C **IR**: ν_{max}(cm^{-1}): 3058 (C=C-H), 2928 (C-H), 1738 (C=O), 1598 (C=C), 1123, 1074 and 1031 (C-O); **^{1}H NMR (300 MHz, $CDCl_3$)** δ 7.49 – 7.38 (m, 6H), 7.35 – 7.16 (m, 9H), 4.31 – 4.19 (m, 1H), 3.63 (s, 3H), 3.18 – 3.09 (m, 1H), 3.06 – 2.96 (m, 1H), 2.75 (dd, *J* = 14.9, 4.5 Hz, 1H), 2.47 (dd, *J* = 14.9, 8.1 Hz, 1H), 0.80 (s, 9H), -0.02 (s, 3H), -0.08 (s, 3H); **^{13}C NMR (75 MHz, $CDCl_3$)** δ 172.2, 143.9, 128.7, 127.8, 127.0, 86.7, 68.9, 67.2, 51.4, 40.7, 25.7, 17.9, -4.6, -5.2; **HRMS** (m/z), calculated for $C_{30}H_{38}NaO_4Si$: 513.2432, found $(M+Na)^+$: 513.2422.

Sulfoxide addition to (*S*)-methyl 3-((*tert*-butyldimethylsilyl)oxy)-4-(trityloxy)butanoate (7)

LDA (1.0 M in hexanes, 26 mL, 26.0 mmol) was added to dry THF (100 mL) under N_2 at −78 °C, followed by the addition of *R*-(+)-methyl *p*-tolyl sulfoxide (3.21 g, 20.8 mmol) in dry THF (50 mL). The reaction proceeded for 30 min, during which the solution turned bright orange whilst the temperature was slowly raised to −40 °C. In a separate two-necked flask, (*S*)-methyl 2-(2,2-dimethyl-1,3-dioxolan-4-yl)acetate (**7**, 5.10 g, 10.4 mmol) was dissolved in dry THF (100 mL), and cooled to −40 °C. The methyl sulfoxide solution was added dropwise using a canula needle to the stirring solution of compound **7**, and the reaction was allowed to reach rt upon complete addition. After 2 h of stirring at rt the reaction was quenched by the addition

of saturated aqueous NH_4Cl solution (200 mL), and acidified to pH 6 using 1 M HCl. The mixture was extracted with EtOAc (3 × 100 mL). The combined organic layers were washed with H_2O (100 mL), followed by saturated brine solution (100 mL), and dried over anhydrous Na_2SO_4. TLC showed two products of potential significance. Excess solvent was removed *in vacuo* yielding a brownish oil, which was purified by column chromatography. Gradient elution was used for separating the desired products starting from (EtOAc/Hex, 1:1) and then increasing the ratio to 4:1. The separated side product **9** (1.50 g, 24%), and compound **8** (3.49 g, 55%) were recovered as pale yellow solids.

(*S*)-4-((*tert*-Butyldimethylsilyl)oxy)-1-((*R*)-*p*-tolylsulfinyl)-5-(trityloxy)pentan-2-one (8)

R_f = 0.46 (EtOAc/Hex, 3:7); **$[\alpha]_D^{20}$** = +67.0 (c 1.3, $CHCl_3$); **M.p**. = 115 – 116 °C; **IR**: $\nu_{max}(cm^{-1})$: 3064 (C=C-H), 2950 (C-H), 1712 (C=O), 1080 (C-O), 1050 (S=O); **^1H NMR (300 MHz, $CDCl_3$)** δ 7.45 (d, *J* = 7.9 Hz, 2H), 7.42 – 7.36 (m, 6H), 7.32 – 7.17 (m, 11H), 4.28 – 4.17 (m, 1H), 3.88 (d, *J* = 14.0 Hz, 1H), 3.76 (d, *J* = 14.0 Hz, 1H), 3.10 (dd, *J* = 9.3, 4.6 Hz, 1H), 2.95 (dd, *J* = 9.3, 6.8 Hz, 1H), 2.80 – 2.62 (m, 2H), 2.36 (s, 3H), 0.79 (s, 9H), -0.02 (s, 3H), -0.09 (s, 3H); **^{13}C NMR (75 MHz, $CDCl_3$)** δ 200.5, 143.7, 142.0, 140.0, 130.0, 128.6, 127.8, 127.1, 124.1, 86.9, 69.9, 68.3, 67.1, 50.0, 25.7, 21.4, 17.9, -4.7, -5.0; **HRMS** (m/z), calculated for $C_{37}H_{44}NaO_4SSi$: 635.2622, found $(M+Na)^+$: 635.2616.

(*S*)-3-((*tert*-Butyldimethylsilyl)oxy)-1-((*R*)-*p*-tolylsulfinyl)-5-(trityloxy)pentan-2-one (9)

R_f = 0.48 (EtOAc/Hex, 3:7); **$[\alpha]_D^{20}$** = +91.4 (c 1.3, $CHCl_3$); **M.p.** = 115 – 116 °C; **IR**: $\nu_{max}(cm^{-1})$: 3056 (C=C-H), 2951 (C-H), 1716 (C=O), 1083 (C-O), 1067 (S=O); **^1H NMR (300 MHz, $CDCl_3$)** δ 7.56 – 7.50 (m, 2H), 7.41 – 7.34 (m, 6H), 7.33 – 7.16 (m, 11H), 4.18 (t, *J* = 5.5 Hz, 1H), 4.07 (d, *J* = 14.8 Hz, 1H), 3.92 (d, *J* = 14.8 Hz, 1H), 3.32 – 3.18 (m, 1H), 3.17 – 3.04 (m, 1H), 2.40 (s, 3H), 2.12 – 1.95 (m, 1H), 1.90 – 1.75 (m, 1H), 0.75 (s, 9H), -0.07 (s, 3H), -0.13 (s, 3H); **^{13}C NMR (75 MHz, $CDCl_3$)** δ 204.7, 143.8, 141.9, 141.0, 130.0, 128.6, 127.8, 127.0, 124.3, 87.3, 76.2, 65.3, 58.7, 34.5, 25.7, 21.4, 17.9, -5.0, -5.1; **HRMS** (m/z), calculated for $C_{37}H_{44}NaO_4SSi$: 635.2622, found $(M+Na)^+$: 635.2621.

(2*R*,4*S*)-4-((*tert*-Butyldimethylsilyl)oxy)-1-((*R*)-*p*-tolylsulfinyl)-5-(trityloxy)pentan-2-ol (16)

$ZnCl_2$ (3.25 g, 23.8 mmol) was flame dried under vacuum in a 500 mL 2-necked round-bottomed flask, cooled and dissolved in dry THF (200 mL). (*S*)-4-((*tert*-butyldimethylsilyl)oxy)-1-((*R*)-*p*-tolylsulfinyl)-5-(trityloxy)pentan-2-one (**8**, 3.65 g, 5.96 mmol) was dissolved in dry THF (100 mL) and added to the $ZnCl_2$ solution and the mixture was left stirring under N_2 at rt for 2 h. The reaction mixture was cooled to −78 °C, left stirring for 10 min followed by the dropwise addition of DIBALH (1.0 M, 9.0 mL, 9.0 mmol). After the complete addition of the DIBALH, the stirring reaction mixture was warmed to rt and allowed to proceed for a further 2 h after which the temperature was reduced to −78 °C. The reaction was then quenched by the slow addition of a saturated aqueous solution of NH_4Cl until effervescence had stopped. Excess solvent was removed *in vacuo* and the residue was extracted with EtOAc (3 × 150 mL). The combined organic layers were washed with saturated brine (150 mL), dried over anhydrous Na_2SO_4 and an oily residue was recovered after removal of solvent. Purification of the desired compound was afforded by column chromatography (EtOAc/Hex, 3:7) yielding the compound **16** (3.20 g, 87%) as a yellow solid. **R_f** = 0.27 (EtOAc/Hex, 3:7); **$[\alpha]_D^{20}$** = +43.0 (c 1.3, $CHCl_3$); **M.p.** = 45 – 46 °C; **IR**: ν_{max}(cm^{-1}): 3339 (OH), 3058 (C=C-H), 2952 (C-H), 1251, 1155, 1083 (C-O), 1029 (S=O); **^{1}H NMR (500 MHz, $CDCl_3$)** δ 7.55 (d, *J* = 8.2 Hz, 2H), 7.46 – 7.38 (m, 6H), 7.35 – 7.19 (m, 11H), 4.24 – 4.07 (m, 1H), 3.97 – 3.87 (m, 2H), 3.15 – 3.00 (m, 3H), 2.77 (dd, *J* = 13.0, 3.1 Hz, 1H), 2.42 (s, 3H), 1.95 (dt, *J* = 14.2, 4.7 Hz, 1H), 1.89 – 1.77 (m, 1H), 0.80 (s, 9H), -0.04 (s, 3H), -0.10 (s, 3H); **^{13}C NMR (126 MHz, $CDCl_3$)** δ 143.8, 141.8, 140.7, 130.0, 128.7, 127.9, 127.1, 124.2, 86.9, 70.2, 67.3, 66.3, 63.7, 41.7, 25.8, 21.5, 17.9, -4.4, -4.9; **HRMS** (m/z), calculated for $C_{37}H_{46}NaO_4SSi$: 637.6722, found $(M+Na)^+$: 637.6729.

(2*R*,4*S*)-1-((*S*)-*p*-Tolylsulfinyl)-5-(trityloxy)pentane-2,4-diol (17) [1]

To a solution of compound **16** (2.90 g, 4.71 mmol) in dry THF (150 mL) was added TBAF (1.5 equiv, 7.00 mL) under N_2 at 0 °C. The reaction was stirred for 4 h until completion, whilst monitoring by TLC. Quenching of the reaction was achieved by addition of saturated aqueous NH_4Cl solution (50 mL), followed by washing with saturated brine (100 mL). The organic layer

was separated and dried over anhydrous Na_2SO_4 and excess solvent was removed *in vacuo*. Purification of the desired product using column chromatography (EtOAc/Hex, 4:1) yielded the desired product as a white precipitate, compound **17** (2.08 g, 87%). **R$_f$** = 0.38 (EtOAc/Hex, 4:1); **[α]$_D^{21}$** = +118.6 (c 0.98, acetone), **[α]$_D^{20}$** = +83.3 (c 1.00, $CHCl_3$); **M.p.** = 65 – 66 °C (Lit[1], **M.p.** = 74 – 76 °C); **IR:** ν_{max}(cm^{-1}): 3355 (OH), 3056 (C=C-H), 2918 (C-H), 1596 (C=C), 1221 (C-O), 1083 (C-O), 1030 (S=O); **^{1}H NMR (500 MHz, CDCl$_3$)** δ 7.55 – 7.52 (m, 2H), 7.43 – 7.37 (m, 6H), 7.35 – 7.20 (m, 11H), 4.42 – 4.35 (m, 1H), 4.31 (s, 1H), 4.08 – 4.01 (m, 1H), 3.15 (d, *J* = 2.7 Hz, 1H), 3.11 (d, *J* = 5.6 Hz, 2H), 3.03 (dd, *J* = 13.2, 8.2 Hz, 1H), 2.79 (dd, *J* = 13.2, 3.4 Hz, 1H), 2.41 (s, 3H), 1.77 – 1.67 (m, 2H); **^{13}C NMR (126 MHz, CDCl$_3$)** δ 143.7, 141.9, 140.5, 130.1, 128.6, 127.9, 127.1, 124.1, 86.8, 70.6, 68.3, 67.4, 62.9, 39.4, 21.4.

(4*R*,6*S*)-2,2-Dimethyl-6-((trityloxy)methyl)-1,3-dioxane-4-carbaldehyde (18)

Compound **4** (3.38 g, 6.26 mmol) was dissolved in dry MeCN (20 mL) followed by the subsequent addition of collidine (2.48 mL, 18.77 mmol) and TFAA (4.35 mL, 31.28 mmol) at 0 °C. The reaction proceeded for 30 min until total consumption of the starting material was detected, before quenching with water (1 mL) followed by solid K_2CO_3 until the reaction mixture was neutral. Stirring of the mixture was continued for a further 30 min at room temperature after which it was diluted with EtOAc (250 mL), washed with saturated aqueous NH_4Cl solution (200 mL), 1 M HCl (100 mL), saturated $NaHCO_3$ solution (200 mL) and saturated brine solution (250 mL). The organic layer was separated, and dried over anhydrous Na_2SO_4 and excess solvent was removed *in vacuo*. Purification of the desired product was achieved by column chromatography employing gradient elution (EtOAc/Hex, 1:9 to 1:1) yielding compound **18** (2.59 g, 99%), as a white solid. **R$_f$** = 0.30 (EtOAc/Hex, 1:1); **M.p.**: 61 – 62 °C; **IR**: ν_{max}(cm^{-1}): 3059 (C=C-H), 2991 (C-H), 1736 (C=O), 1258, 1165, 1076 (C-O) **^{1}H NMR (300 MHz, CDCl$_3$)** δ 9.58 (s, 1H), 7.50 – 7.40 (m, 6H), 7.34 – 7.17 (m, 9H), 4.30 (dd, *J* = 12.3, 3.0 Hz, 1H), 4.09 – 3.96 (m, 1H), 3.26 (dd, *J* = 9.4, 5.2 Hz, 1H), 3.02 (dd, *J* = 9.4, 6.1 Hz, 1H), 1.88 (dt, *J* = 13.0, 2.8 Hz, 1H), 1.462 (s, 3H), 1.457 (s, 3H), 1.43 – 1.32 (m, 1H); **^{13}C NMR (75 MHz, CDCl$_3$)** δ 201.1, 143.9, 128.7, 127.8, 127.0, 99.0, 86.6, 73.8, 67.9, 66.9, 29.6, 28.6, 19.4.

N-Benzyl-1-((4R,6S)-2,2-dimethyl-6-((trityloxy)methyl)-1,3-dioxan-4-yl)methanamine (19)

A solution of benzylamine (774 µL, 7.08 mmol) in 1,2-dichloroethane (18 mL) was added to a solution of compound **18** (2.27 g, 5.44 mmol) in THF (2 mL) with stirring under N_2. The mixture was treated with sodium triacetoxyborohydride (1.61 g, 7.61 mmol) in one portion and the reaction was allowed to proceed for 24 h whilst monitoring by TLC. Quenching of the reaction was achieved by addition of 1 M NaOH (20 mL) followed by extraction of the organic layer with DCM (100 mL). The organic layer was washed with saturated brine solution (100 mL), and dried over anhydrous Na_2SO_4, followed by solvent removal *in vacuo.* Purification by column chromatography (EtOAc/Hex, 1:1) yielded the desired product **19** (1.65 g, 60%) as a colourless thick gel. **R_f** = 0.38 (EtOAc/Hex, 1:1); **$[\alpha]_D^{20}$** = -16.8 (c 0.9, $CHCl_3$); **IR**: ν_{max}(cm^{-1}): 3316 (NH), 3060 (C=C-H), 2986 (C-H), 1092 (C-O); **^{1}H NMR (400 MHz, $CDCl_3$)** δ 7.54 – 7.48 (m, 6H), 7.40 – 7.24 (m, 14H), 4.16 – 4.00 (m, 2H), 3.85 (s, 2H), 3.30 (dd, *J* = 9.2, 5.3 Hz, 1H), 3.02 (dd, *J* = 9.2, 6.0 Hz, 1H), 2.77 – 2.66 (m, 2H), 2.08 (br-s, 1H), 1.64 (dt, *J* = 12.8, 2.5 Hz, 1H), 1.50 (s, 3H), 1.44 (s, 3H), 1.36 – 1.23 (m, 1H); **^{13}C NMR (75 MHz, $CDCl_3$)** δ 144.0, 140.3, 128.7, 128.3, 128.1, 127.7, 126.9, 98.6, 86.4, 68.34, 68.27, 67.3, 54.6, 53.9, 32.1, 30.0, 19.9; **HRMS** (m/z), calculated for $C_{14}H_{37}NO_3$: 508.2846, found $(M+H)^+$: 508.2865.

((4R,6S)-2,2-Dimethyl-6-((trityloxy)methyl)-1,3-dioxan-4-yl)methanamine (20) [1]

Compound **19** (1.04 g, 2.05 mmol) was dissolved in ethanol (10 mL) and left stirring under N_2 for 15 min, followed by the addition of Pd/C (1.00 g, 10%). The reaction was stirred for a further 15 min before introducing H_2. Completion of the reaction was observed after 24 h of regular monitoring using TLC. The mixture was filtered through a celite plug and excess solvent was removed *in vacuo*. Purification of the desired product was achieved by column chromatography (DCM/MeOH, 95:5) yielding amine **20** (0.71 g, 84%) as a white gluey viscous oil. **R_f** = 0.27 (DCM/MeOH, 1:1); **$[\alpha]_D^{20}$** = -24.6 (c 1.0, $CHCl_3$); **IR**: ν_{max}(cm^{-1}): 3319 (NH), 3057 (C=C-H), 2924 (C-H), 1052 (C-O); **^{1}H NMR (300 MHz, $CDCl_3$)** ^{1}H NMR (300 MHz, $CDCl_3$) δ 7.48 – 7.42 (m, 6H), 7.32 – 7.18 (m, 9H), 4.03 (dtd, *J* = 11.5, 5.6, 2.5 Hz, 1H), 3.92 – 3.78 (m, 1H), 3.25 (dd, *J* = 9.2, 5.3 Hz, 1H), 2.96 (dd, *J* = 9.2, 6.0 Hz, 1H), 2.80 – 2.62 (m, 2H), 1.65 (br-s, 2H), 1.58 (dt, *J* = 12.7, 2.5 Hz, 1H), 1.45 (s, 3H), 1.39 (s, 3H), 1.29 – 1.10 (m, 1H), **^{13}C NMR (75 MHz,**

$CDCl_3$) δ 144.0, 128.7, 127.7, 126.9, 98.6, 86.5, 70.5, 68.2, 67.3, 47.4, 31.6, 30.0, 19.9; **HRMS** (m/z), calculated for $C_{27}H_{32}NO_3$: 418.2377, found $(M+H)^+$: 418.2376.

tert-Butyl (((4*R*,6*S*)-2,2-dimethyl-6-((trityloxy)methyl)-1,3-dioxan-4-yl)methyl)carbamate (21)

Compound **20** (0.68 g, 1.63 mmol) was dissolved in THF (50 mL), followed by the addition of Boc anhydride (0.43 g, 1.96 mmol) and DMAP (10 mg, 0.082 mmol). The reaction was left stirring overnight and TLC showed total consumption of the starting material. Work up of the experimental mixture was achieved by diluting with DCM (100 mL) followed by washing with H_2O (100 mL) and saturated brine solution (150 mL). The organic layer was separated, dried over anhydrous Na_2SO_4 and excess solvent was removed *in vacuo*. The desired product **21** was recovered as a white solid (0. 85 g, 100%). $\mathbf{R_f}$ = 0.30 (EtOAc/Hex, 1:9); $[\alpha]_D^{20}$ = -20.3 (c 1.0, $CHCl_3$); IR: $v_{max}(cm^{-1})$: 3330 (NH), 3057 (C=C-H), (C-H), 1686 (C=O), 1091 (C-O); **^{1}H NMR (300 MHz, $CDCl_3$)** δ 7.49 – 7.40 (m, 6H), 7.34 – 7.13 (m, 9H), 4.88 (br-s, 1H), 4.11 – 3.89 (m, 2H), 3.40 – 3.19 (m, 2H), 3.07 – 2.91 (m, 2H), 1.58 (dt, *J* = 12.8, 2.5 Hz, 1H), 1.45 (s, 9H), 1.43 (s, 3H), 1.38 (s, 3H), 1.28 – 1.13 (m, 1H); **^{13}C NMR (75 MHz, $CDCl_3$)** δ 156.0, 144.0, 128.7, 127.8, 127.0, 98.7, 86.5, 79.3, 68.2, 68.1, 67.2, 45.5, 31.2, 29.9, 28.4, 19.9; **HRMS** (m/z), calculated for $C_{32}H_{39}NNaO_5$: 540.2720, found $(M+Na)^+$: 540.2711.

tert-Butyl (((4*R*,6*S*)-6-(hydroxymethyl)-2,2-dimethyl-1,3-dioxan-4-yl)methyl)carbamate (22)

A 500 mL two-necked round-bottomed flask was heat sealed before being connected to a cold finger condenser under N_2. Na (729 mg, 31.7 mmol) was added in one portion and the flask was cooled to −60 °C with the condenser also at −60 °C. Liquid NH_3 (50 mL) was introduced to the flask and it was allowed to reflux for 15 min ensuring that the colour remained deep blue. Compound **21** (820 mg, 1.58 mmol) was dissolved in dry THF (20 mL), cooled, and added to the ammonia solution. There was no colour change observed, and the reaction proceeded for a further 1 h. Quenching was achieved by slow addition of ethanol (10 mL) over 10 min until the blue colour had disappeared completely. Solid NH_4Cl (4 g) was

then added in one portion and the reaction mixture was left stirring open at ambient temperature until all the NH_3 had evaporated. The mixture was diluted with saturated aqueous NH_4Cl solution (100 mL), and the organic layer was extracted with DCM (200 mL), dried over anhydrous Na_2SO_4 and excess solvent was removed *in vacuo*. Purification of the desired compound was achieved by column chromatography (DCM/MeOH, 95:5) yielding compound **22** (353 mg, 81%) as a yellow oil. **R_f** = 0.30 (DCM/MeOH, 95:5); **$[\alpha]_D^{20}$** = -10.7 (c 0.6, $CHCl_3$); **IR**: ν_{max}(cm^{-1}): 3324-br (OH and NH), 2923 (C-H), 1023 (C-O); **^{1}H NMR (300 MHz, $CDCl_3$)** δ 4.95 (br-s, 1H), 4.03 – 3.95 (m, 2H), 3.60 (dd, *J* = 11.5, 3.4 Hz, 1H), 3.51 (dd, *J* = 11.5, 6.2 Hz, 1H), 3.36 – 3.27 (m, 1H), 3.04 (ddd, *J* = 13.8, 6.9, 5.1 Hz, 1H), 2.41 (br-s, 1H), 1.47 – 1.43 (m, 12H), 1.40 (s, 3H), 1.40 – 1.30 (m, 2H), **^{13}C NMR (75 MHz, $CDCl_3$)** δ 156.1, 98.8, 79.4, 69.3, 67.9, 65.9, 45.4, 29.9, 29.2, 28.4, 19.9; **HRMS** (m/z), calculated for $C_{13}H_{26}NO_5$: 276.1805, found $(M+H)^+$: 276.1806.

tert-Butyl (((4*R*,6*S*)-6-formyl-2,2-dimethyl-1,3-dioxan-4-yl)methyl)carbamate (23)

Compound **22** (196 mg, 0.712 mmol) and IBX (797 mg, 2.85 mmol) were added to DMSO (10 mL) and left stirring overnight. TLC analysis showed formation of an aldehyde using 2,4-DNP as a visualisation agent. The reaction mixture was diluted with DCM (100 mL), followed by washing with water (2 × 100 mL), saturated brine (100 mL) and evaporation of excess solvent. Quantitative yield of the crude aldehyde **23** was recovered as an oil and was not purified further. **R_f** = 0.34 (DCM/MeOH, 95:5); **IR**: ν_{max}(cm^{-1}): 3382 (NH), 2922 (C-H), 1710 (C=O), 1164 (C-O); **^{1}H NMR (300 MHz, $CDCl_3$)** δ 9.58 (s, 1H), 4.93 (s, 1H), 4.49 (dd, *J* = 12.2, 2.9 Hz, 0.34H) and 4.32 (dd, *J* = 12.2, 3.1 Hz, 0.41H), 4.15 – 3.84 (s, 1H), 3.43 – 3.18 (m, 1H), 3.15 – 3.00 (m, 1H), 1.77 – 0.99 (m, 17H; **HRMS** (m/z), calculated for $C_{13}H_{24}NO_5$: 274.1649, found $(M+H)^+$: 274.1647.

((4*S*,6*R*)-6-(((*tert*-Butoxycarbonyl)amino)methyl)-2,2-dimethyl-1,3-dioxan-4-yl)methyl 4-methylbenzenesulfonate (24)

Compound **22** (198 mg, 0.719 mmol), was dissolved in DCM (10 mL), followed by the addition of DMAP (527 mg, 4.31 mmol) and TsCl (822 mg, 4.31 mmol). The reaction was left stirring for 24 h until TLC showed complete disappearance of the starting material. Work up of the reaction was performed by diluting the reaction with water (100 mL), followed by extraction of the desired compound with DCM (150 mL), followed by washing with saturated brine (100 mL). The organic layer was separated and dried over anhydrous Na_2SO_4 and excess solvent was removed *in vacuo*. Purification of the product was achieved by column chromatography (EtOAc/Hex, 3:7) yielding compound **24** (256 mg, 83%) as a yellow oil. **R_f** = 0.32 (EtOAc/Hex, 3:7); **$[\alpha]_D^{20}$** = -3.2 (c 1.02, $CHCl_3$); **IR**: ν_{max}(cm^{-1}): 3403 (NH), 2980 (C-H), 1598 (C=C), 1363 (S=O), 1168 (C-N), 1097 (C-O); **^{1}H NMR (300 MHz, $CDCl_3$)** δ 7.78 (d, *J* = 8.3 Hz, 2H), 7.34 (d, *J* = 7.9 Hz, 2H), 4.91 (br-s, 1H), 4.17 – 4.04 (m, 1H), 4.02 – 3.85 (m, 3H), 3.34 – 3.19 (m, 1H), 3.02 (ddd, *J* = 13.8, 6.7, 5.3 Hz, 1H), 2.45 (s, 3H), 1.44 (s, 9H), 1.36 (s, 3H), 1.30 (s, 3H), 1.28 – 1.10 (m, 2H); **^{13}C NMR (75 MHz, $CDCl_3$)** δ 156.0, 144.9, 132.9, 129.8, 128.0, 99.0, 79.4, 72.2, 67.7, 66.6, 45.2, 29.6, 29.5, 28.4, 21.6, 19.6.

***tert*-Butyl (((4*R*,6*S*)-6-(azidomethyl)-2,2-dimethyl-1,3-dioxan-4-yl)methyl)carbamate (25)**

Compound **24** (240 mg, 0.559 mmol) was dissolved in DMF (6 mL) followed by the addition of NaN_3 (182 mg, 2.79 mmol) and the reaction was refluxed at 80 °C for 3 h. After TLC analysis showed complete disappearance of the starting material the reaction was cooled and diluted with Et_2O (100 mL) and washed with saturated brine. The brine layer in turn was extracted once with Et_2O (100 mL). The combined Et_2O solutions were washed with saturated brine (7 × 150 mL), dried over Na_2SO_4 and evaporated to afford the crude product. Purification by column chromatography (EtOAc/Hex, 3:7) yielded the desired product **25** (125 mg, 75%) as a yellow oil. **R_f** = 0.35 (EtOAc/Hex, 3:7); **$[\alpha]_D^{20}$** = -5.4 (c 0.5, $CHCl_3$); **IR**: ν_{max}(cm^{-1}): 3358 (NH), 2979 (C-H), 2098 (N=N=N), 1703 (C=O), 1164 (C-O); **^{1}H NMR (300 MHz, $CDCl_3$)** δ 4.92 (br s, 1H), 4.12 – 3.91 (m, 2H), 3.40 – 3.15 (m, 3H), 3.06 (ddd, *J* = 13.9, 6.9, 5.2 Hz, 1H), 1.50 – 1.43 (m, 12H), 1.41 (s, 3H), 1.41 – 1.23 (m, 2H); **^{13}C NMR (75 MHz, $CDCl_3$)** δ 156.1, 99.1, 79.4, 68.2,

68.0, 55.1, 45.3, 30.6, 29.8, 28.4, 19.8; **HRMS** (m/z), calculated for $C_{13}H_{24}NaN_4O_4$: 323.1690, found $(M+H)^+$: 323.1691.

***tert*-Butyl (((4*R*,6*S*)-6-(aminomethyl)-2,2-dimethyl-1,3-dioxan-4-yl)methyl)carbamate (26)**

Pd/C (30 mg, 10 mol %) was added to a stirring solution of compound **25** (112 mg, 373 μmol) in ethanol (5 mL) under H_2. The reaction was allowed to proceed overnight at 4 atm until TLC showed total consumption of the starting material. The reaction mixture was then filtered over celite and evaporated yielding the desired amine **26** as a brown oil in quantitative yield. It was not purified any further. **R*f*** = 0.14 (DCM/MeOH, 9:1); **IR**: ν_{max}(cm^{-1}): 3353 (NH), 2978 (C-H), 1696 (C=O), 1160 (C-O); **^{1}H NMR (300 MHz, $CDCl_3$)** δ 5.00 (br-s, 1H), 4.09 – 3.91 (m, 1H), 3.89 – 3.79 (m, 1H), 3.39 – 3.22 (m, 1H), 3.04 (ddd, *J* = 13.8, 6.9, 5.0 Hz, 1H), 2.80 – 2.52 (m, 2H), 1.45 (s, 12H), 1.41 – 1.35 (m, 4H), 1.31 – 1.15 (m, 1H); **^{13}C NMR (75 MHz, $CDCl_3$)** δ 156.1, 98.8, 79.3, 70.3, 68.1, 47.4, 45.4, 30.9, 30.0, 28.4, 20.0; **HRMS** (m/z), calculated for $C_{13}H_{27}N_2O_4$: 275.1965, found $(M+H)^+$: 275.1975.

Di-*tert*-butyl (((4*R*,4'*R*,6*S*,6'*S*)-6,6'-(azanediylbis(methylene))bis(2,2-dimethyl-1,3-dioxane-6,4-diyl))bis(methylene))dicarbamate (27)

Aldehyde **23** (101 mg, 370 μmol) and amine **26** (84 mg, 307 μmol) were dissolved in THF (3 mL) under N_2 followed by the addition of 1,2-dichloroethane (5 mL) and sodium triacetoxyborohydride (109 mg, 518 μmol) in one portion. After stirring at room temperature for 24 h over molecular sieves (3 Å) the reaction was quenched with 1 M NaOH (5 mL), washed with DCM (50 mL) and saturated brine (50 mL). The organic layer was separated and dried over anhydrous Na_2SO_4 and excess solvent was removed *in vacuo*. Purification by column chromatography ($CHCl_3$/MeOH, 9:1) yielded compound **27** (155 mg, 95%) as a viscous yellow oil. **R*f*** = 0.40 (DCM/MeOH, 9:1); **$[\alpha]_D^{20}$** = -10.1 (c 1.03, $CHCl_3$); **IR** ν_{max}(cm^{-1}) : 3362 (NH), 2977 (C-H), 1694 (C=O), 1163 (C-O); **^{1}H NMR (500 MHz, $CDCl_3$)** δ 4.94 (s, 1H), 4.05 – 3.91 (m, 4H), 3.36 – 3.23 (m, 2H), 3.08 – 2.99 (m, 2H), 2.75 – 2.55 (m, 4H), 2.07 (br s, 2H), 1.48 – 1.42 (m, 26H), 1.38 (s, 3H), 1.30 – 1.21 (m, 1H); **^{13}C NMR (126 MHz, $CDCl_3$)** δ 156.1, 98.8, 79.3, 68.2,

67.9, 55.0, 45.4, 31.6, 30.0, 28.4, 19.9; **HRMS** (m/z), calculated for $C_{26}H_{50}N_3O_8$: 532.3592, found $(M+H)^+$: 532.3605.

(2*R*,2'*R*,4*S*,4'*S*)-5,5'-azanediylbis(1-aminopentane-2,4-diol) TFA salt (28)

Compound **27** (65 mg, 122 µmol) was dissolved in a solution TFA/H_2O/triisopropylsilane mixture (95%:2.5%:2.5%, 3 mL) and left stirring at ambient temperature. The reaction mixture was diluted with MeOH (100 mL) and excess solvent was removed under reduced pressure. The residue was redissolved in methanol (50 mL) followed by the addition of a few drops of Et_3N. The excess solvent was removed *in vacuo*. The desired product **28** was not purified any further and it was recovered as a yellow oil in quantitative yield. **R_f** = 0.35 (IPA/AcOH/H_2O, 9:1); **$[\alpha]_D^{20}$** = -1.61 (c 0.13, MeOH); **IR**: ν_{max}(cm^{-1}): 3220 – 2810 (NH_2, OH and C-H); **^{1}H NMR (500 MHz, MeOD)** δ 4.19 – 4.10 (m, 2H), 4.07 – 3.99 (m, 2H), 3.22 – 3.14 (m, 2H), 3.10 – 3.00 (m, 4H), 2.88 (dd, *J* = 12.8, 8.9 Hz, 2H), 1.78 – 1.62 (m, 4H); ^{13}C **NMR (126 MHz, MeOD)** δ 163.8 (q, $J_{C\text{-}F}$ = 33.4 Hz), 120.3 (q, $J_{C\text{-}F}$ = 291.3 Hz), 64.6, 63.7, 52.3, 44.4, 38.9; **HRMS** (m/z), calculated for $C_{10}H_{26}N_3O_4$: 252.1918, found $(M+H)^+$: 252.1912.

X-ray single-crystal structure determination

Intensity data for **4** and **9** were collected on a Bruker Apex-II CCD area detector diffractometer with graphite monochromated MoKα radiation (50 kV, 30 mA). The collection method involved *ω*- and *φ*-scans of width 0.5° and 1024x1024 bit data frames. Using *Olex2* [3], the crystal structures were solved by with the *ShelXT* [4] structure solution program using Intrinsic Phasing and refined with the *ShelXL* [5] refinement package using Least Squares minimization. Non-hydrogen atoms were first refined isotropically followed by anisotropic refinement by full matrix.

Crystal data for 4: $C_{34}H_{36}O_4S$ (*M* =540.69 g/mol): orthorhombic, space group $P2_12_12_1$ (no. 19), *a* = 9.2972(3) Å, *b* = 15.1161(5) Å, *c* = 20.4356(7) Å, *V* = 2871.97(17) Å^3, *Z* = 4, *T* = 173.15 K, μ(MoKα) = 0.150 mm^{-1}, *Dcalc* = 1.250 g/cm^3, 27581 reflections measured (3.352° ≤ 2Θ ≤

50.99°), 5356 unique (R_{int} = 0.0706, R_{sigma} = 0.0524) which were used in all calculations. The final R_1 was 0.0403 (I > 2σ(I)) and wR_2 was 0.0879 (all data). (CCDC 2076003).

Crystal data for 9: $C_{37}H_{44}O_4S_2$ (*M* =616.84 g/mol): monoclinic, space group $P2_1$ (no. 4), *a* = 14.4049(4) Å, *b* = 7.5907(2) Å, *c* = 16.9364(5) Å, *β* = 112.0590(10)°, *V* = 1716.32(8) $Å^3$, *Z* = 2, *T* = 173.15 K, μ(MoKα) = 0.192 mm^{-1}, *Dcalc* = 1.194 g/cm^3, 24077 reflections measured (2.594° ≤ 2Θ ≤ 56.816°), 8570 unique (R_{int} = 0.0519, R_{sigma} = 0.0615) which were used in all calculations. The final R_1 was 0.0473 (I > 2σ(I)) and wR_2 was 0.1200 (all data). (CCDC 2076004).

References

1. Bode, M. L.; Gates, P. J.; Gebretnsae, S. Y.; Vleggaar, R. *Tetrahedron,* **2010**, 66, 2026-2036.
2. White, J. D.; Lincoln, C. M.; Yang, J.; Martin, W. H. C.; Chan, D. B. *J. Org. Chem.*, **2008**, *73*, 4139-4150.
3. Dolomanov, O. V.; Bourhis, L. J.; Gildea, R.J.; Howard, J. A. K.; Puschmann, H. J. Appl. Cryst., 2009, 42, 339-341.
4. Sheldrick, G. M. *Acta Cryst*., **2015**, *A71*, 3-8.
5. Sheldrick, G. M. *Acta Cryst*., **2015**, *C71*, 3-8.

^{1}H NMR Spectrum of compound **4** (500 MHz, $CDCl_3$)

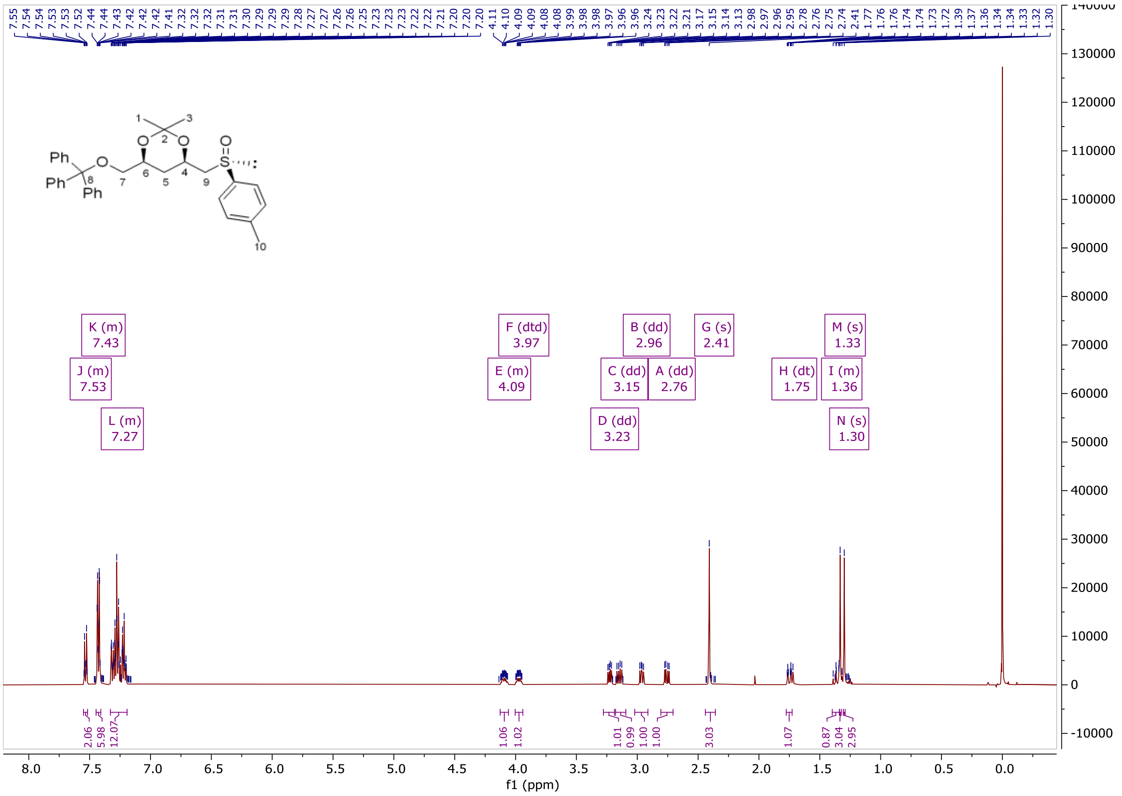

^{13}C NMR Spectrum of compound **4** (126 MHz, $CDCl_3$)

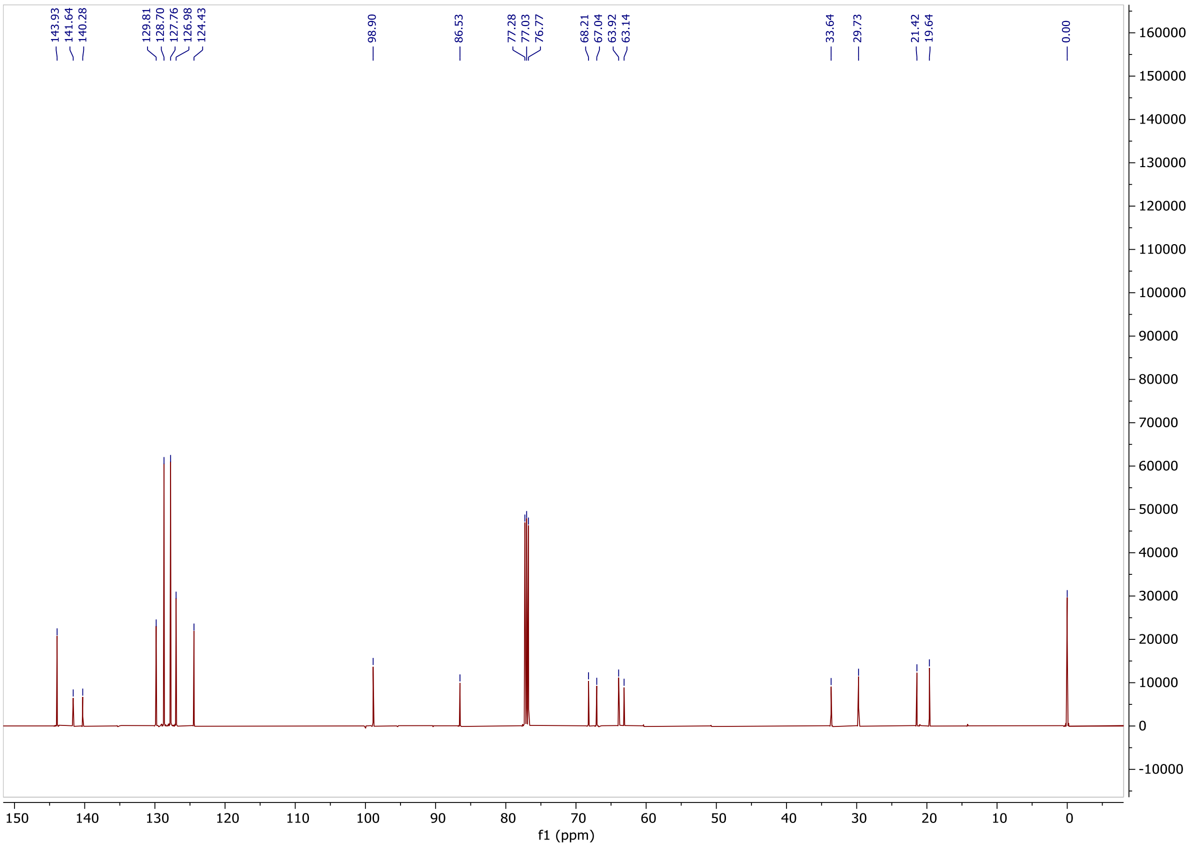

DEPT-135 Spectrum of compound **4**

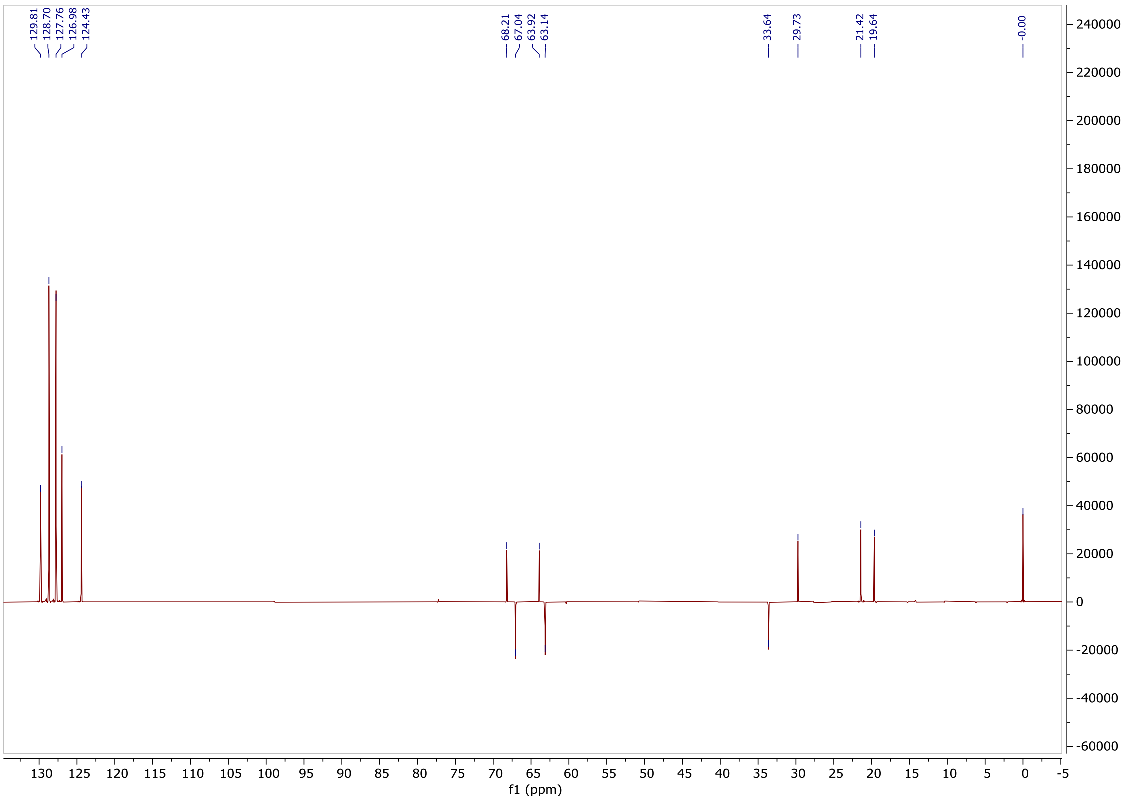

COSY Spectrum of compound **4**

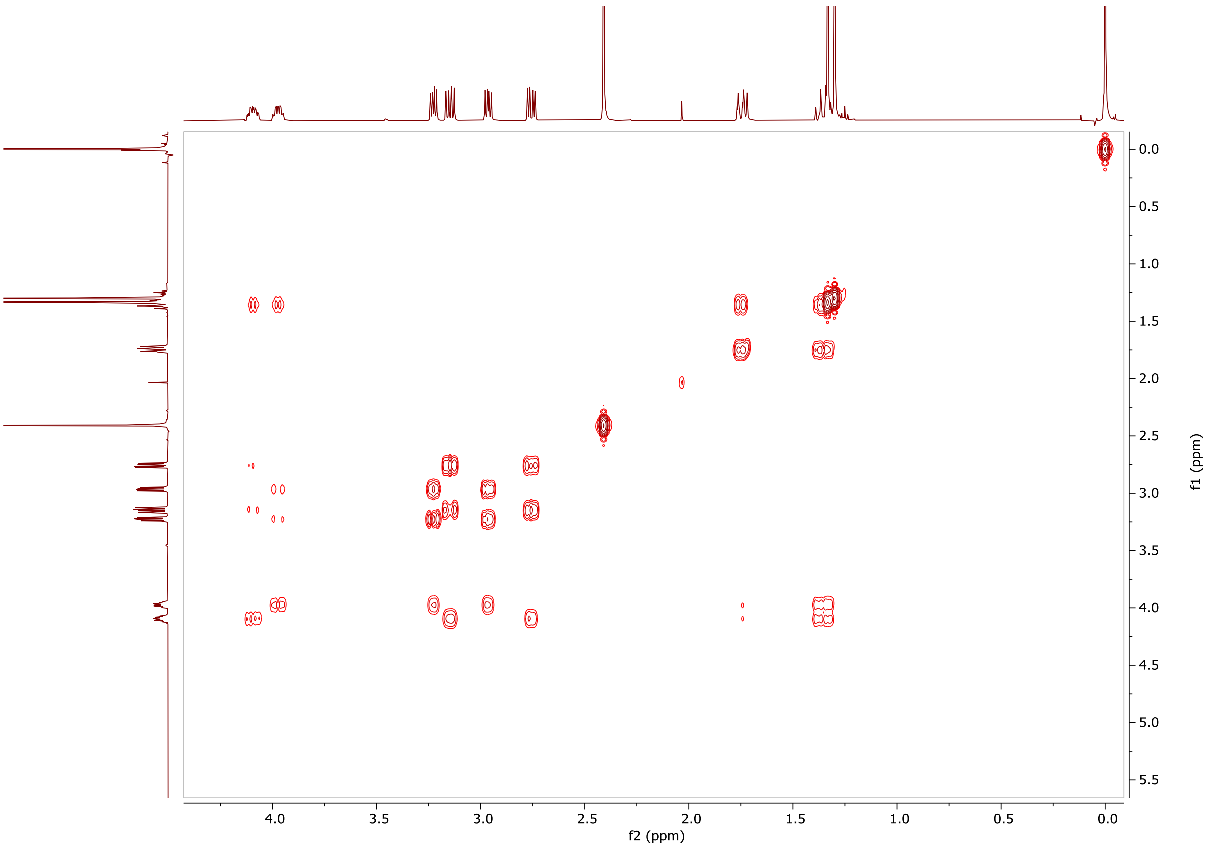

HSQC Spectrum of compound **4**

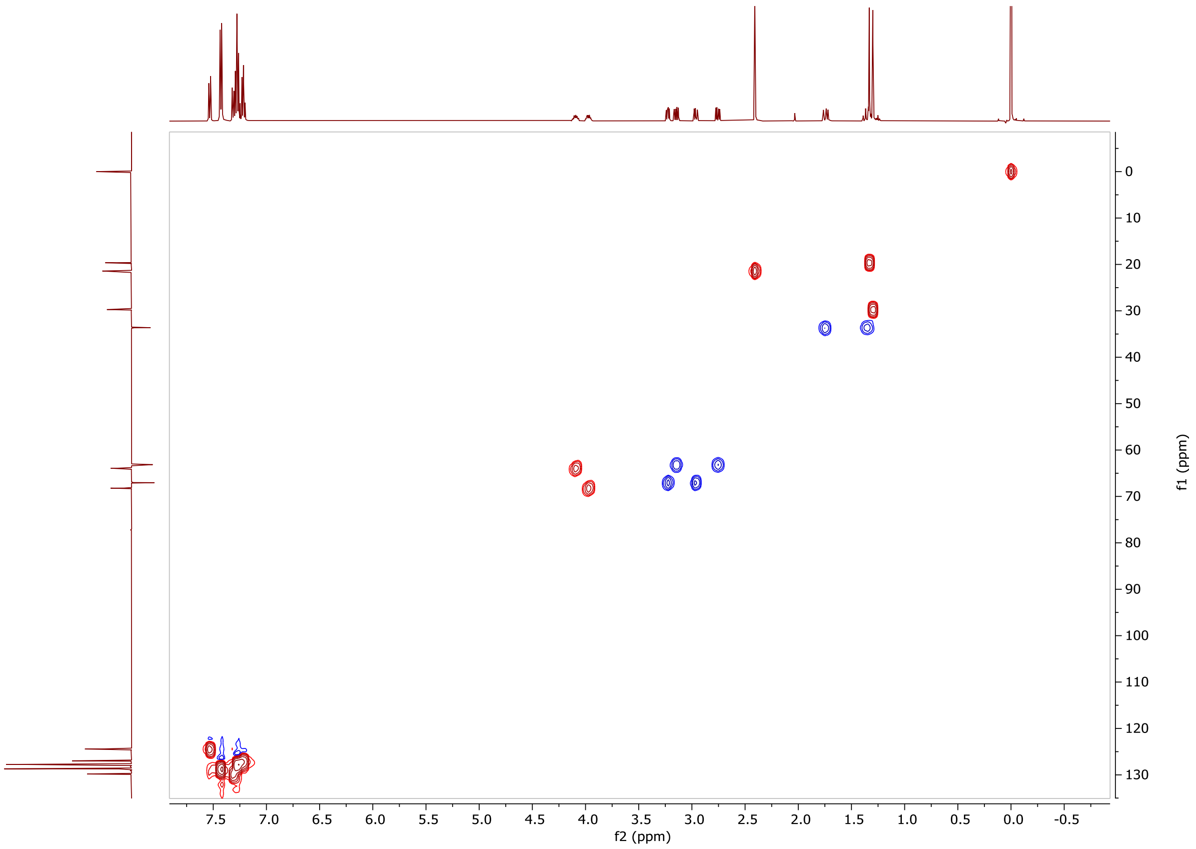

^{1}H NMR Spectrum of compound **5** (500 MHz, $CDCl_3$)

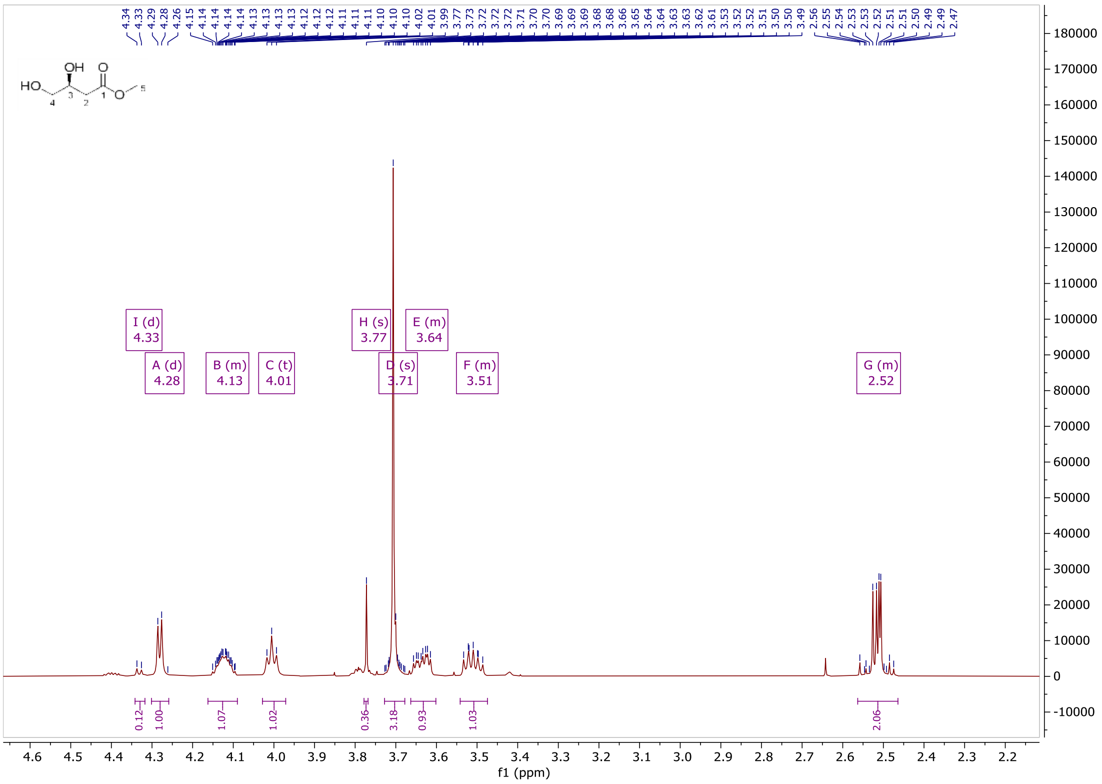

^{13}C NMR Spectrum of compound **5** (126 MHz, $CDCl_3$)

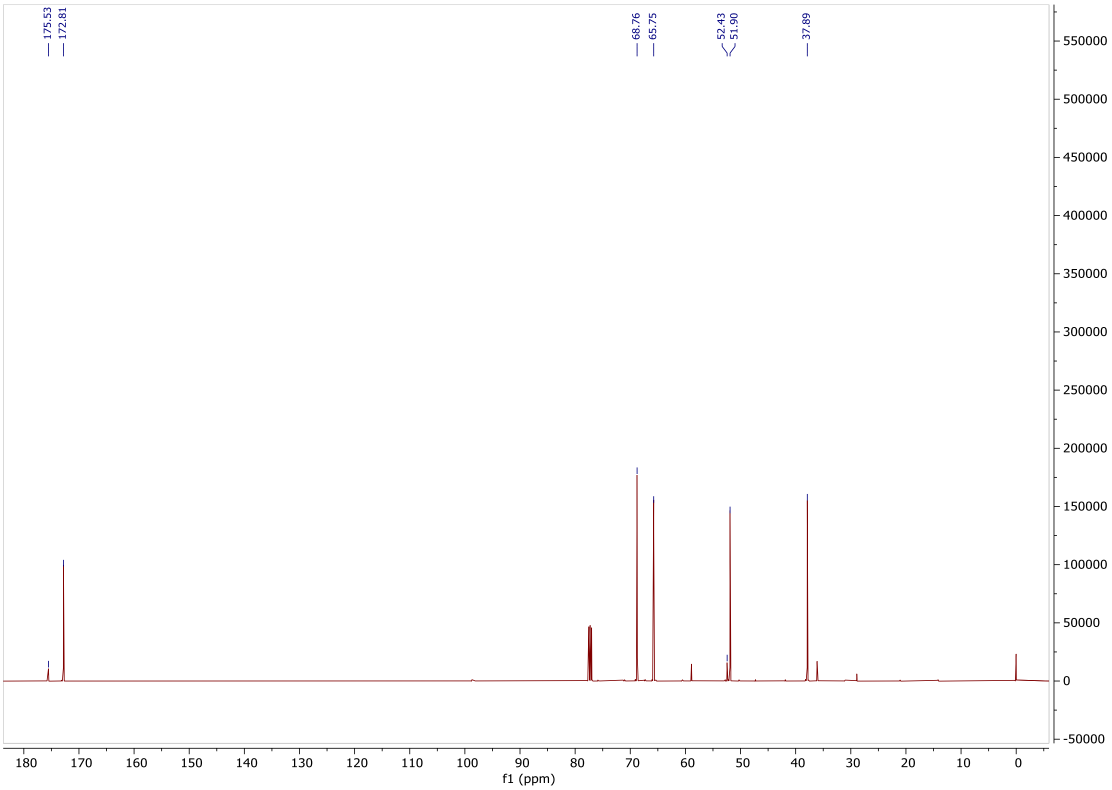

DEPT-135 Spectrum of compound **5**

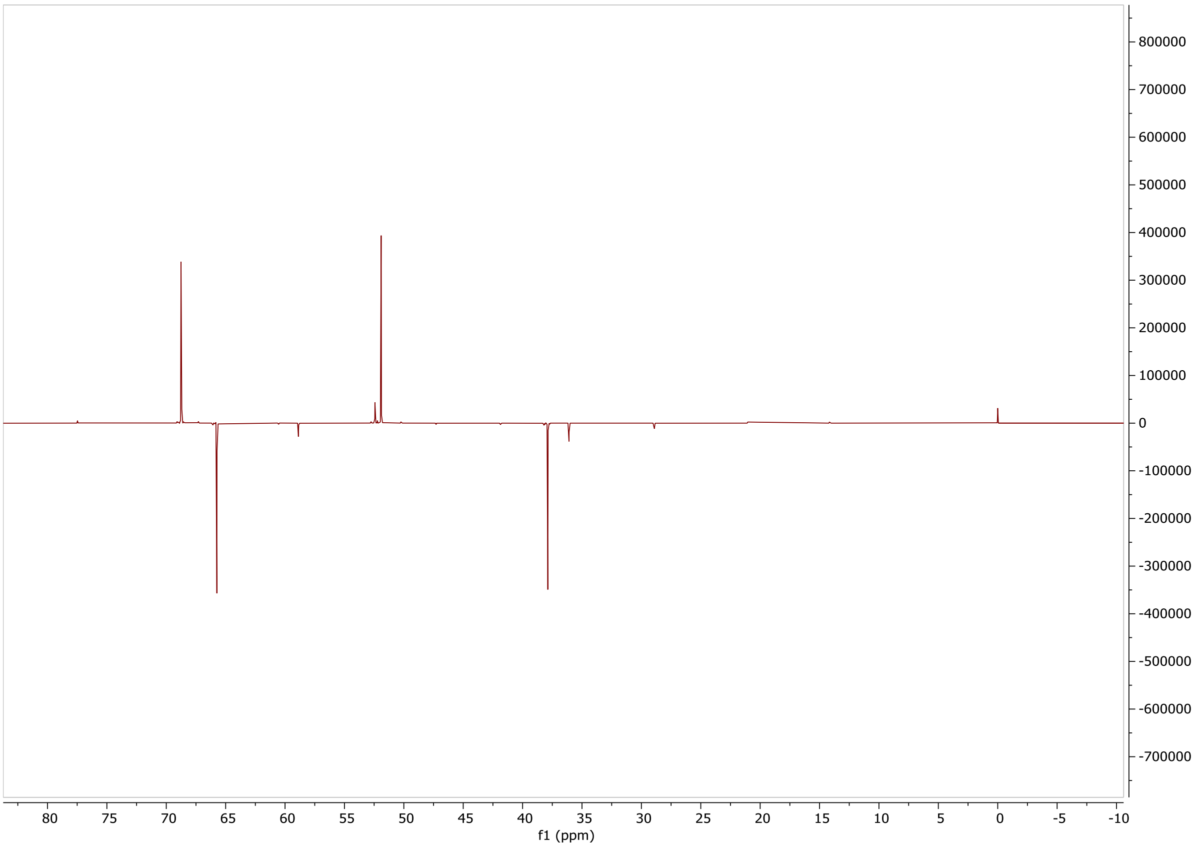

COSY Spectrum of compound **5**

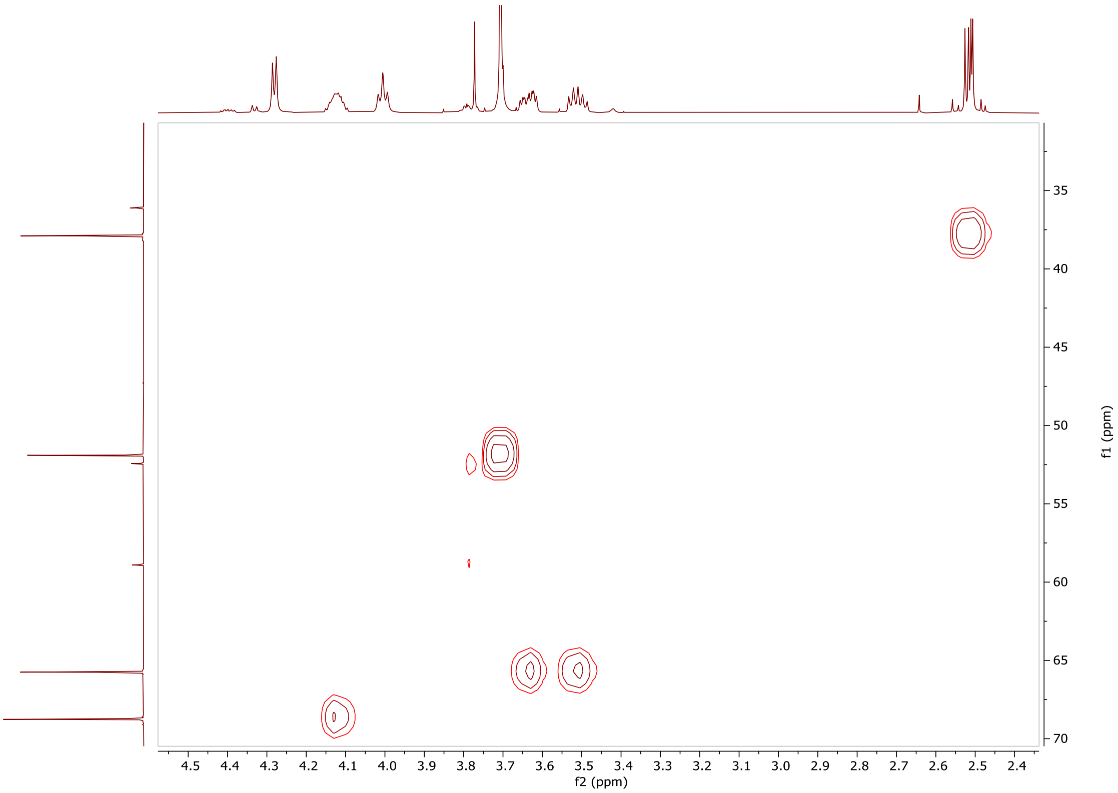

^{1}H NMR Spectrum of compound **6** (300 MHz, $CDCl_3$)

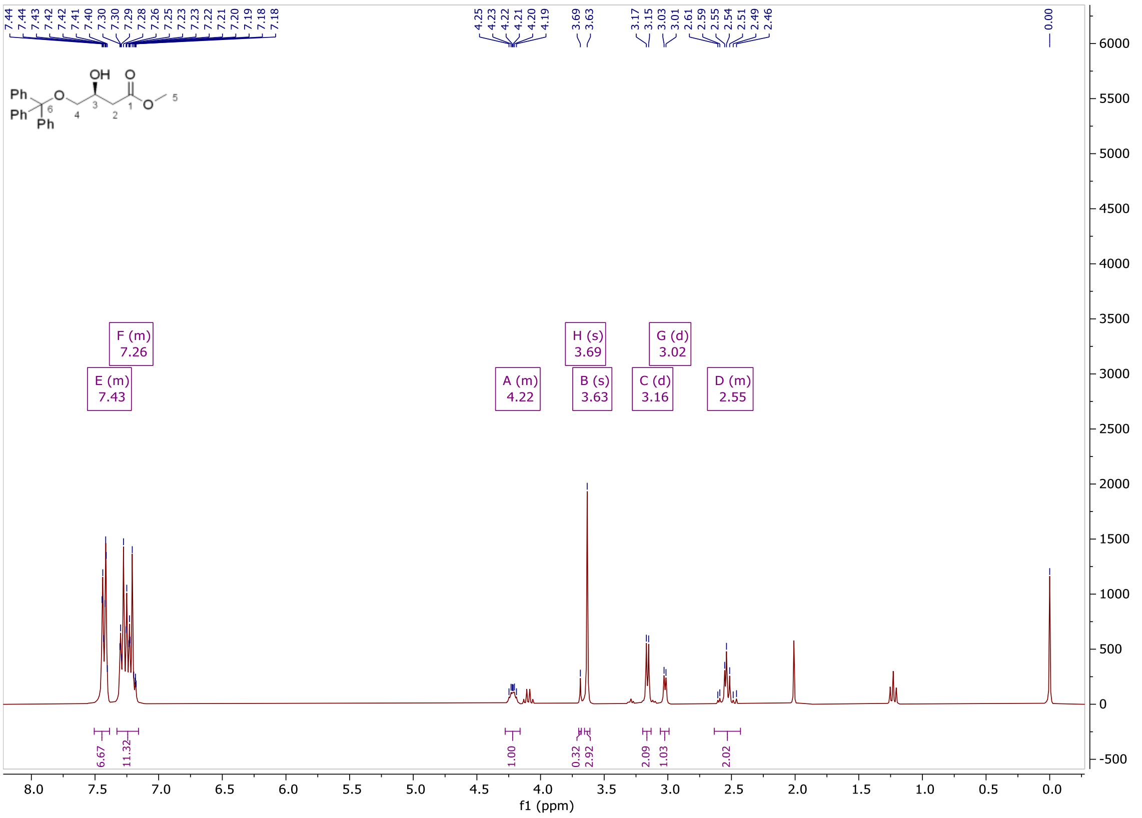

^{13}C NMR Spectrum of compound **6** (75 MHz, $CDCl_3$)

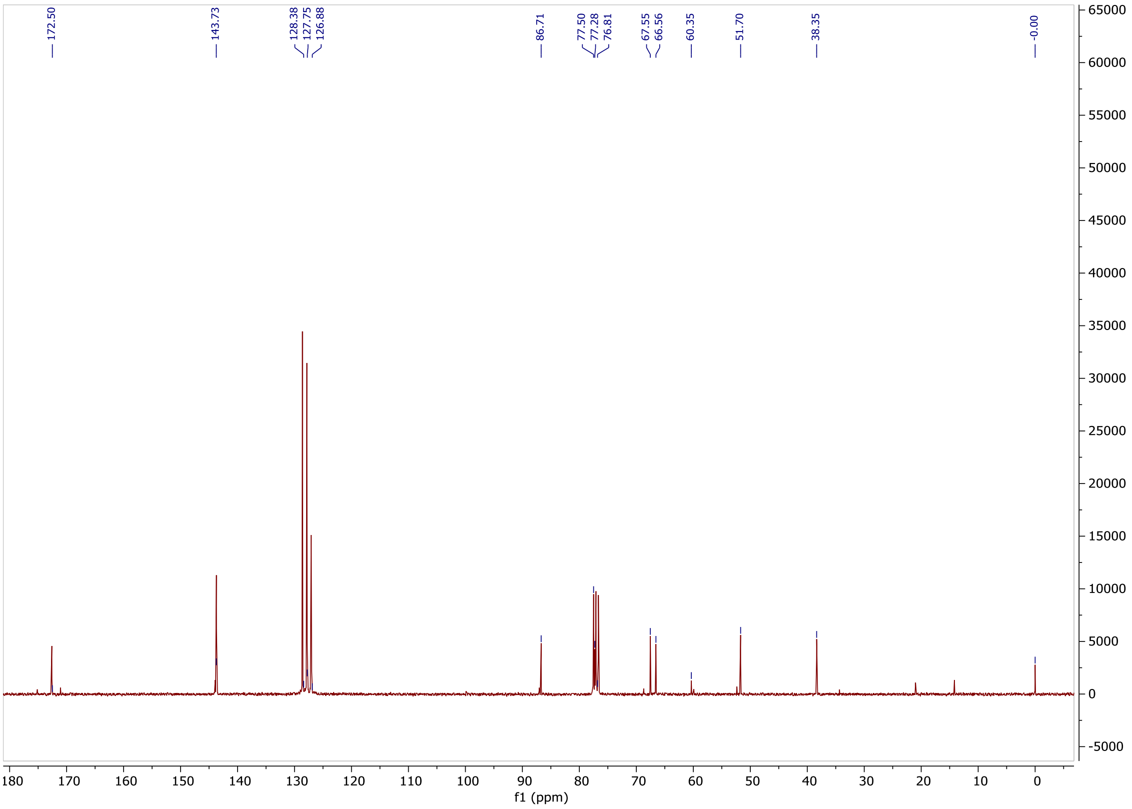

^{1}H NMR Spectrum of compound **7** (300 MHz, $CDCl_3$)

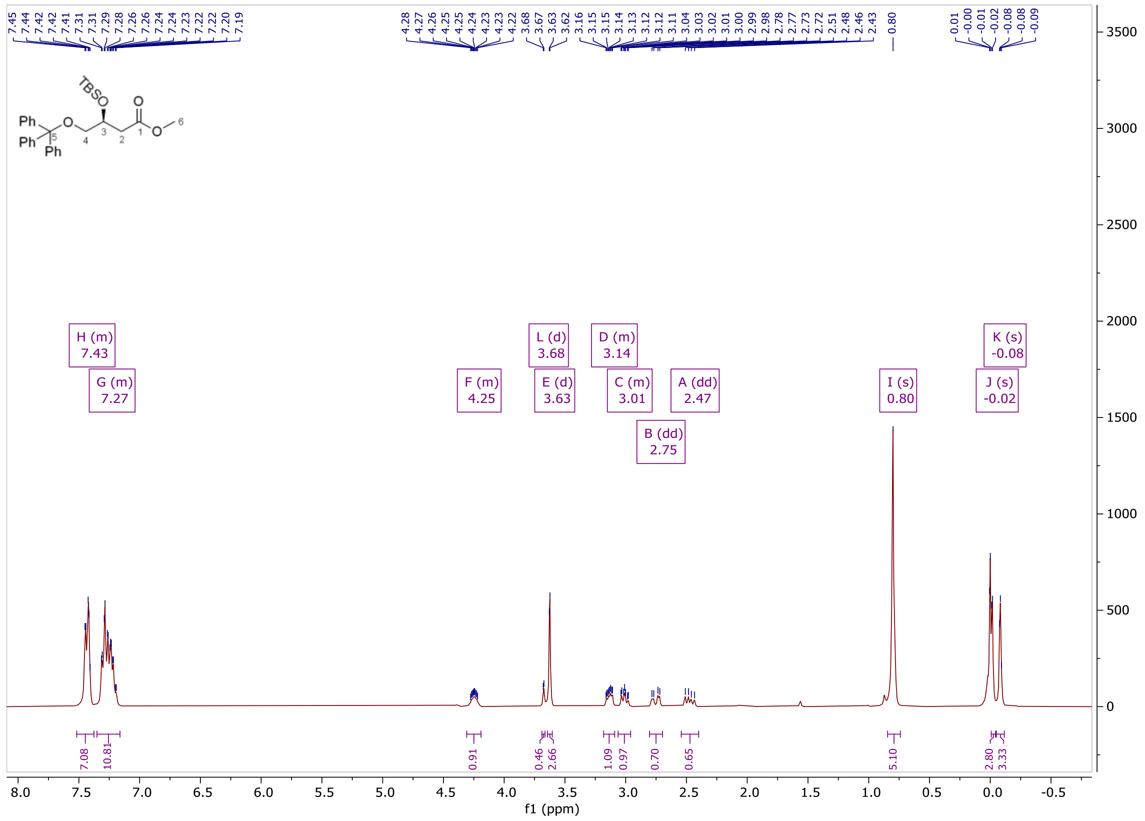

^{13}C NMR Spectrum of compound **7** (75 MHz, $CDCl_3$)

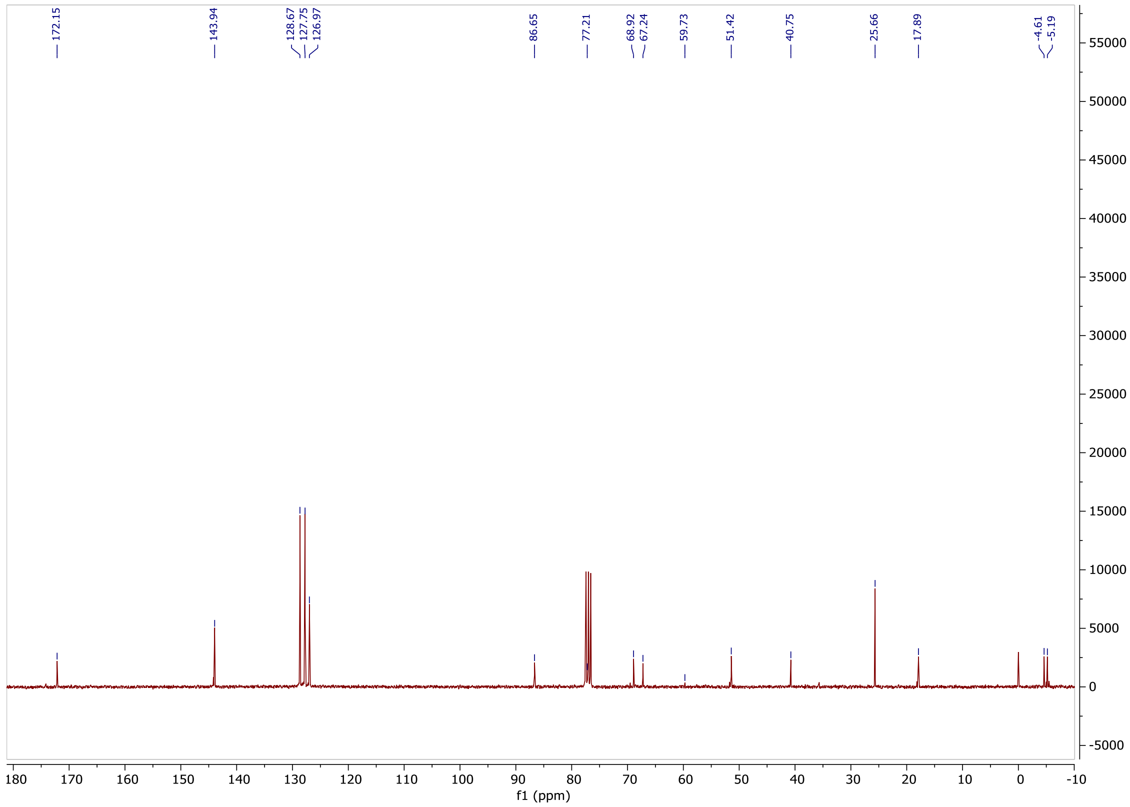

COSY Spectrum of compound **7**

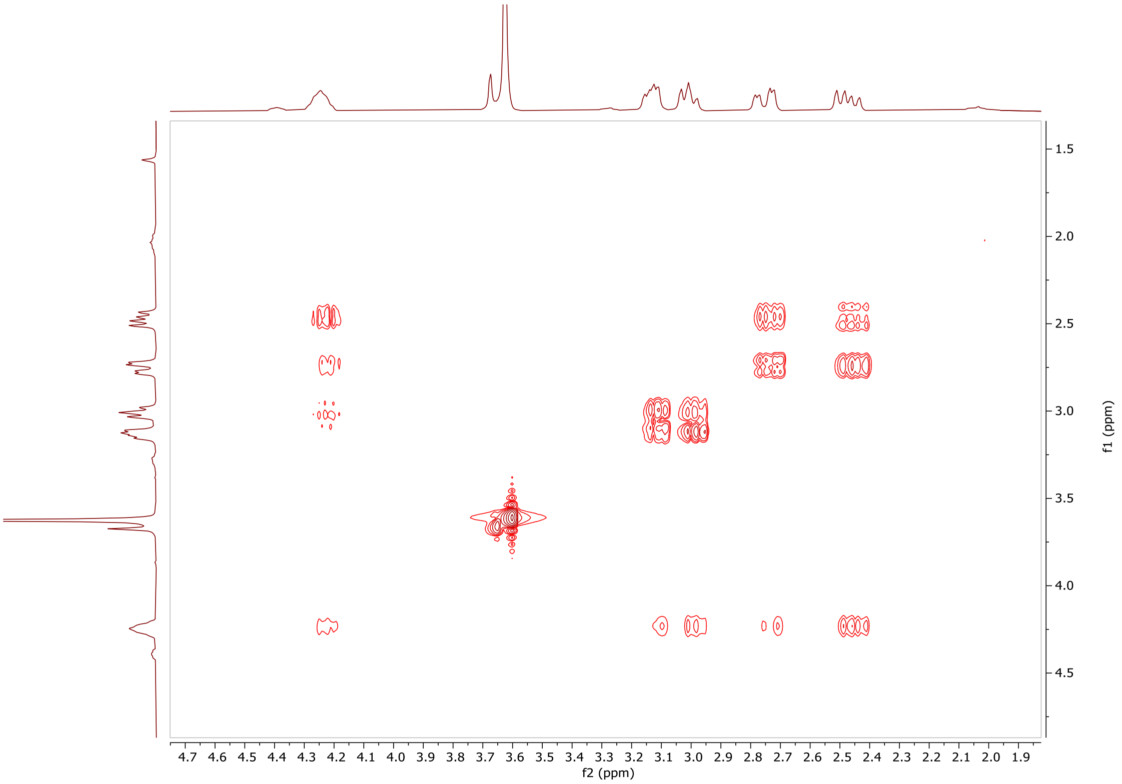

^{1}H NMR Spectrum of compound **8** (300 MHz, $CDCl_3$)

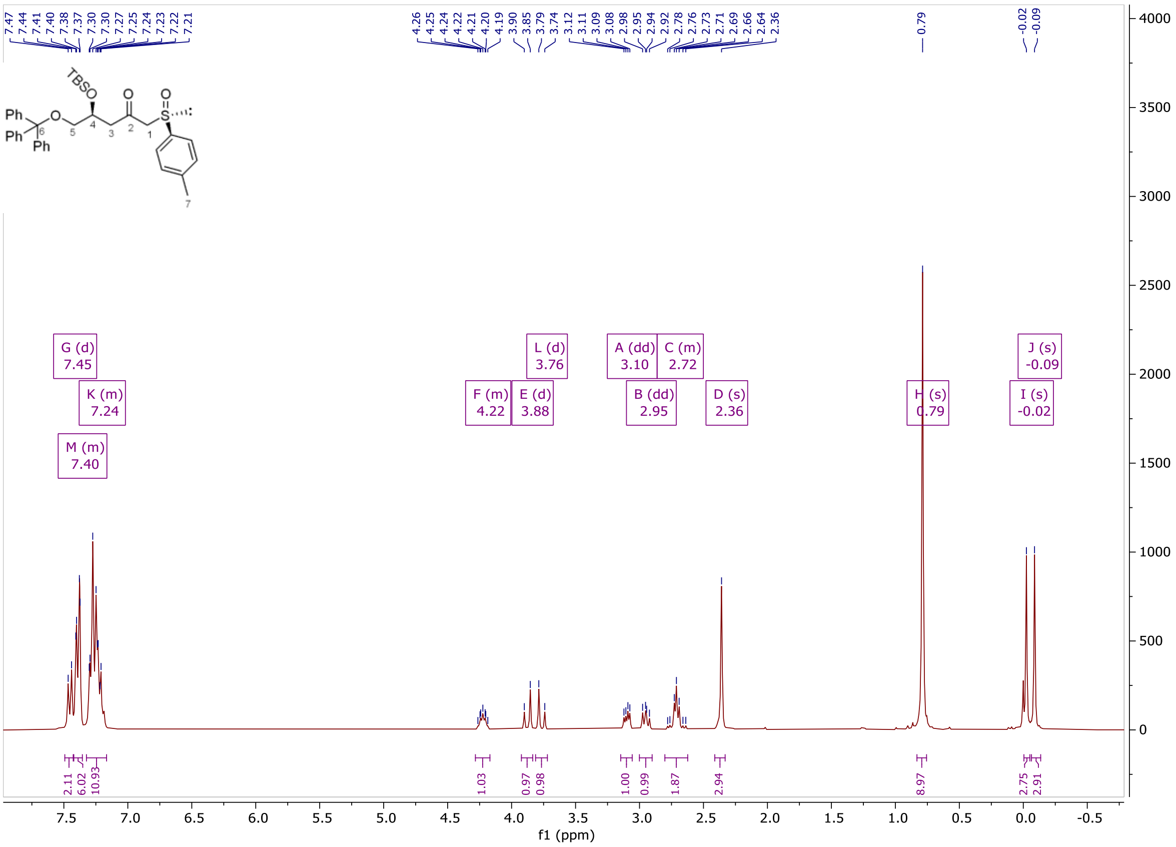

^{13}C NMR Spectrum of compound **8** (75 MHz, $CDCl_3$)

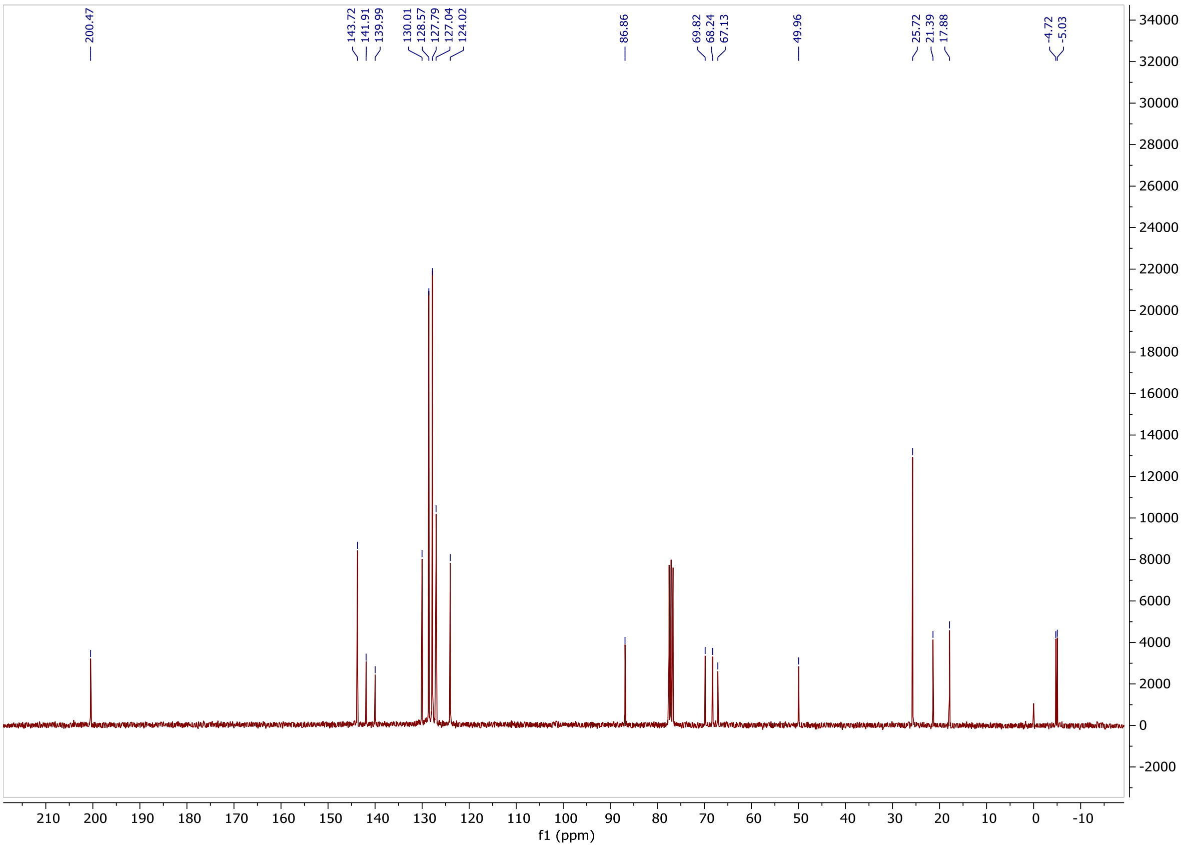

COSY Spectrum of compound **8**

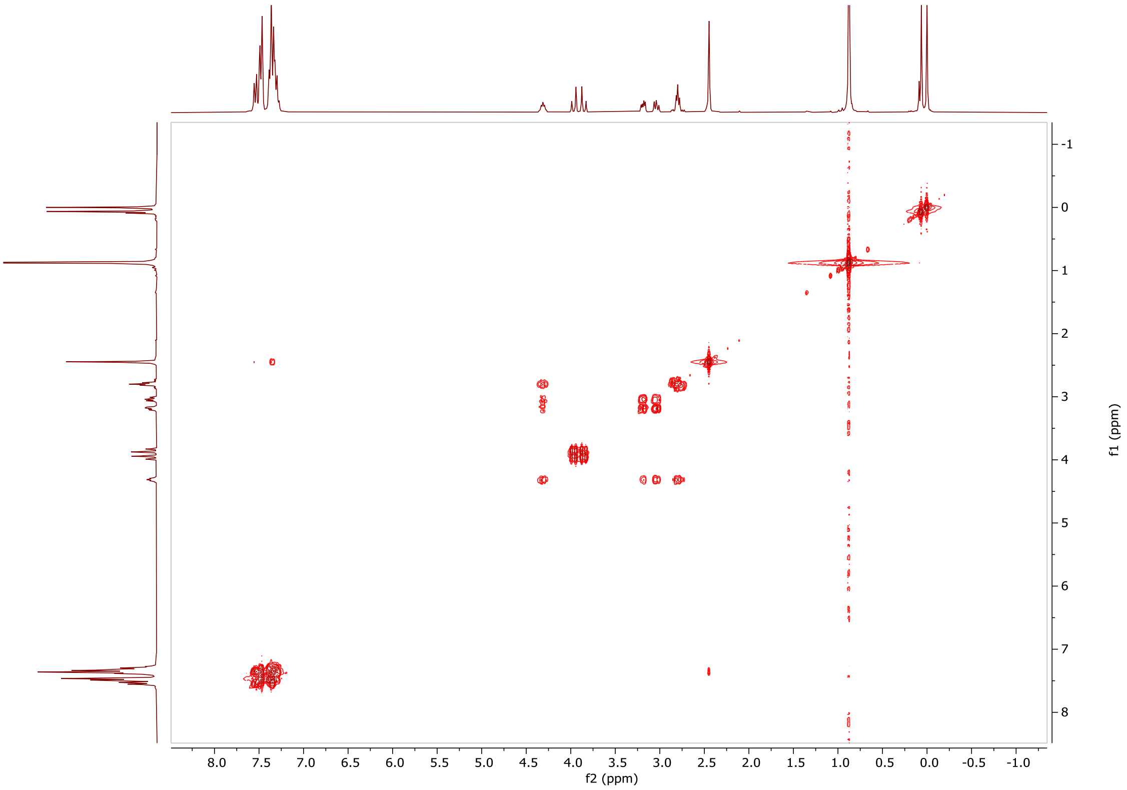

HSQC Spectrum of compound **8**

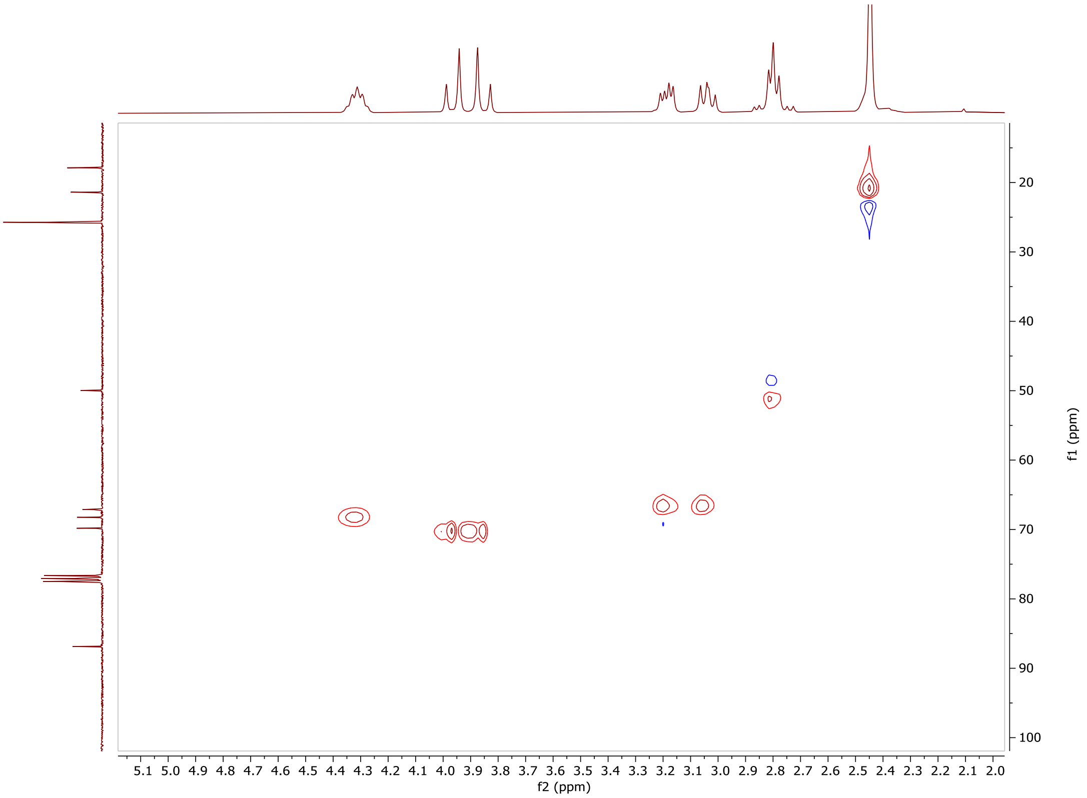

^{1}H NMR Spectrum of compound **9** (300 MHz, $CDCl_3$)

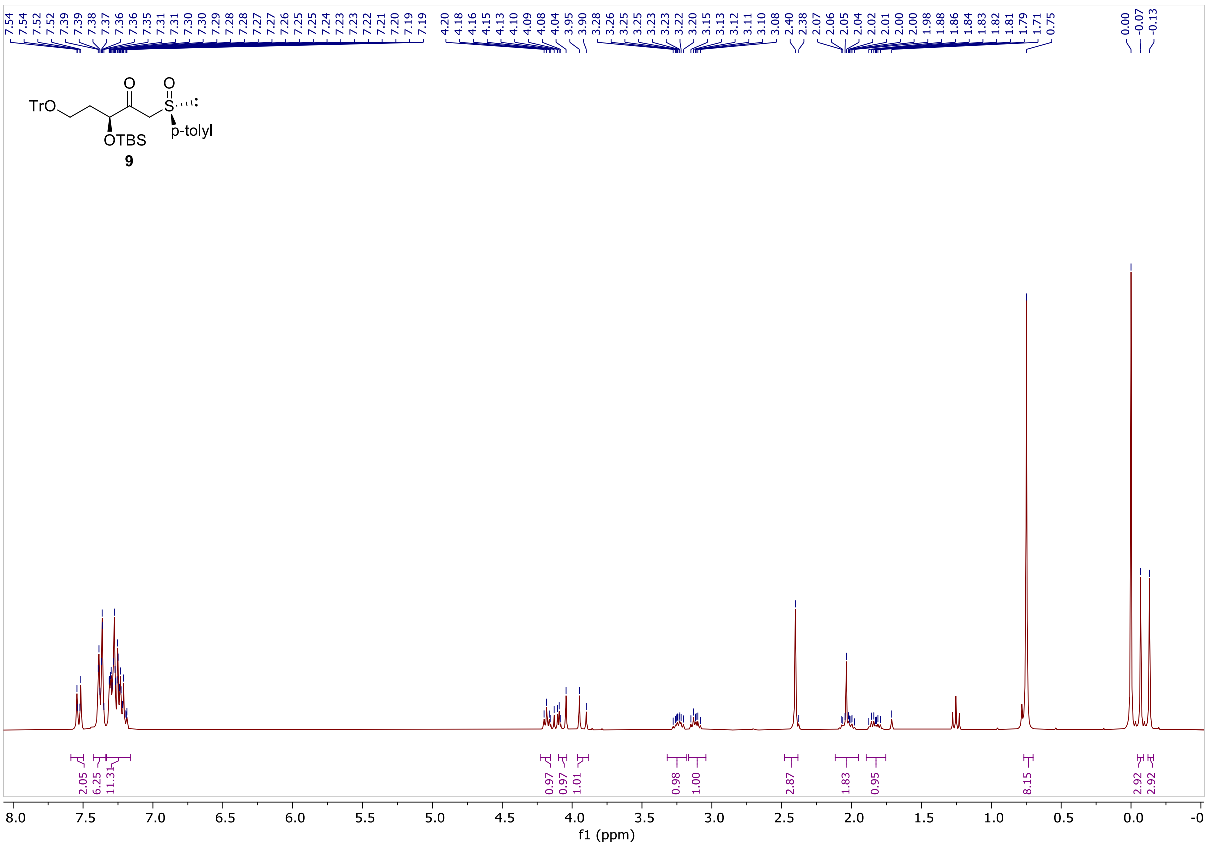

^{13}C NMR Spectrum of compound **9** (75 MHz, $CDCl_3$)

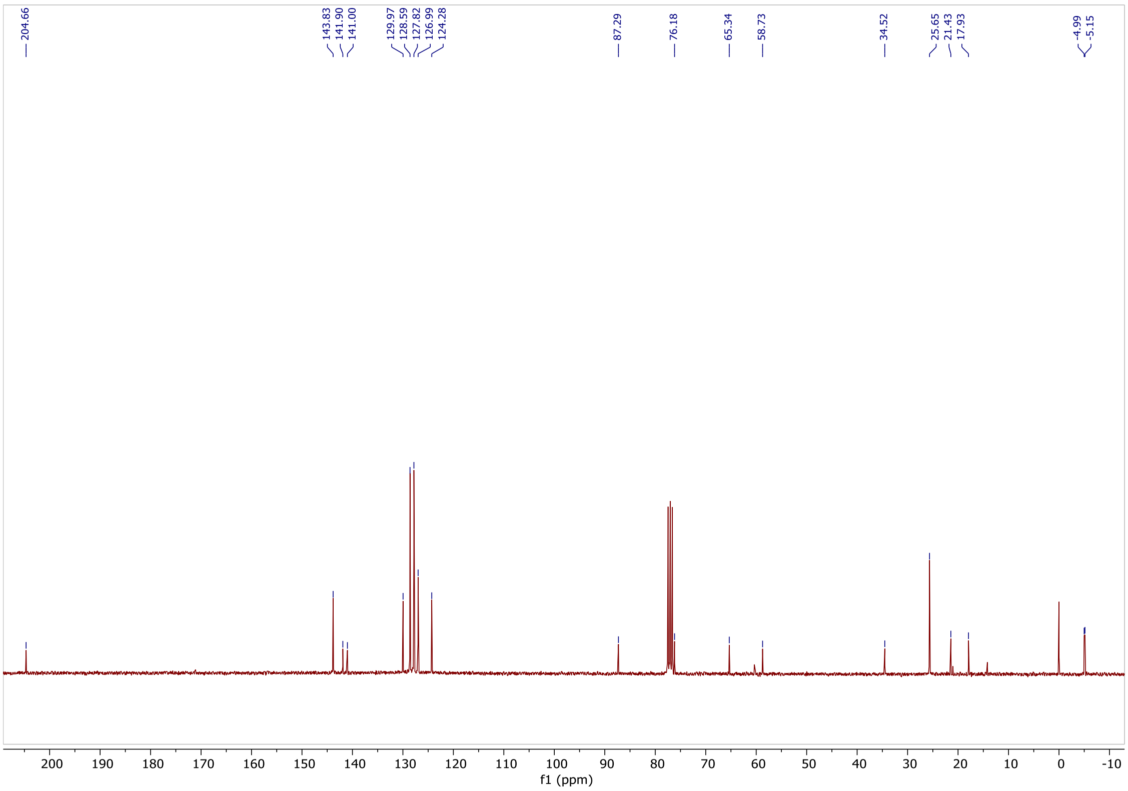

COSY Spectrum of compound **9**

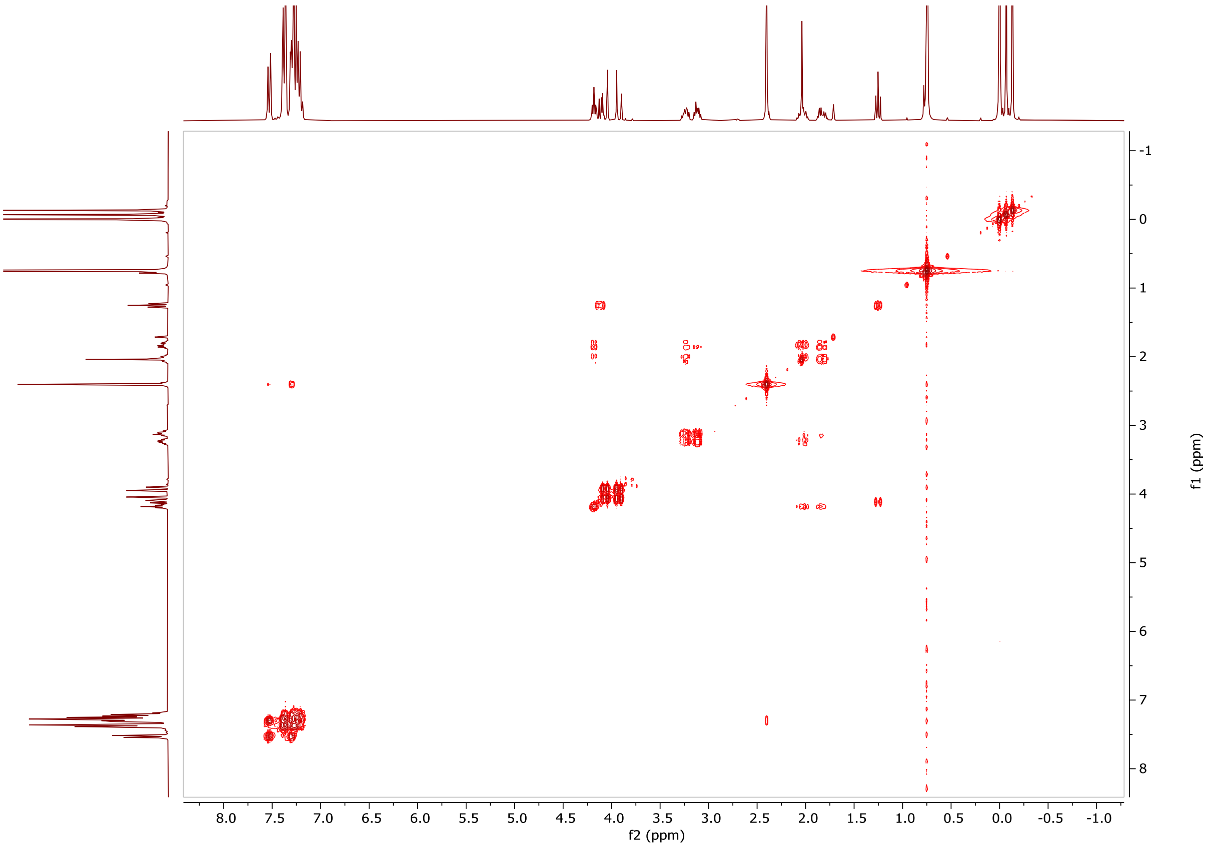

HSQC Spectrum of compound **9**

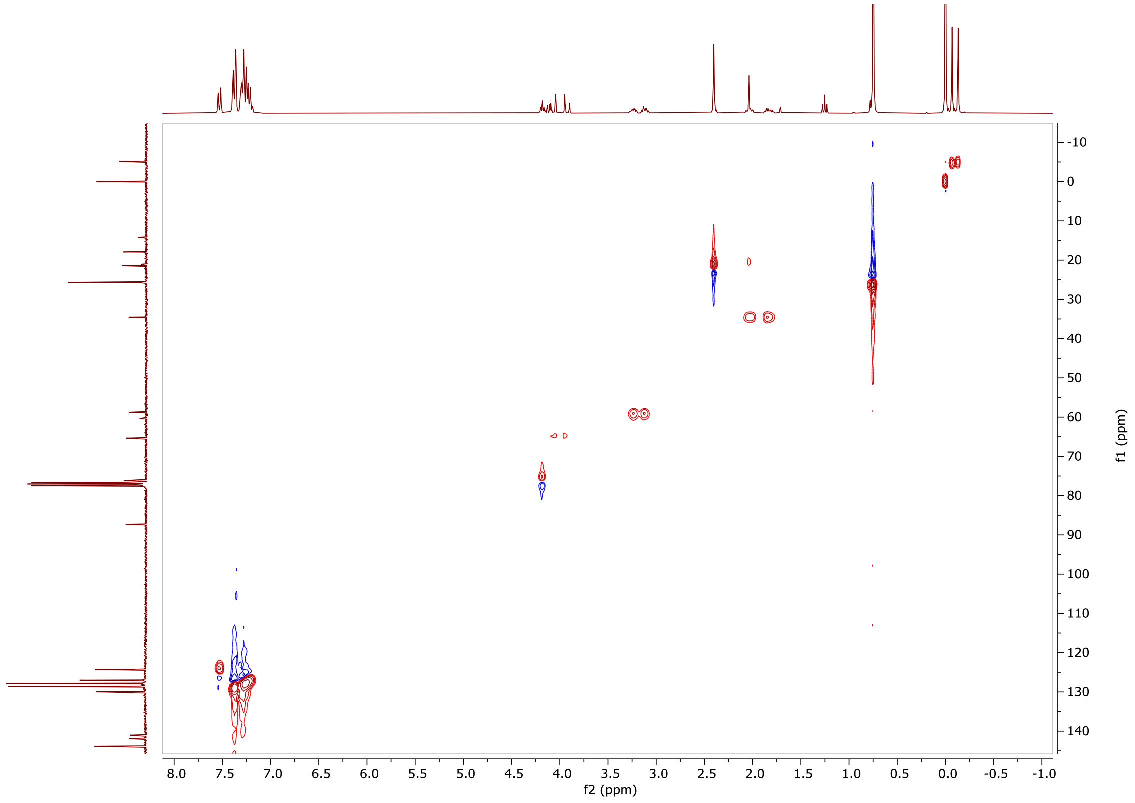

^{1}H NMR Spectrum of compound **16** (500 MHz, $CDCl_3$)

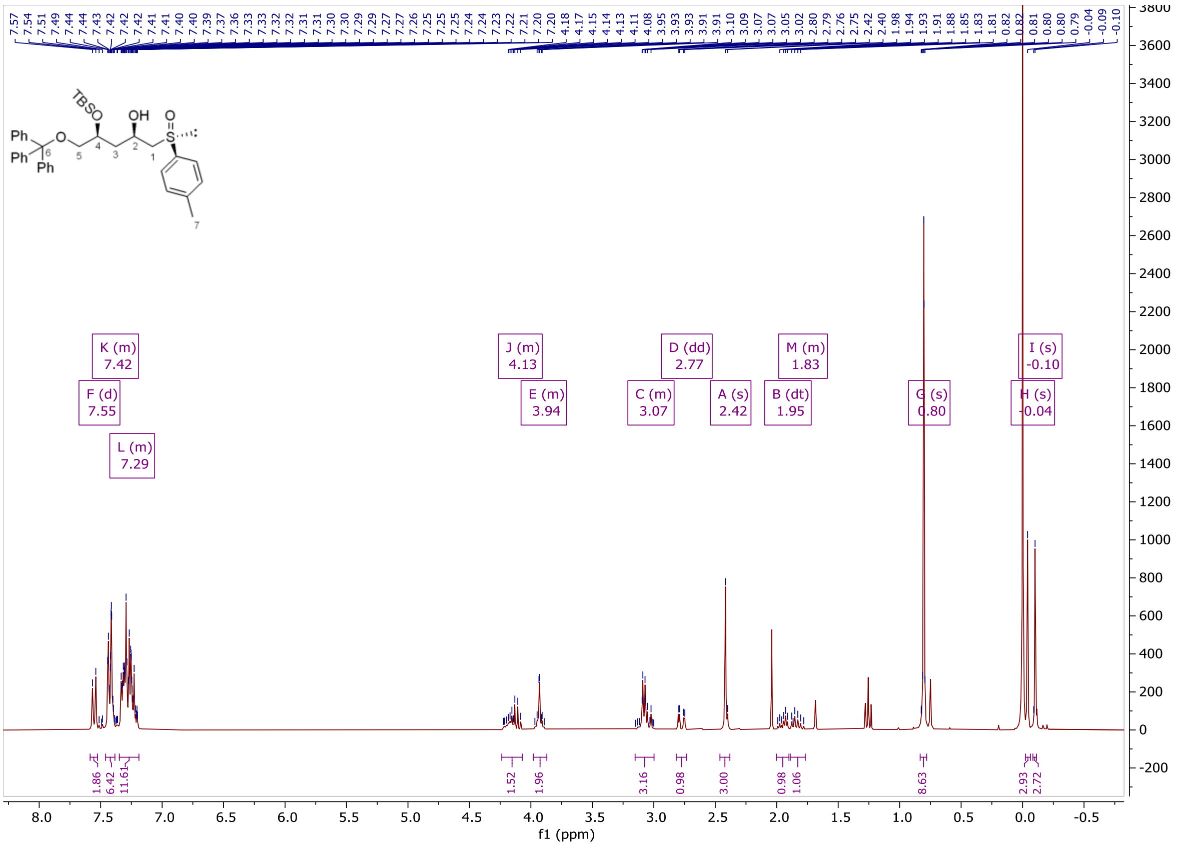

^{13}C NMR Spectrum of compound **16** (126 MHz, $CDCl_3$)

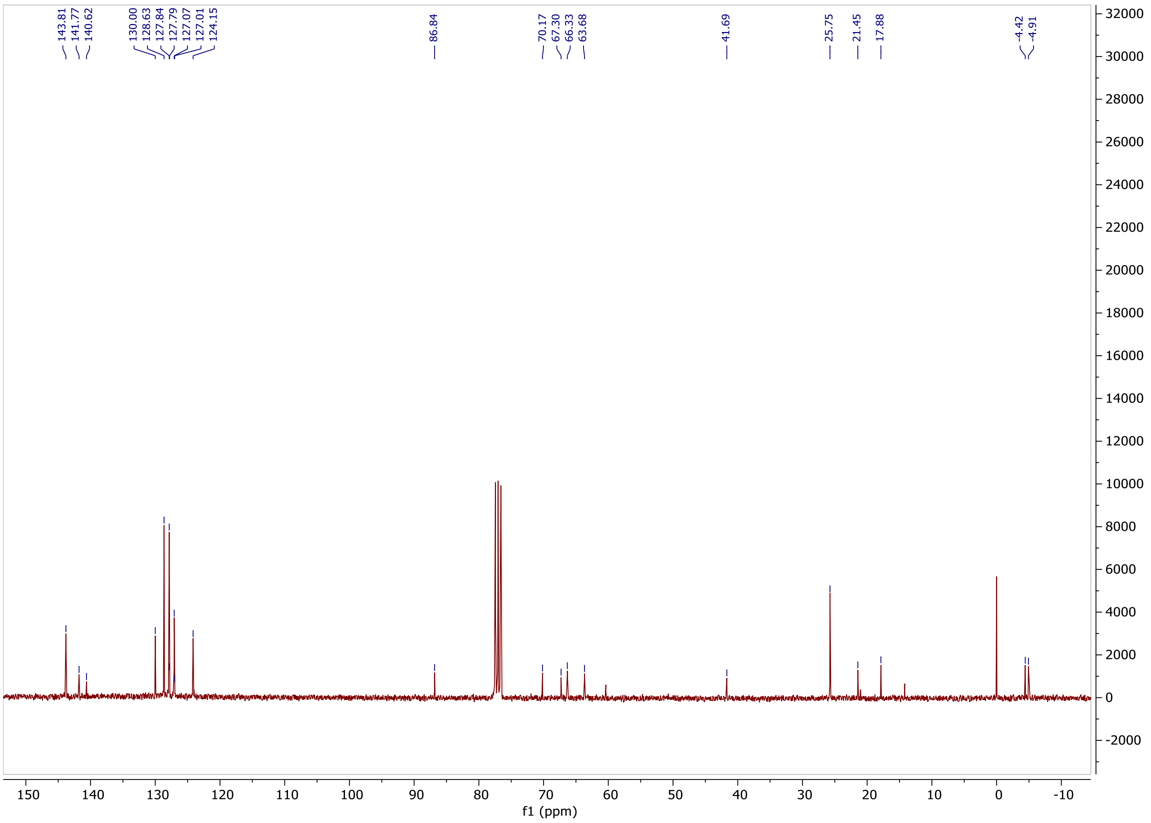

DEPT-135 Spectrum of compound **16**

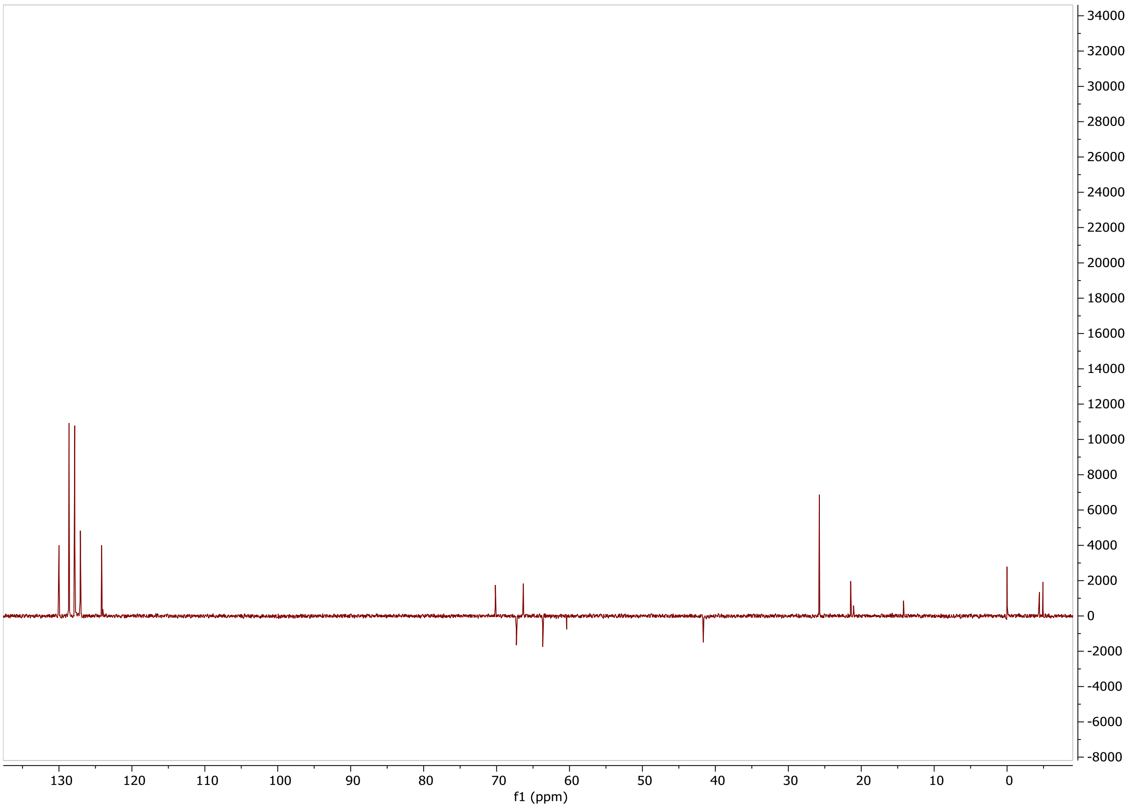

COSY Spectrum of compound **16**

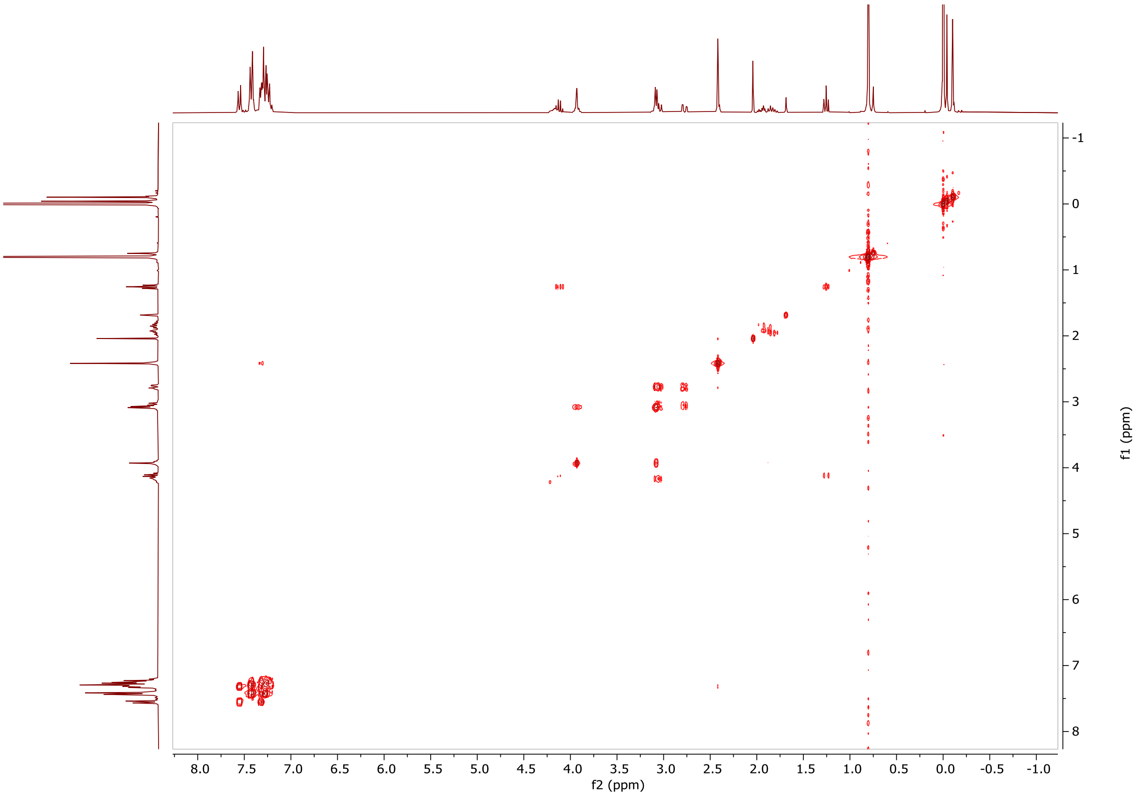

^{1}H NMR Spectrum of compound **17** (500 MHz, $CDCl_3$)

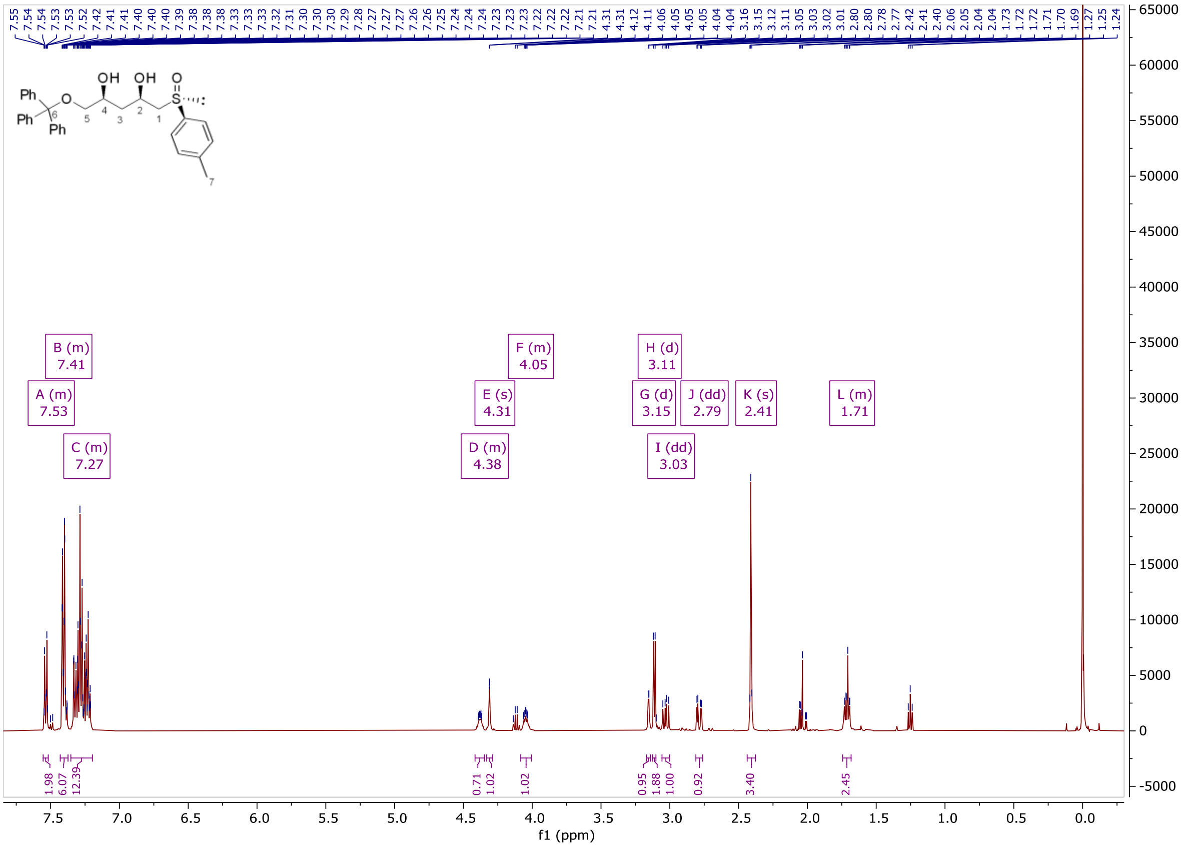

^{13}C NMR Spectrum of compound **17** (126 MHz, $CDCl_3$)

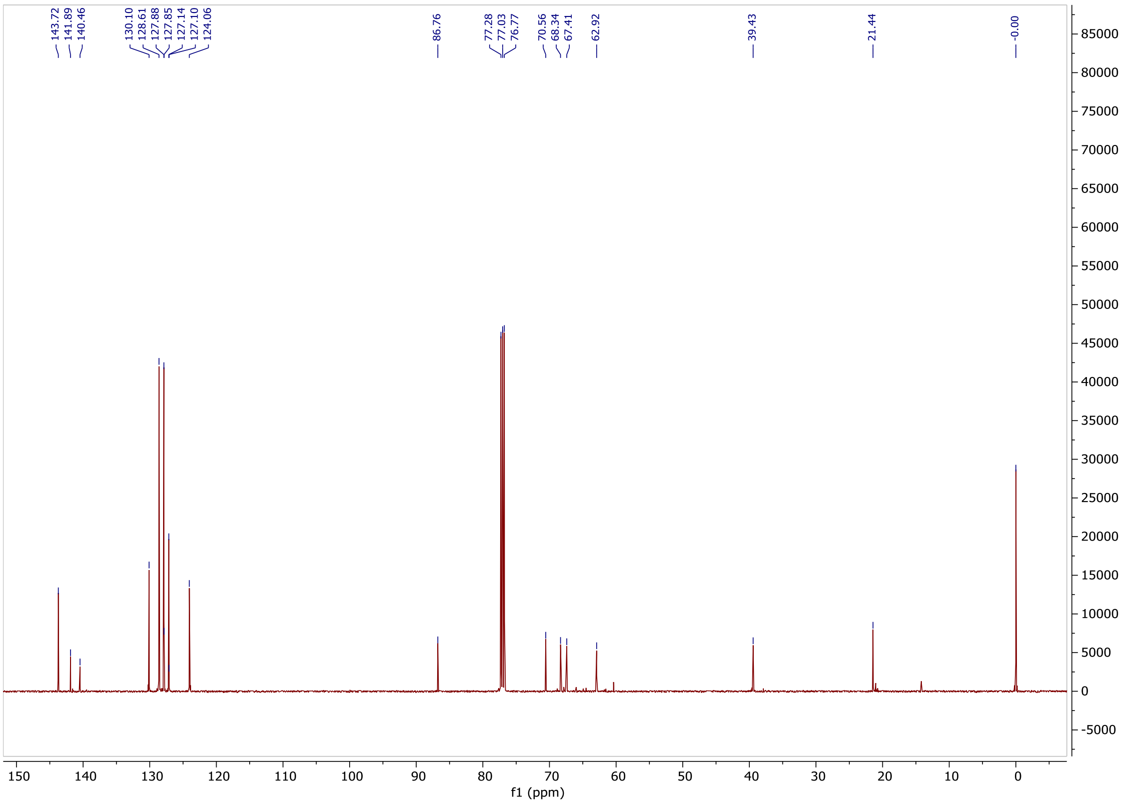

DEPT-135 Spectrum of compound **17**

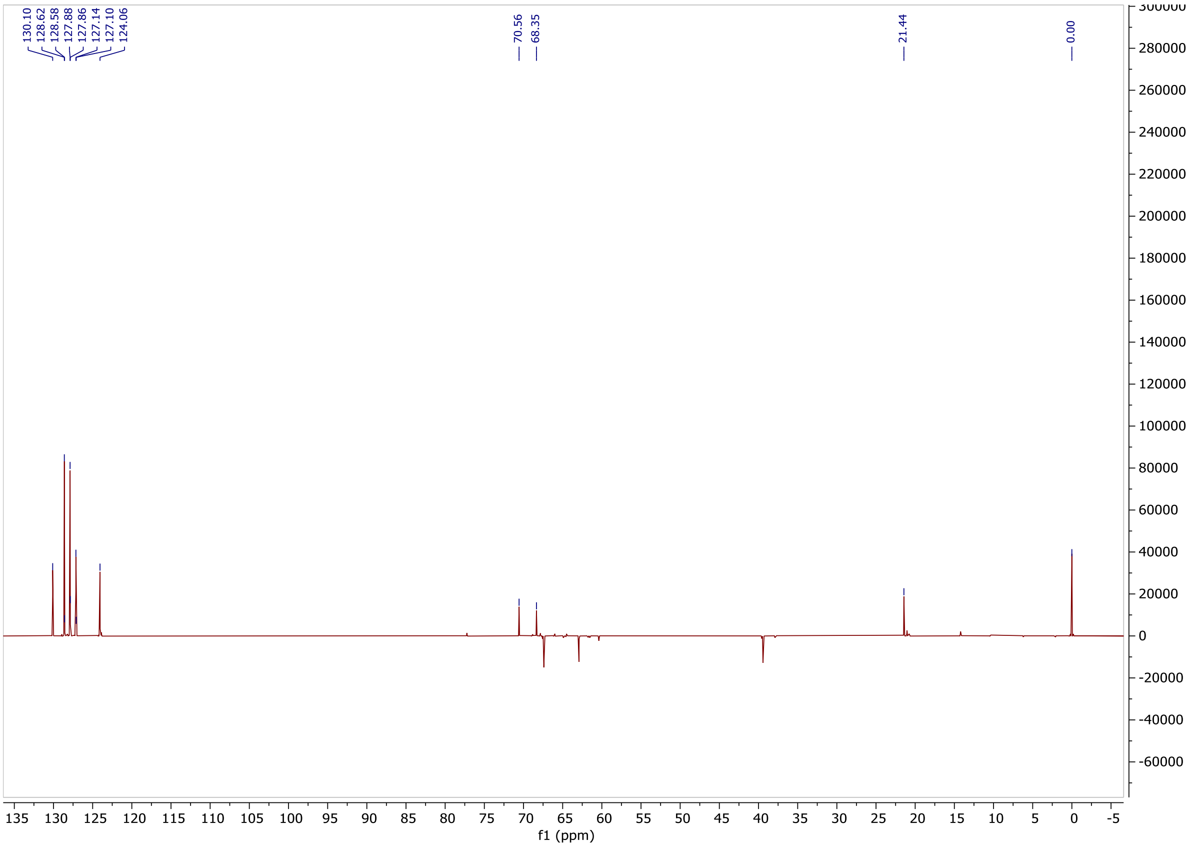

COSY Spectrum of compound **17**

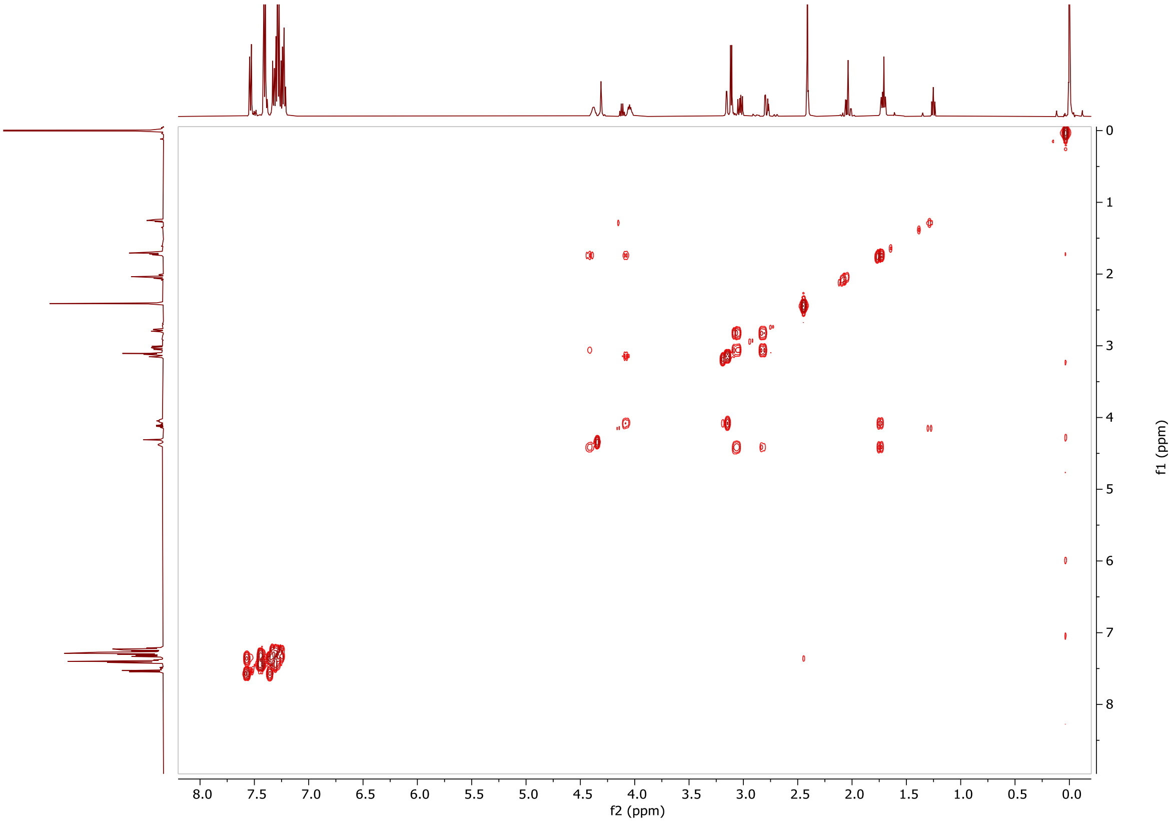

HSQC Spectrum of compound **17**

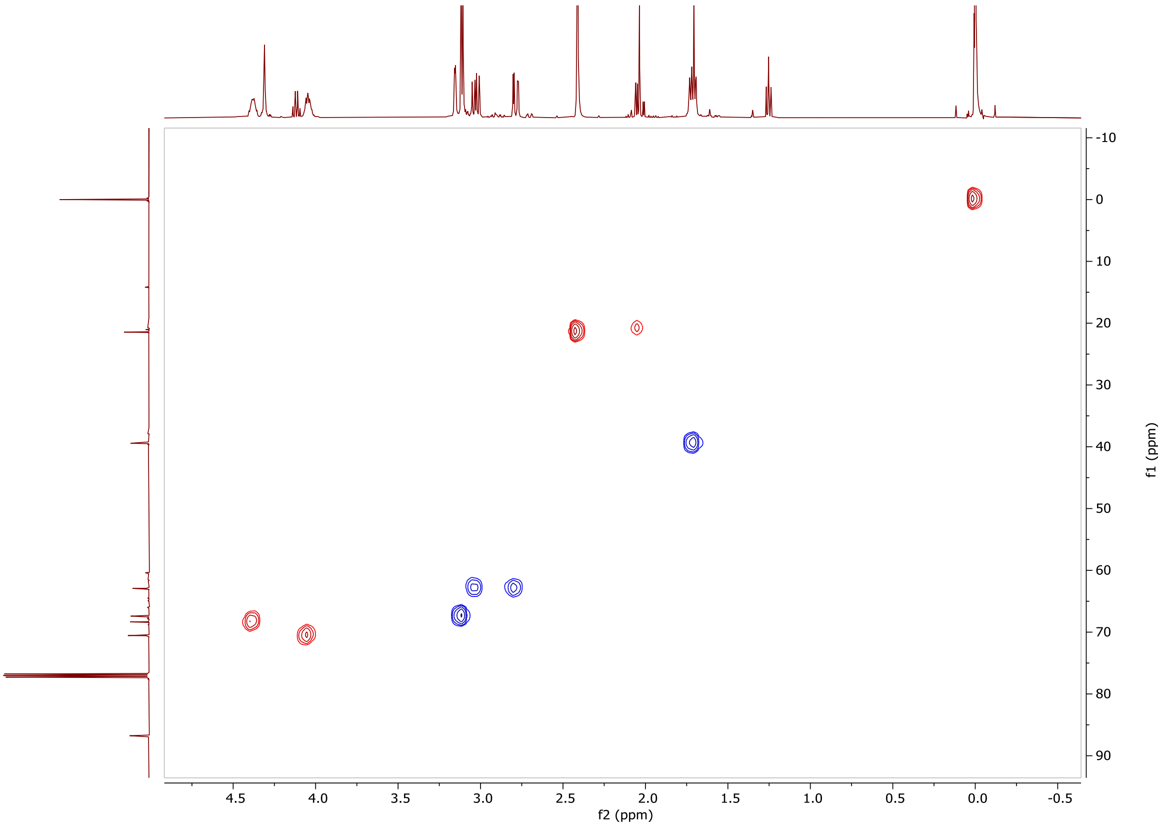

^{1}H NMR Spectrum of compound **18** (300 MHz, $CDCl_3$)

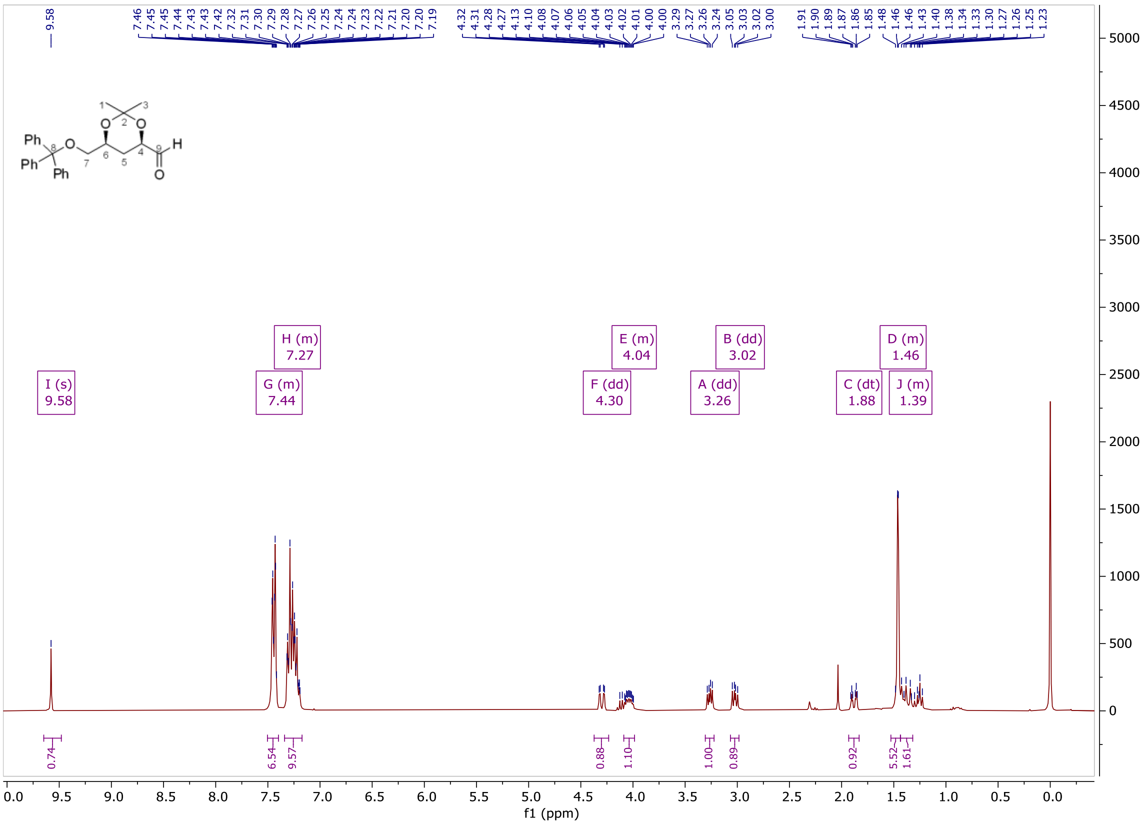

^{13}C NMR Spectrum of compound **18** (75 MHz, $CDCl_3$)

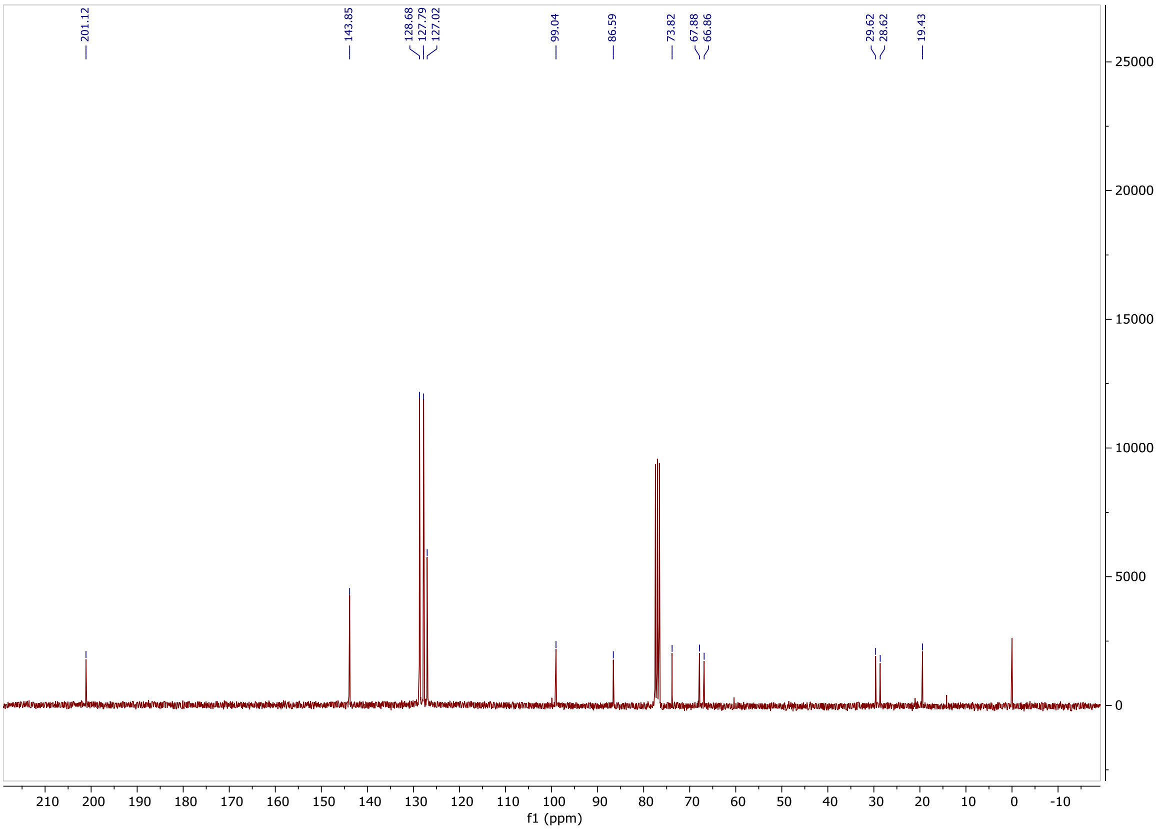

COSY Spectrum of compound **18**

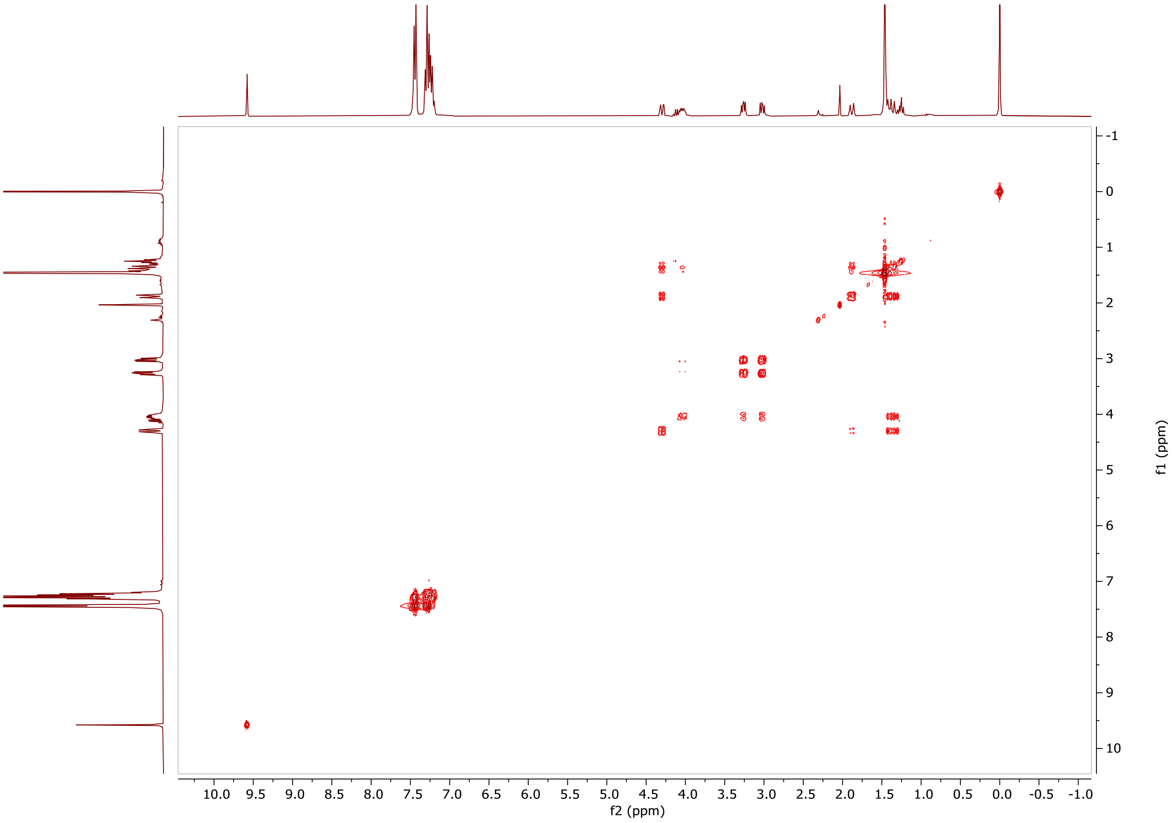

HSQC Spectrum of compound **18**

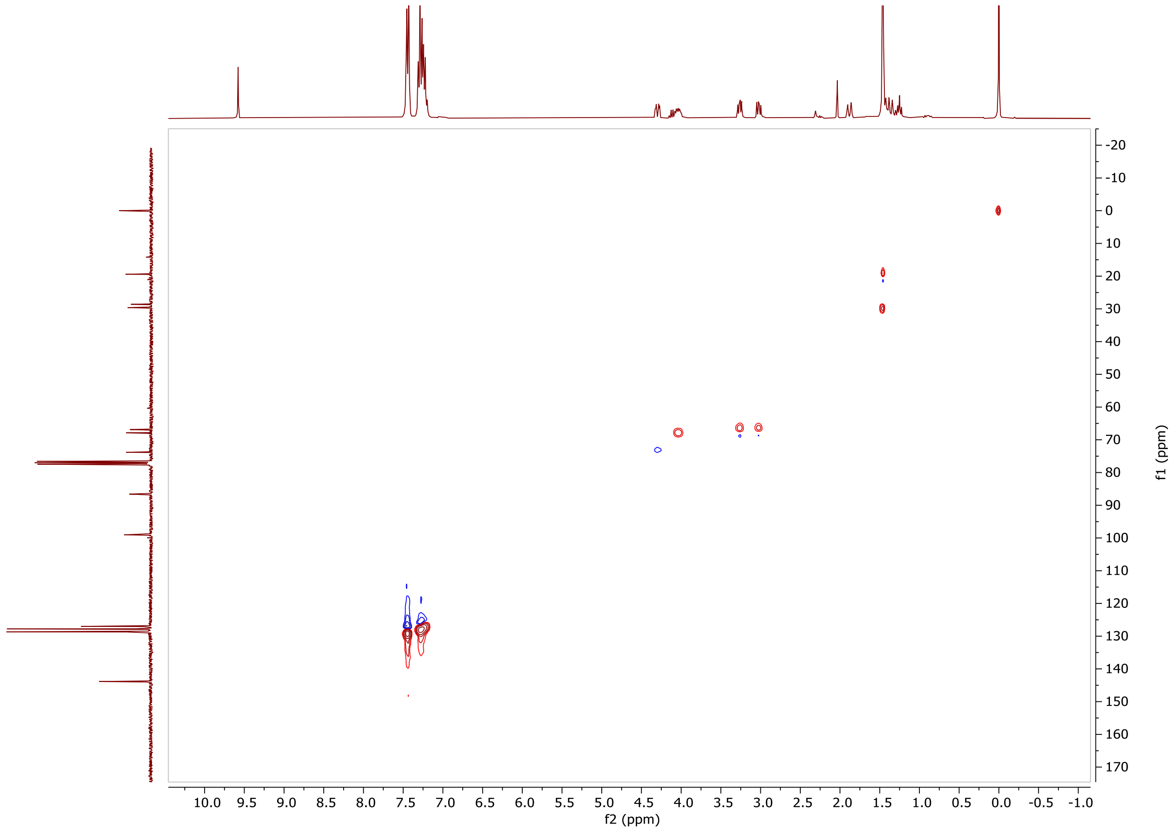

^{1}H NMR Spectrum of compound **19** (400 MHz, $CDCl_3$)

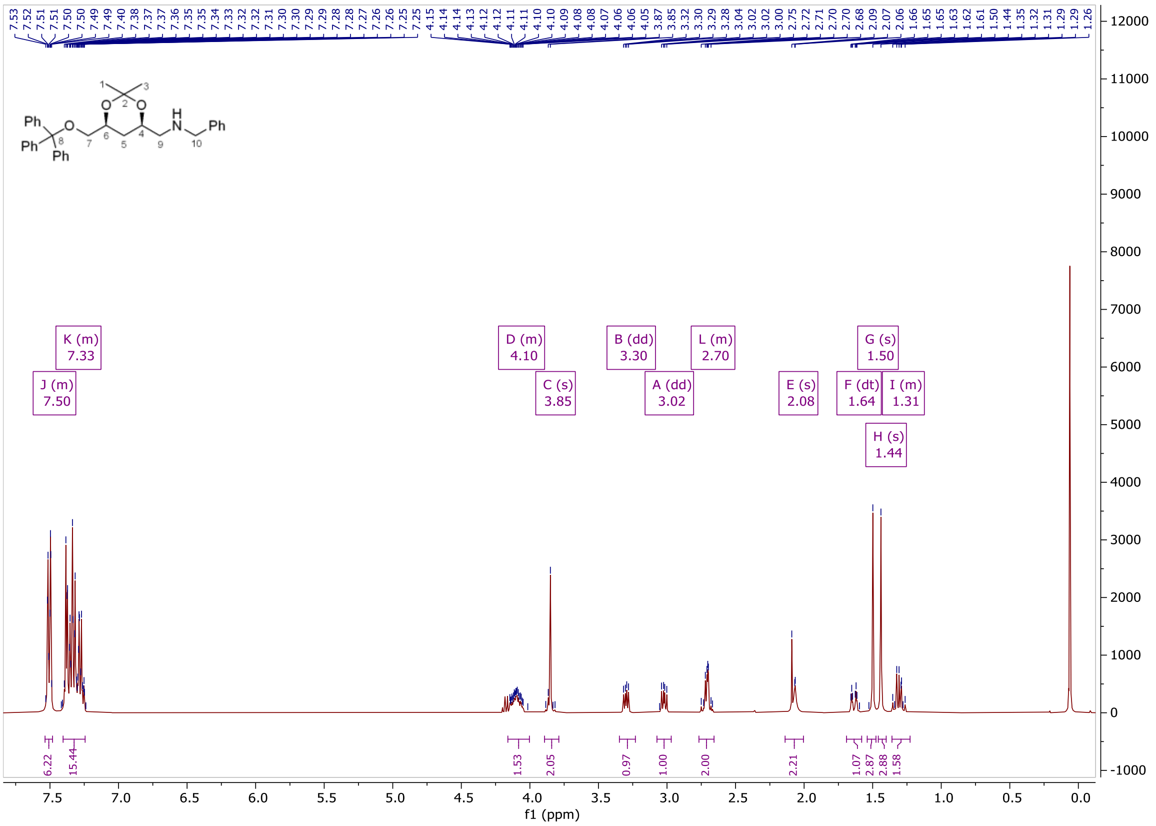

^{13}C NMR Spectrum of compound **19** (75 MHz, $CDCl_3$)

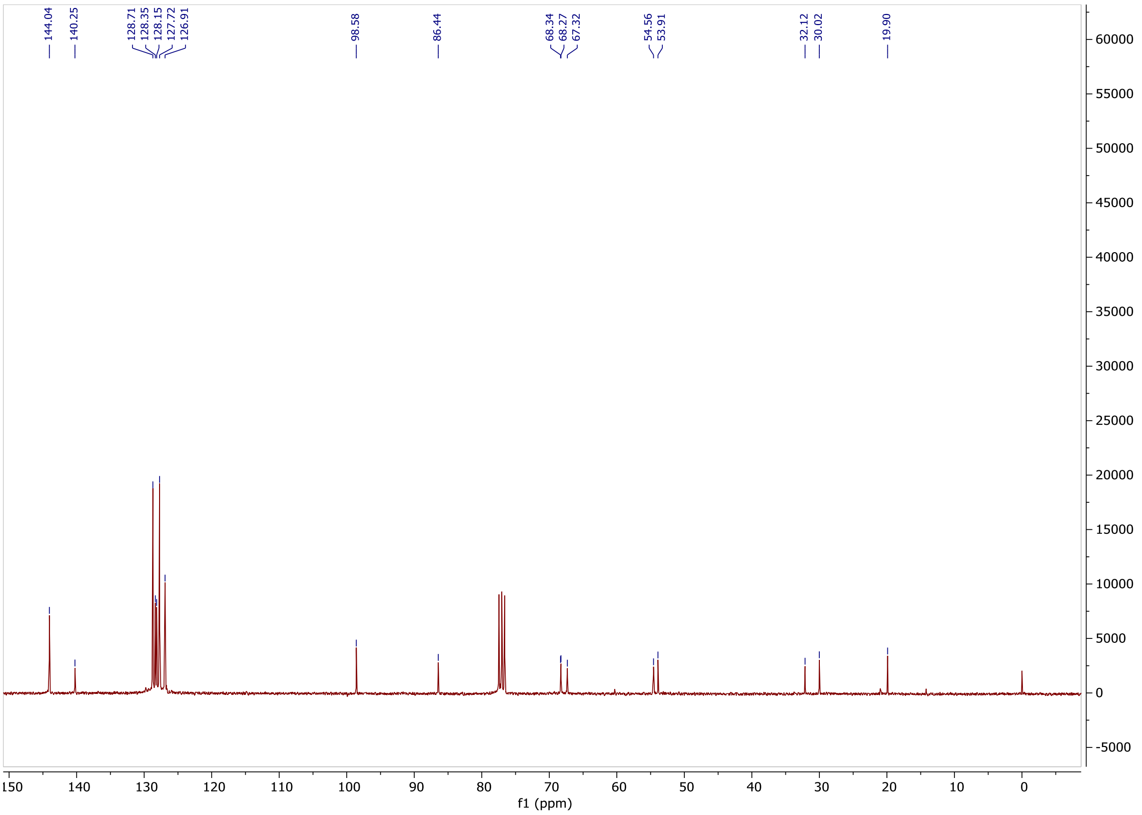

DEPT-135 Spectrum of compound **19**

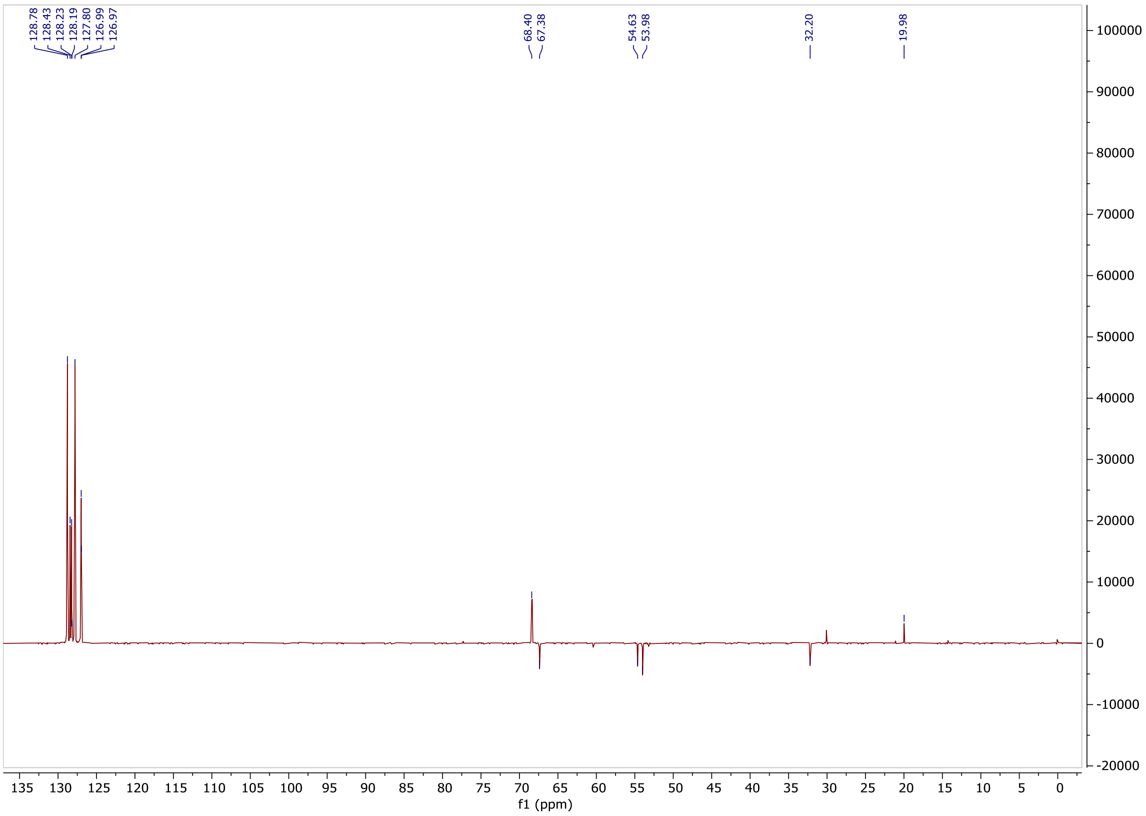

COSY Spectrum of compound **19**

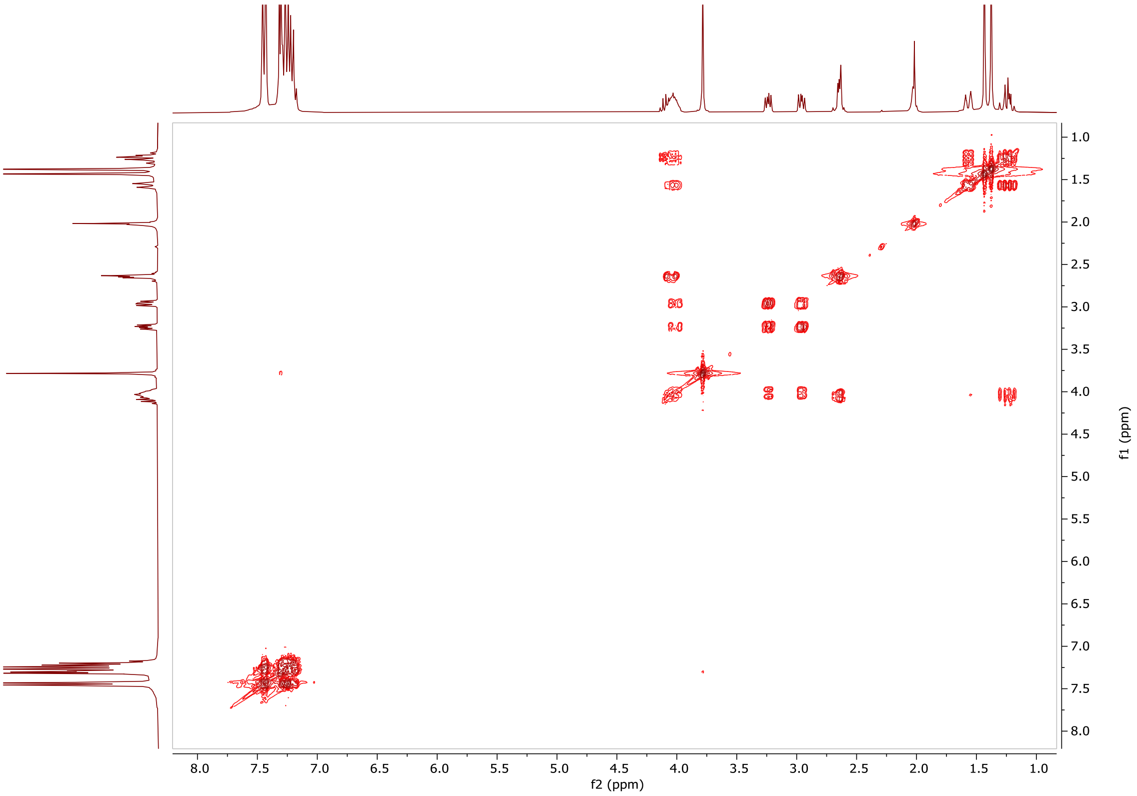

HSQC Spectrum of compound **19**

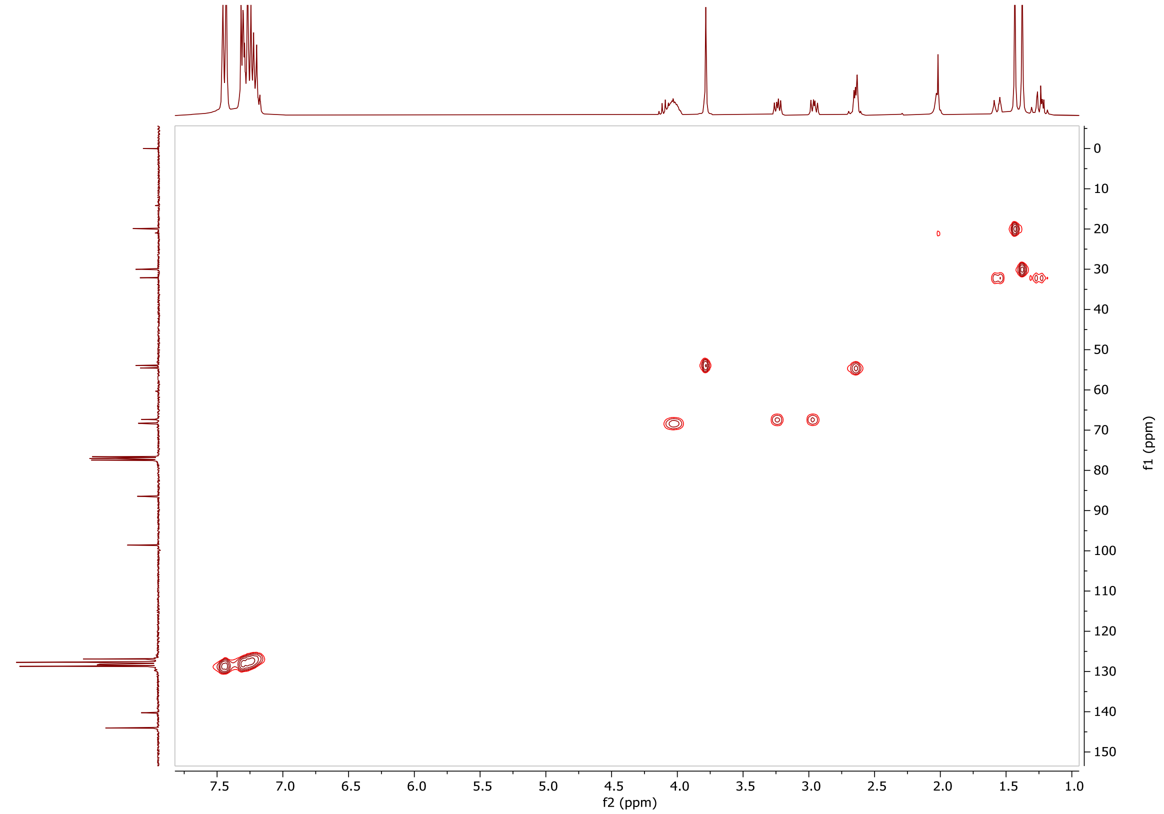

^{1}H NMR Spectrum of compound **20** (300 MHz, $CDCl_3$)

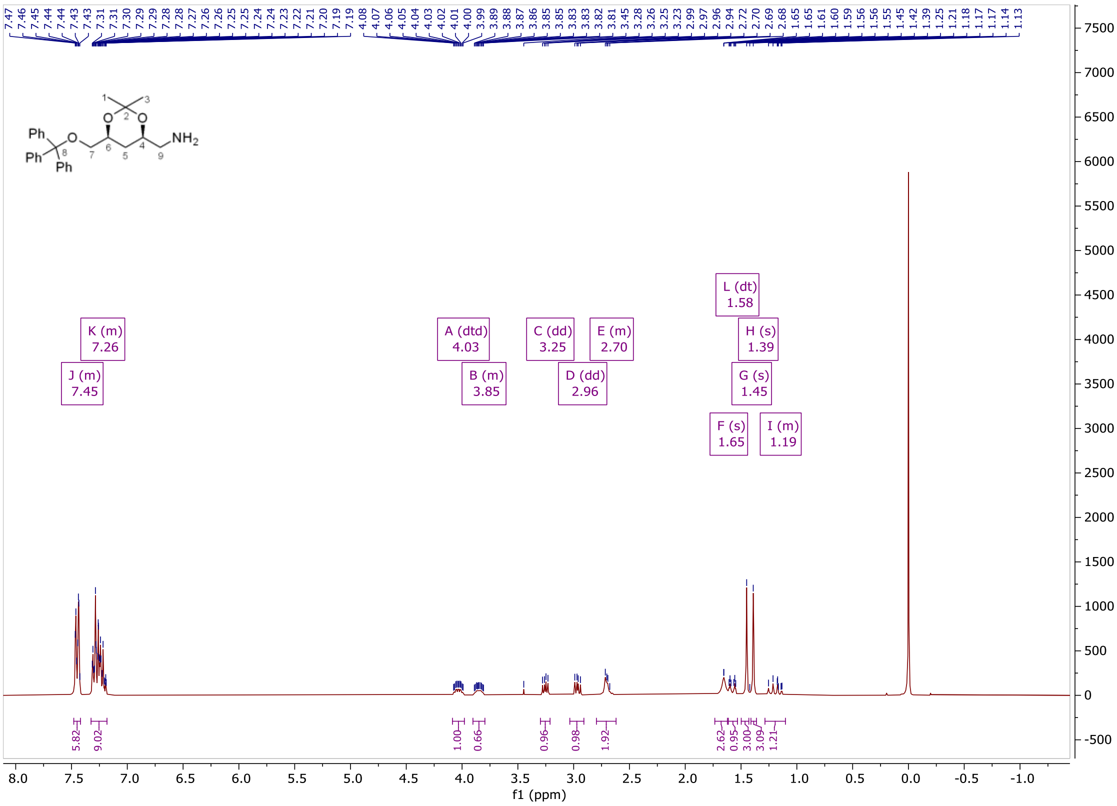

^{13}C NMR Spectrum of compound **20** (75 MHz, $CDCl_3$)

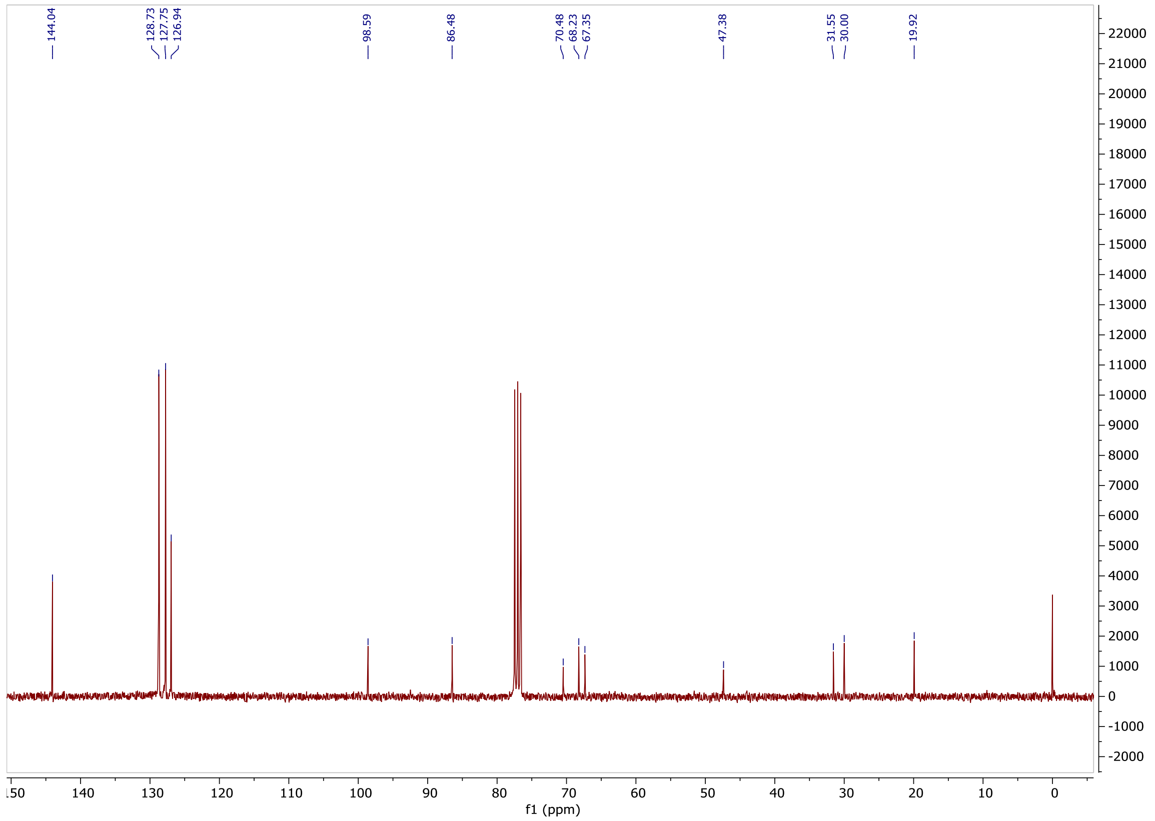

DEPT-135 Spectrum of compound **20**

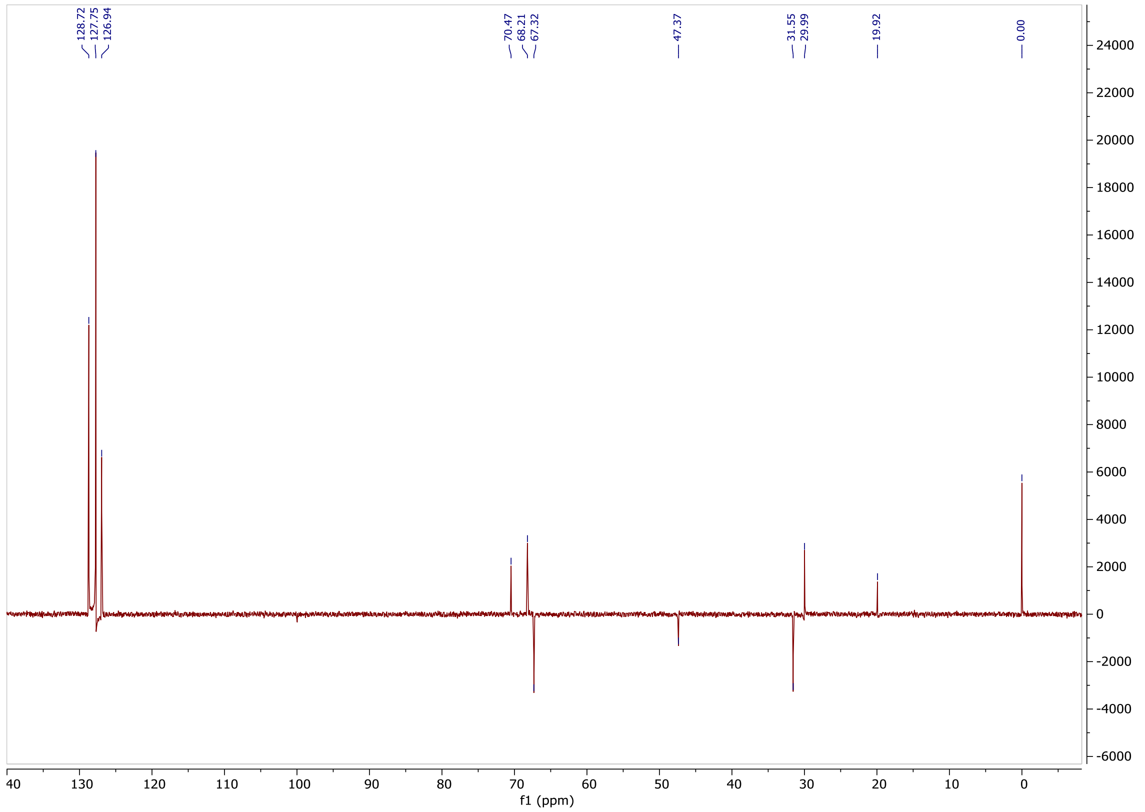

COSY Spectrum of compound **20**

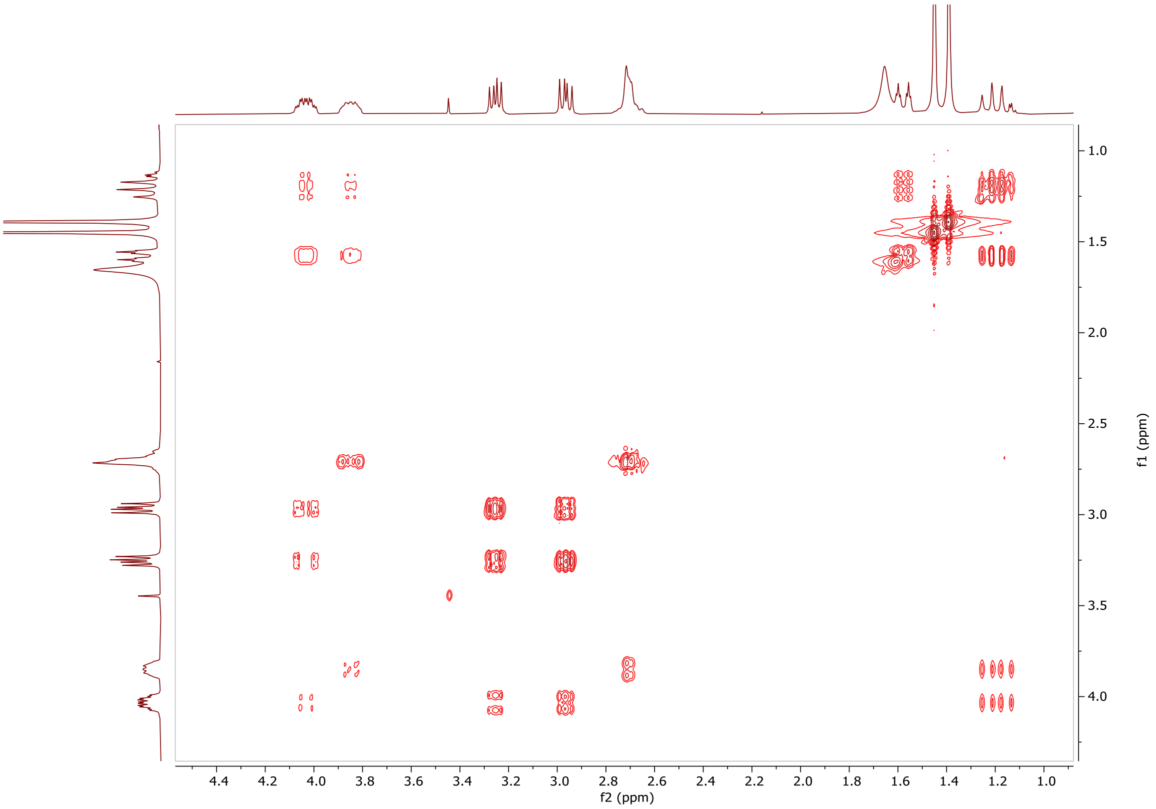

HSQC Spectrum of compound **20**

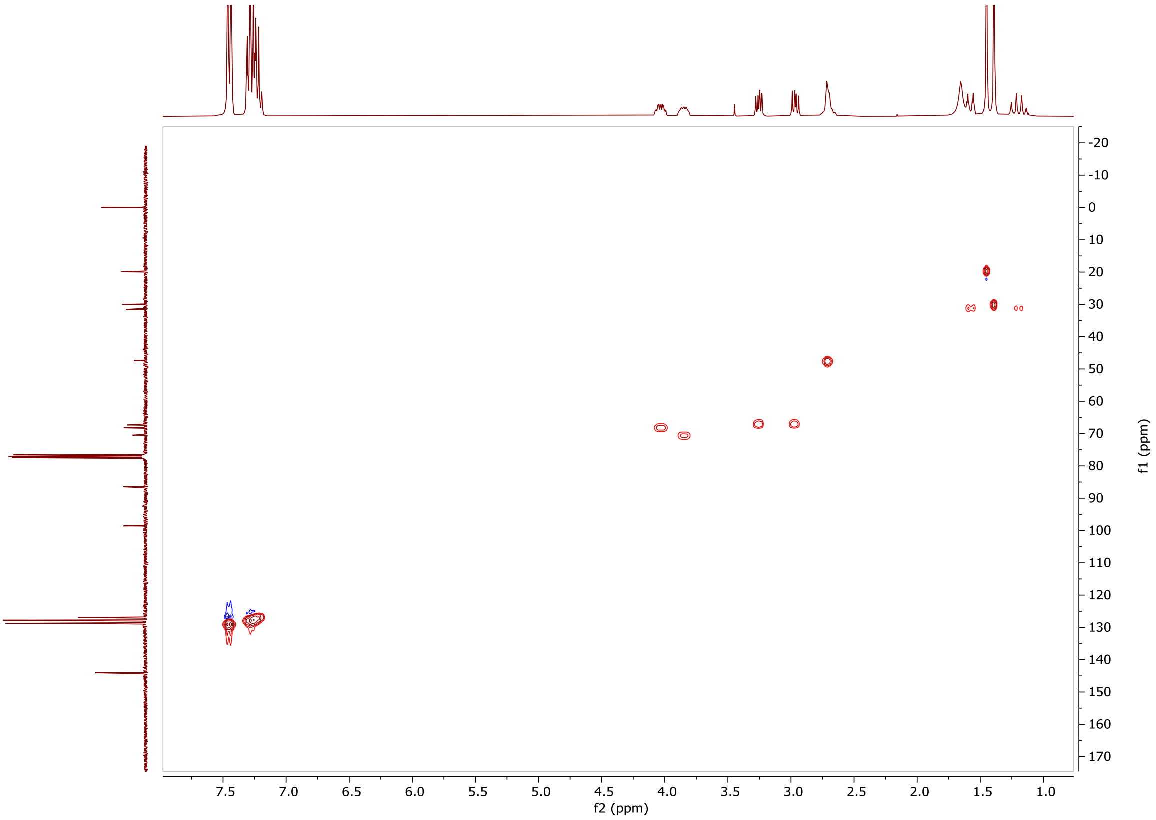

^{1}H NMR Spectrum of compound **21** (300 MHz, $CDCl_3$)

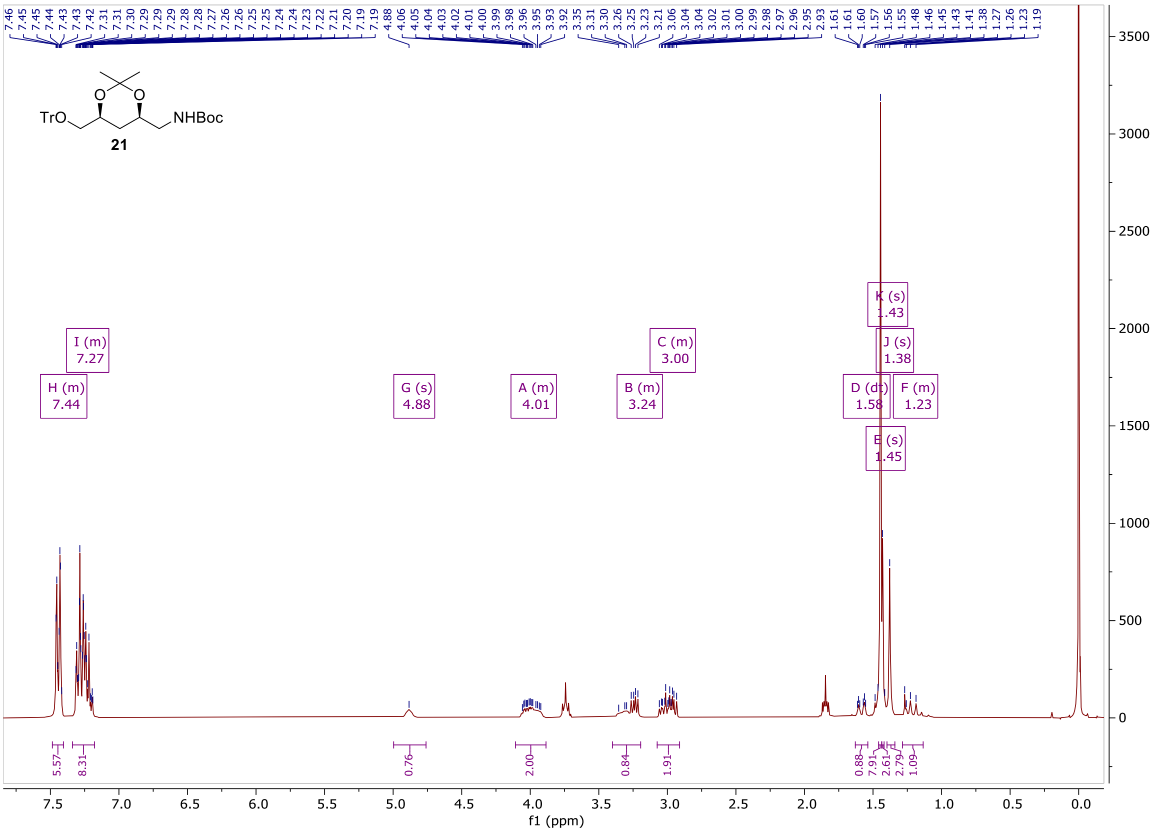

^{13}C NMR Spectrum of compound **21** (75 MHz, $CDCl_3$)

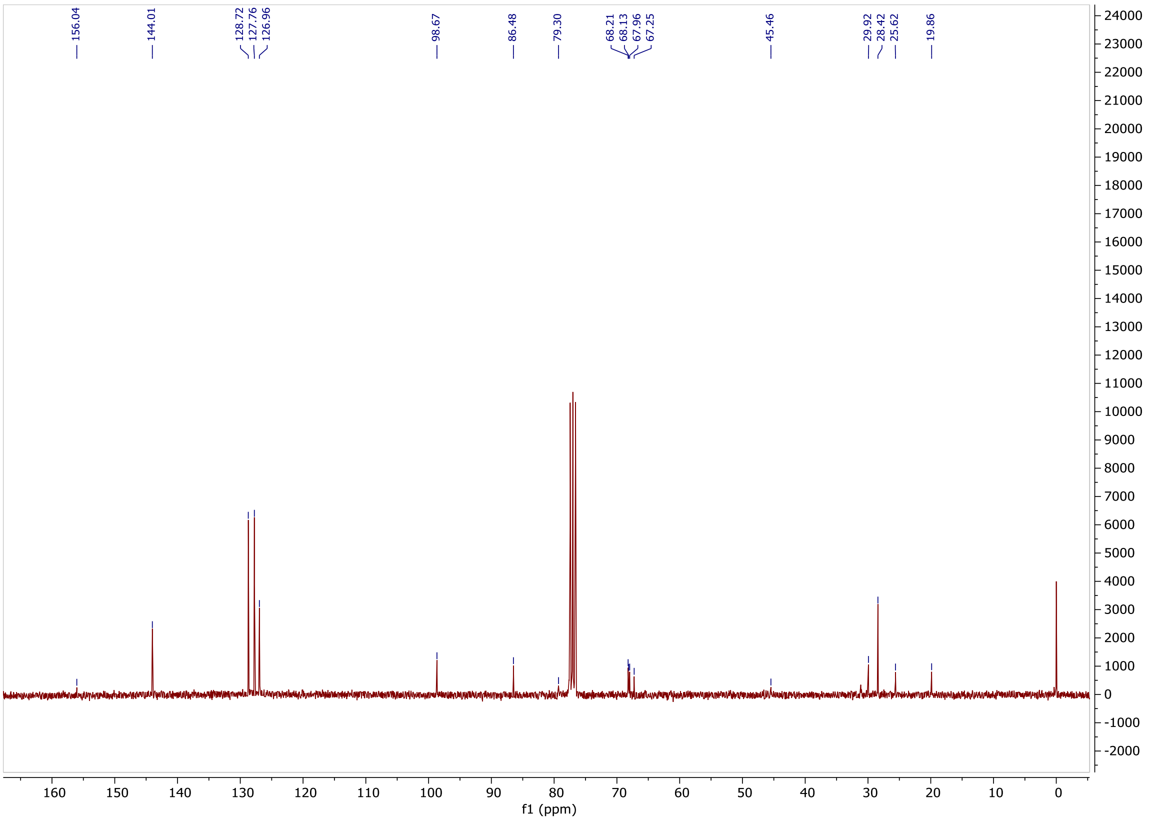

DEPT-135 Spectrum of compound **21**

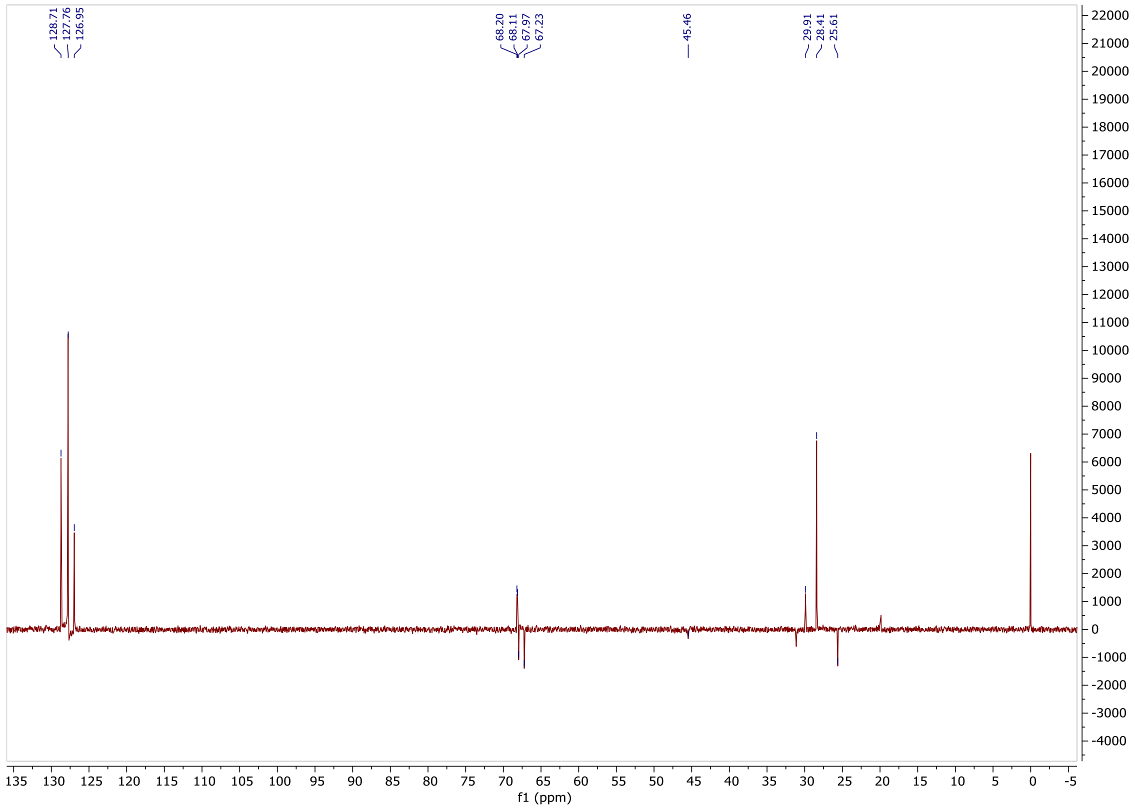

COSY Spectrum of compound **21**

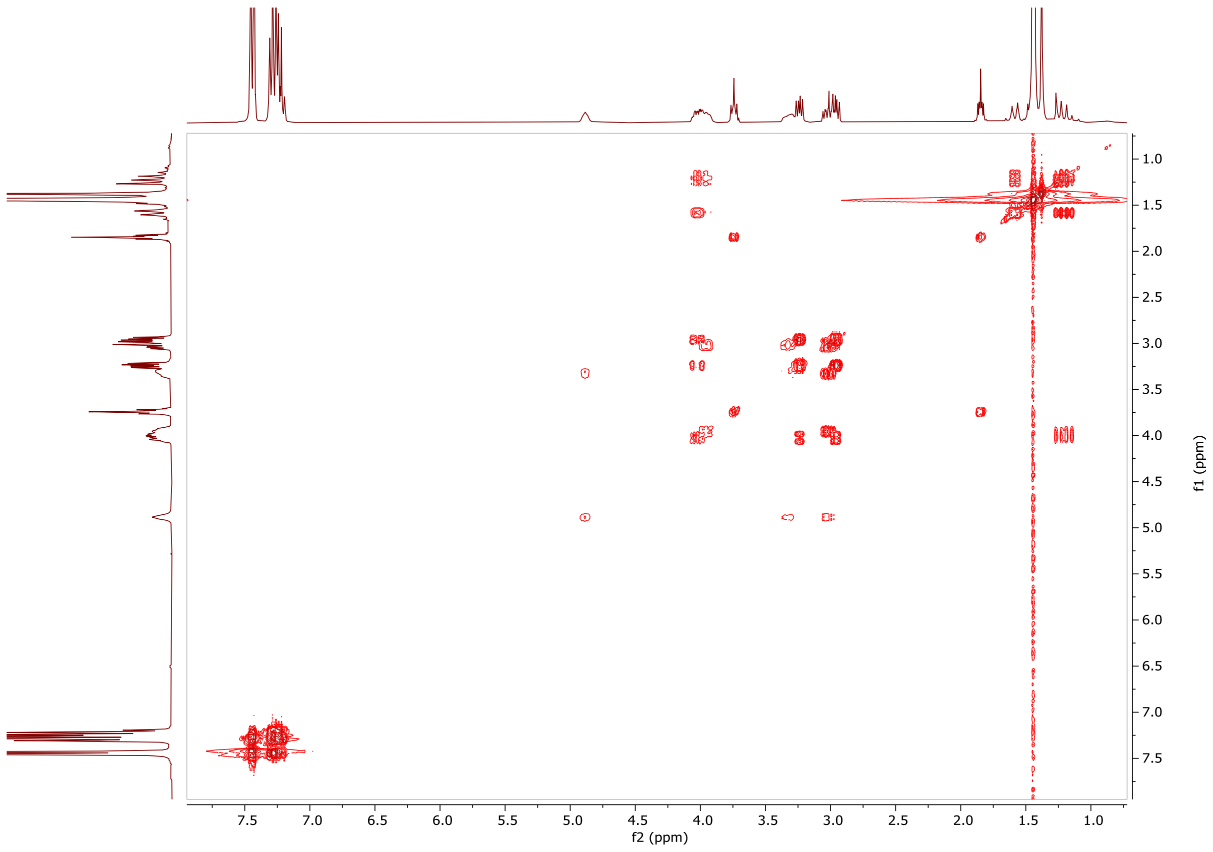

HSQC Spectrum of compound **21**

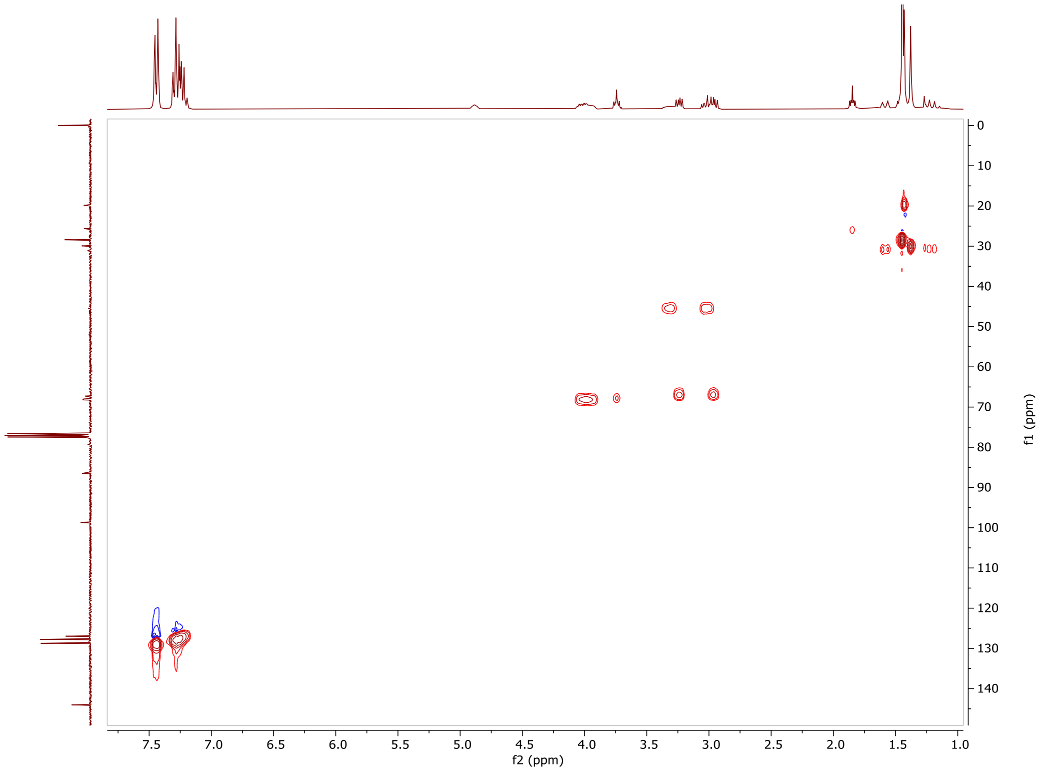

^{1}H NMR Spectrum of compound **22** (300 MHz, $CDCl_3$)

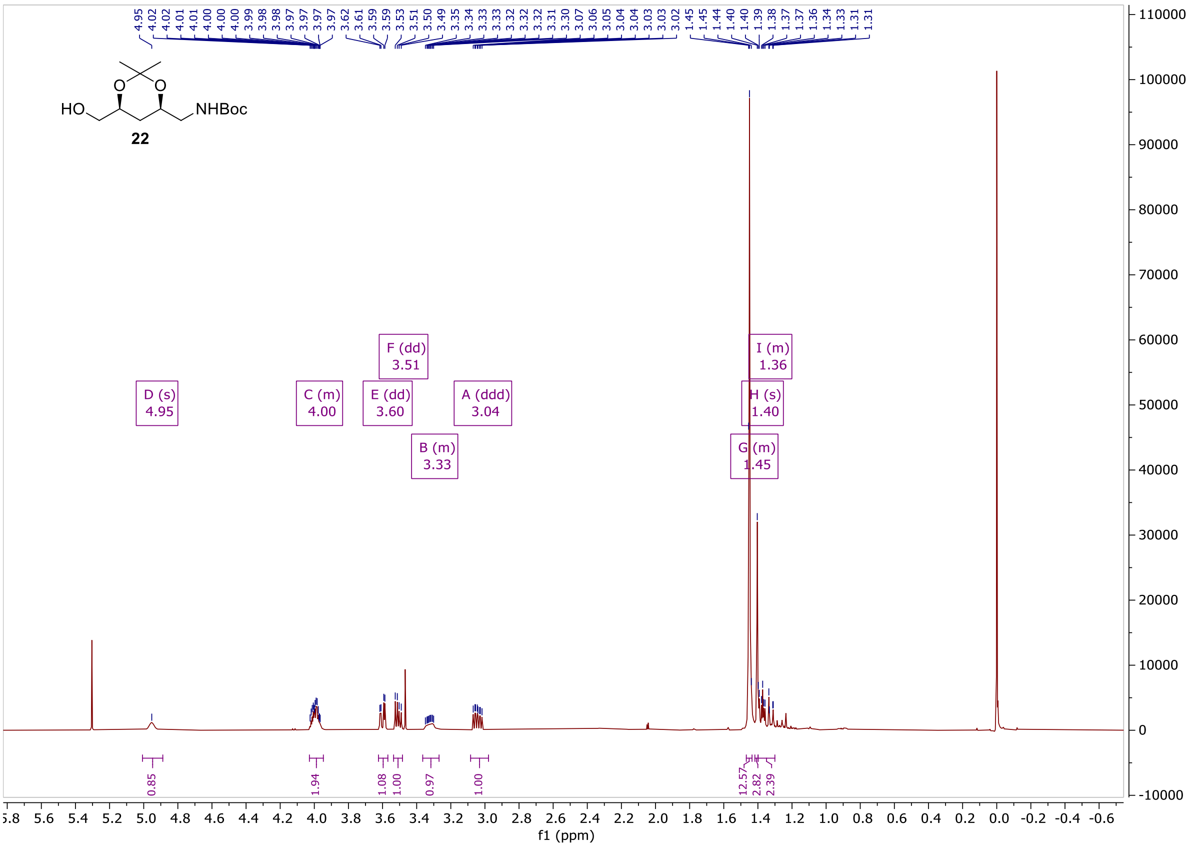

^{13}C NMR Spectrum of compound **22** (75 MHz, $CDCl_3$)

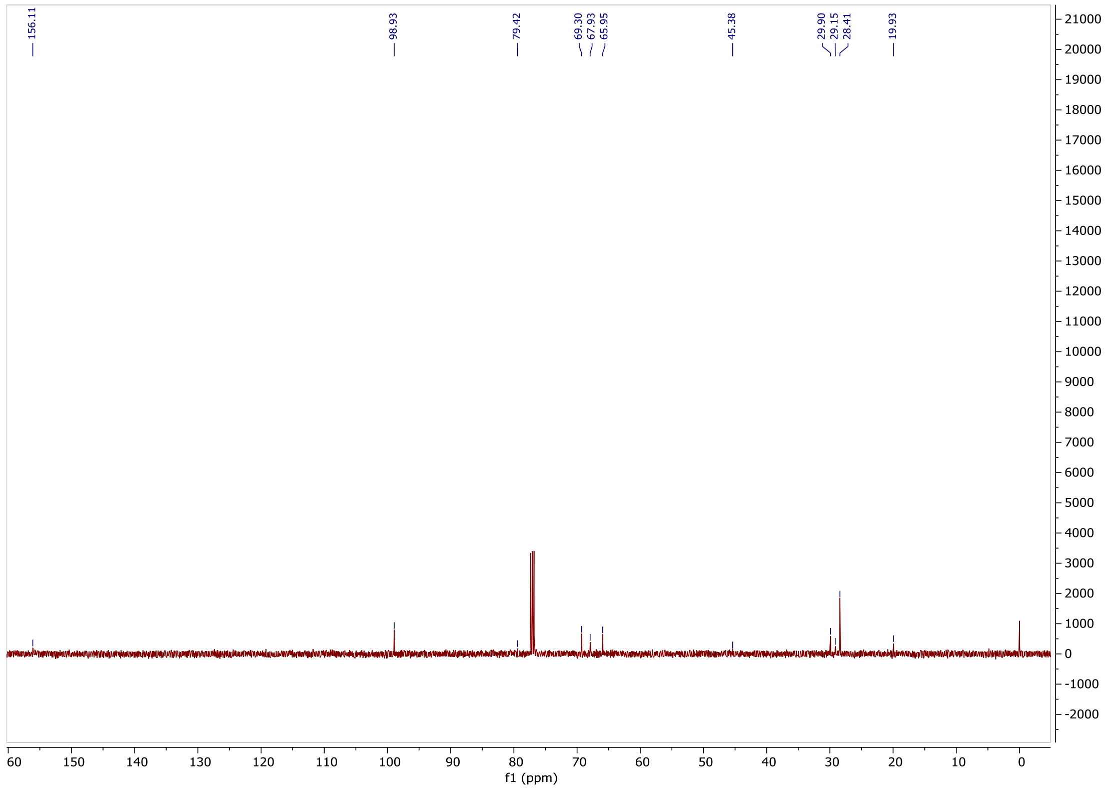

^{1}H NMR Spectrum of compound **23** (300 MHz, $CDCl_3$)

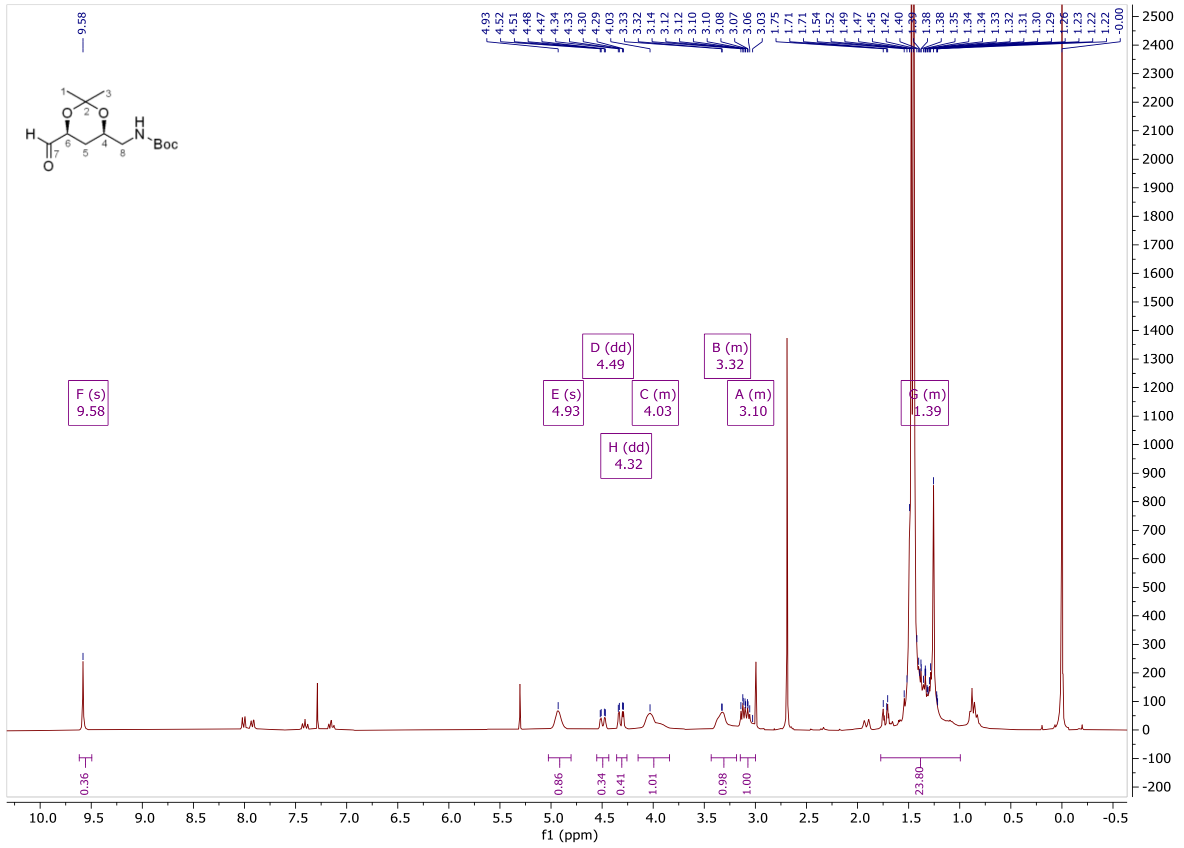

^{1}H NMR Spectrum of compound **24** (300 MHz, $CDCl_3$)

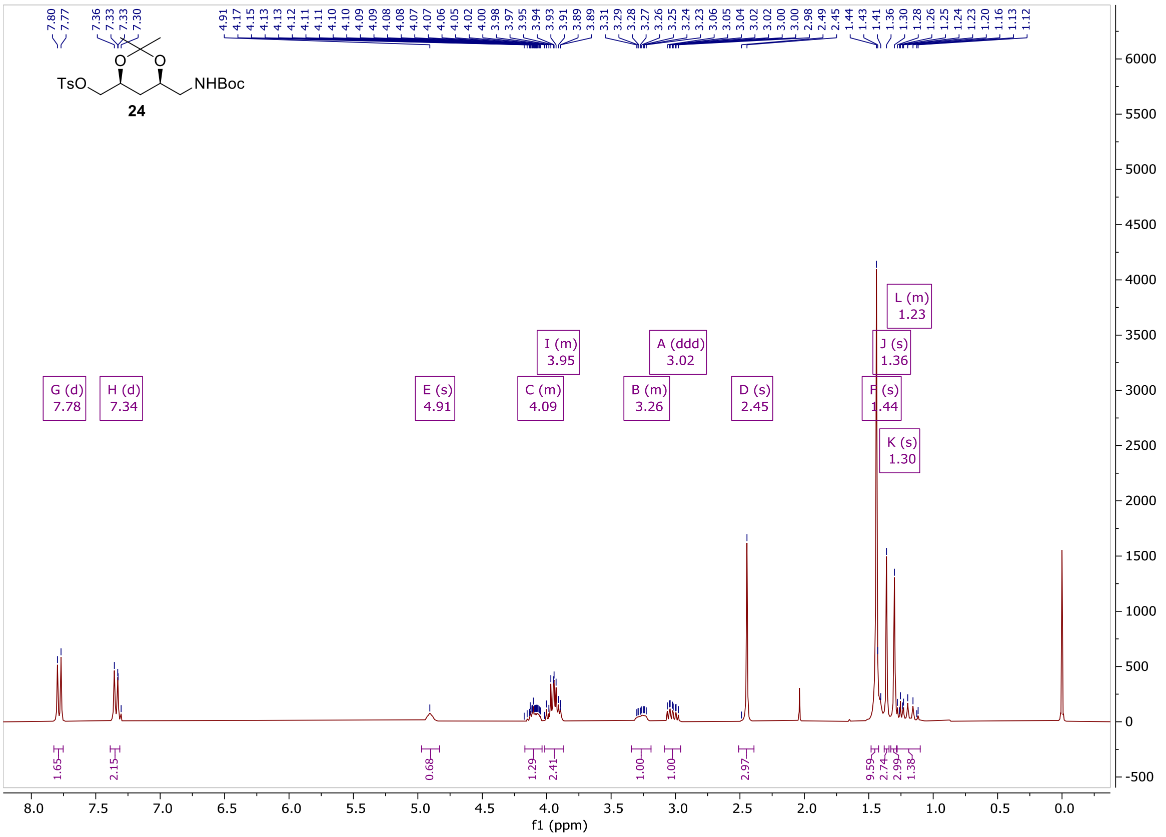

^{13}C NMR Spectrum of compound **24** (75 MHz, $CDCl_3$)

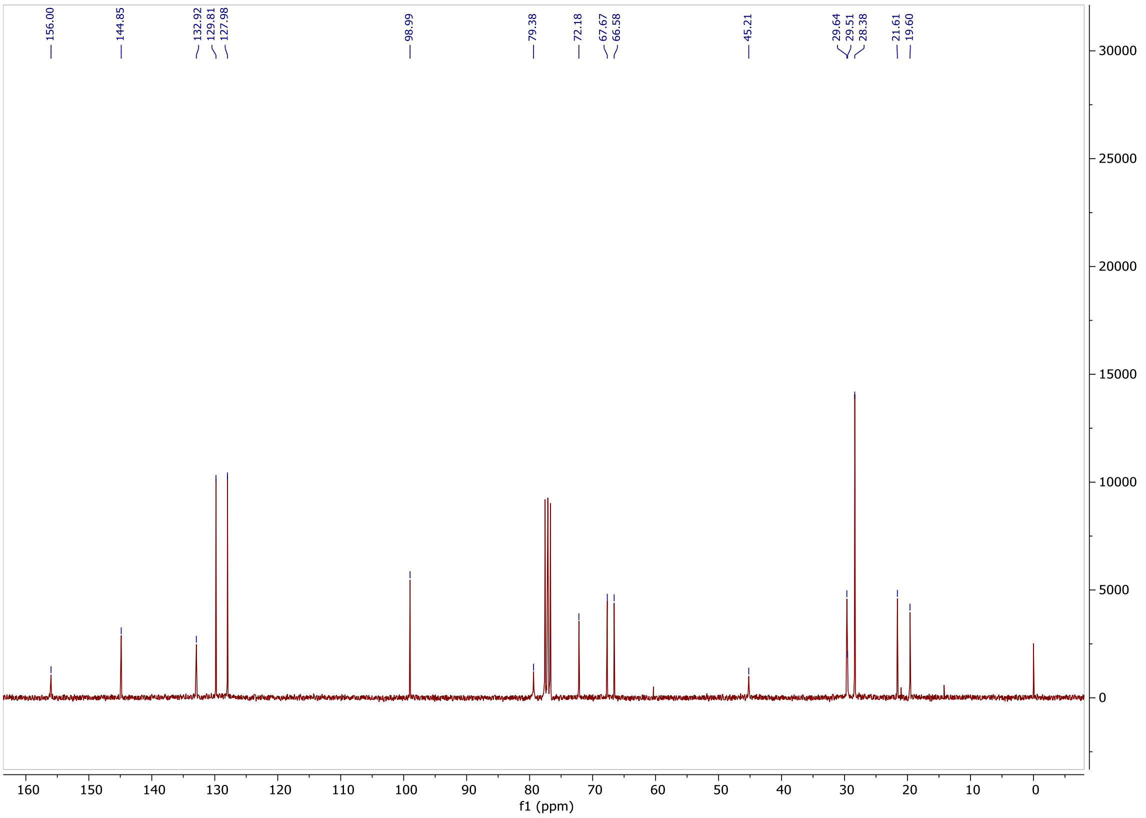

DEPT-135 Spectrum of compound **24**

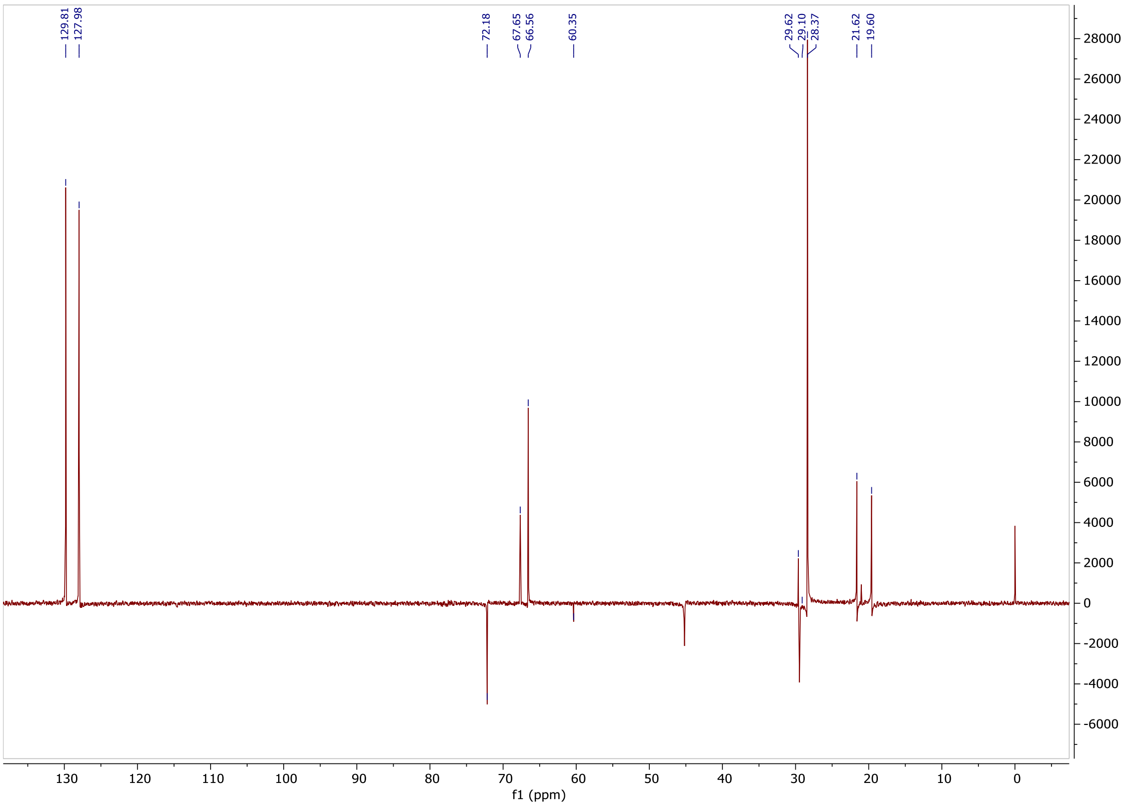

HSQC Spectrum of compound **24**

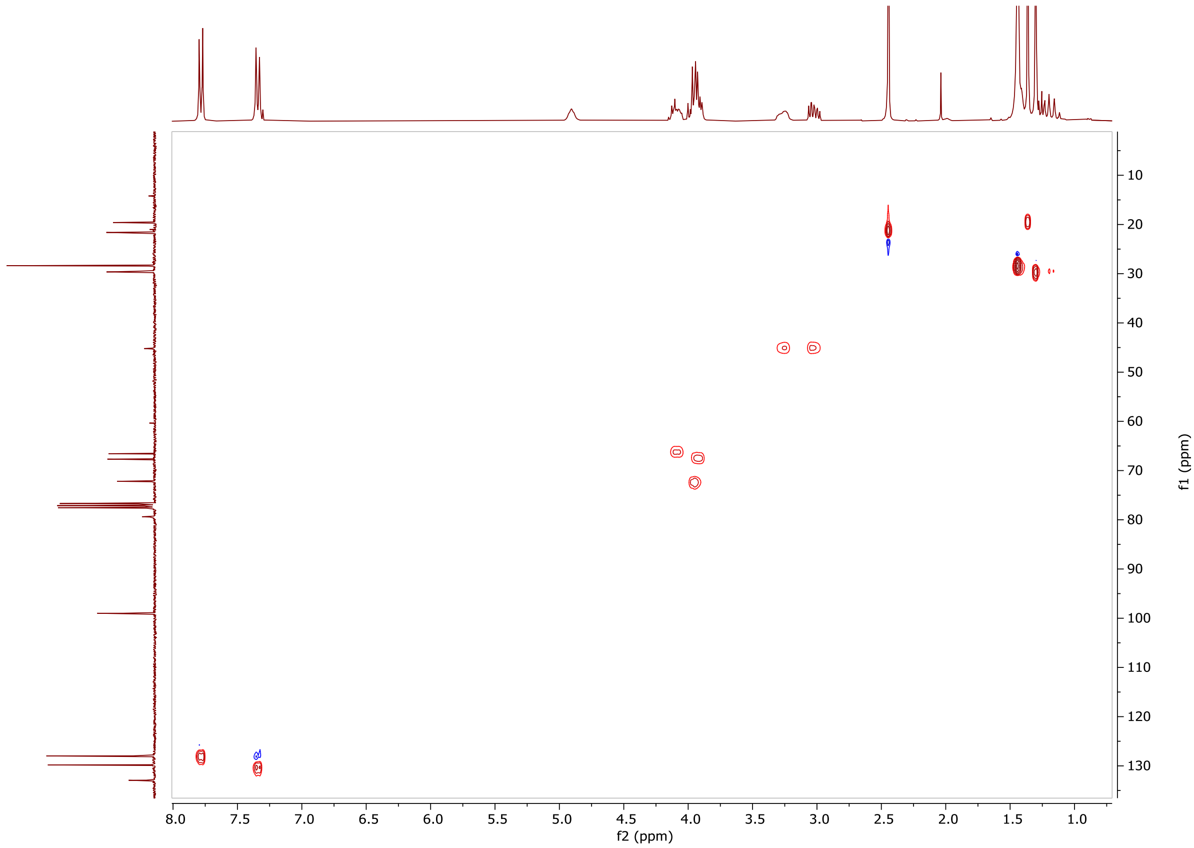

^{1}H NMR Spectrum of compound **25** (300 MHz, CDCl$_3$)

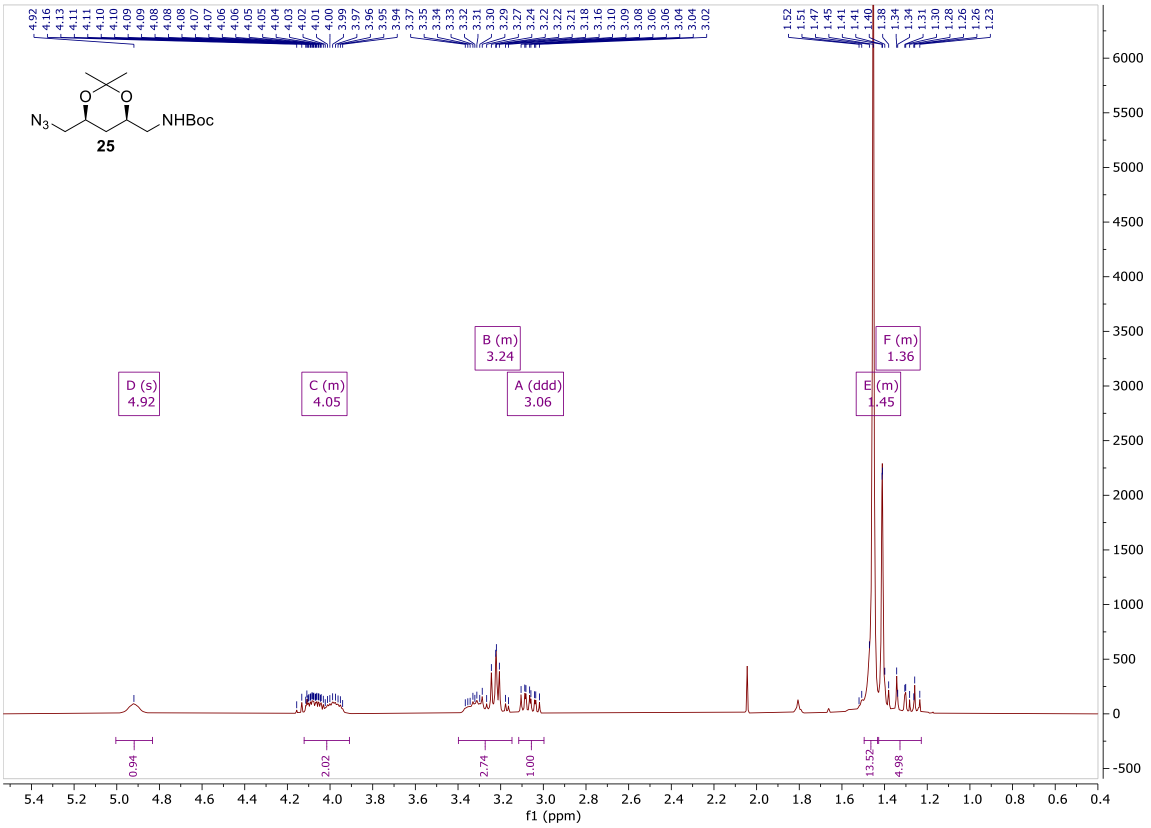

^{13}C NMR Spectrum of compound **25** (75 MHz, CDCl$_3$)

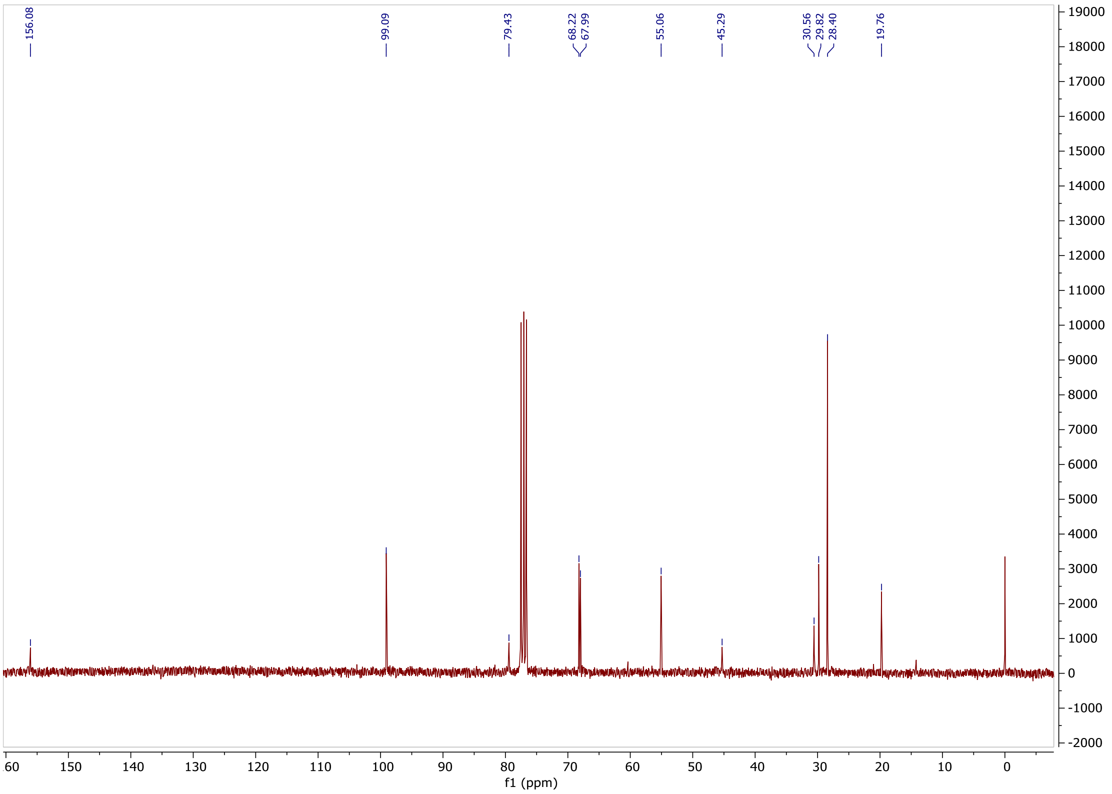

COSY Spectrum of compound **25**

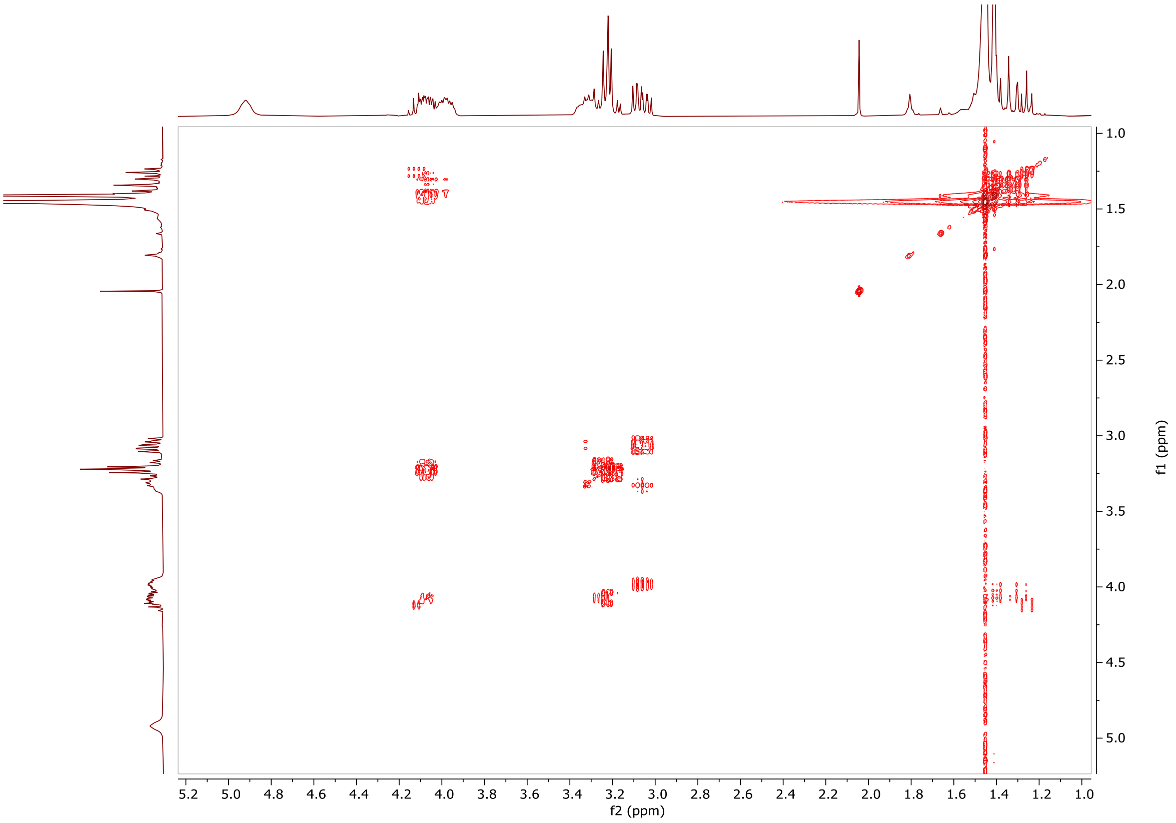

HSQC Spectrum of compound **25**

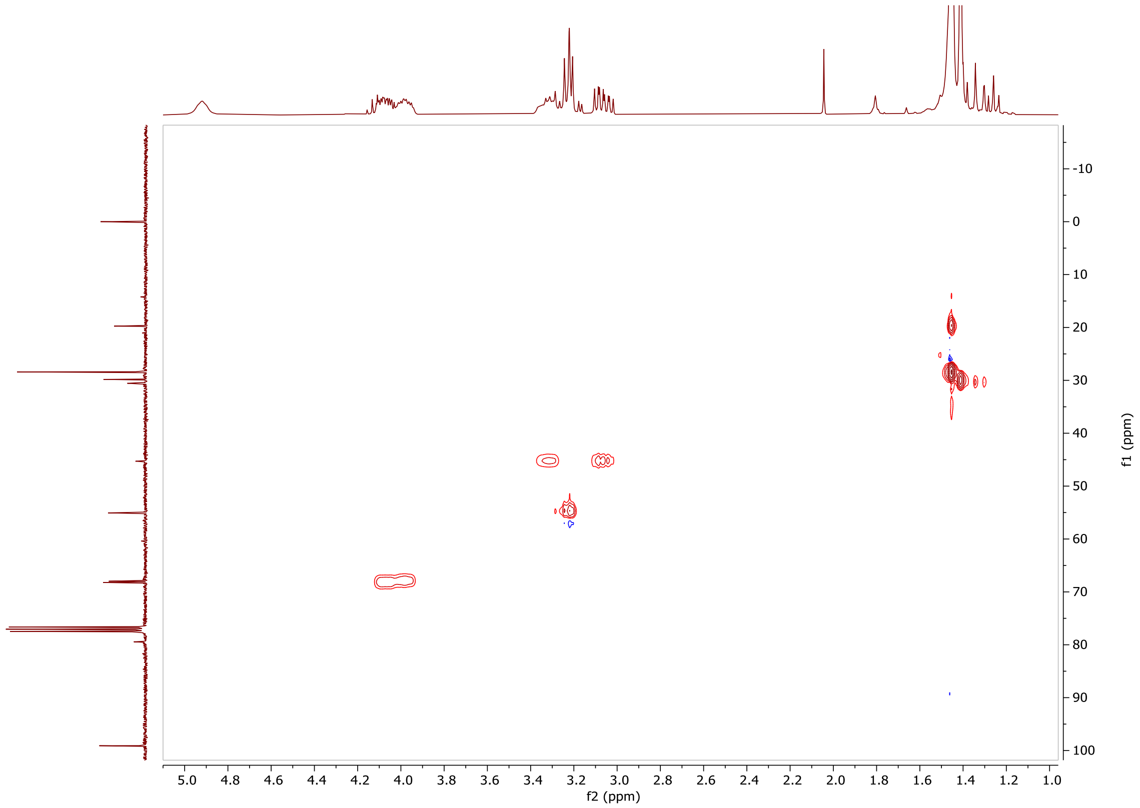

^{1}H NMR Spectrum of compound **26** (300 MHz, $CDCl_3$)

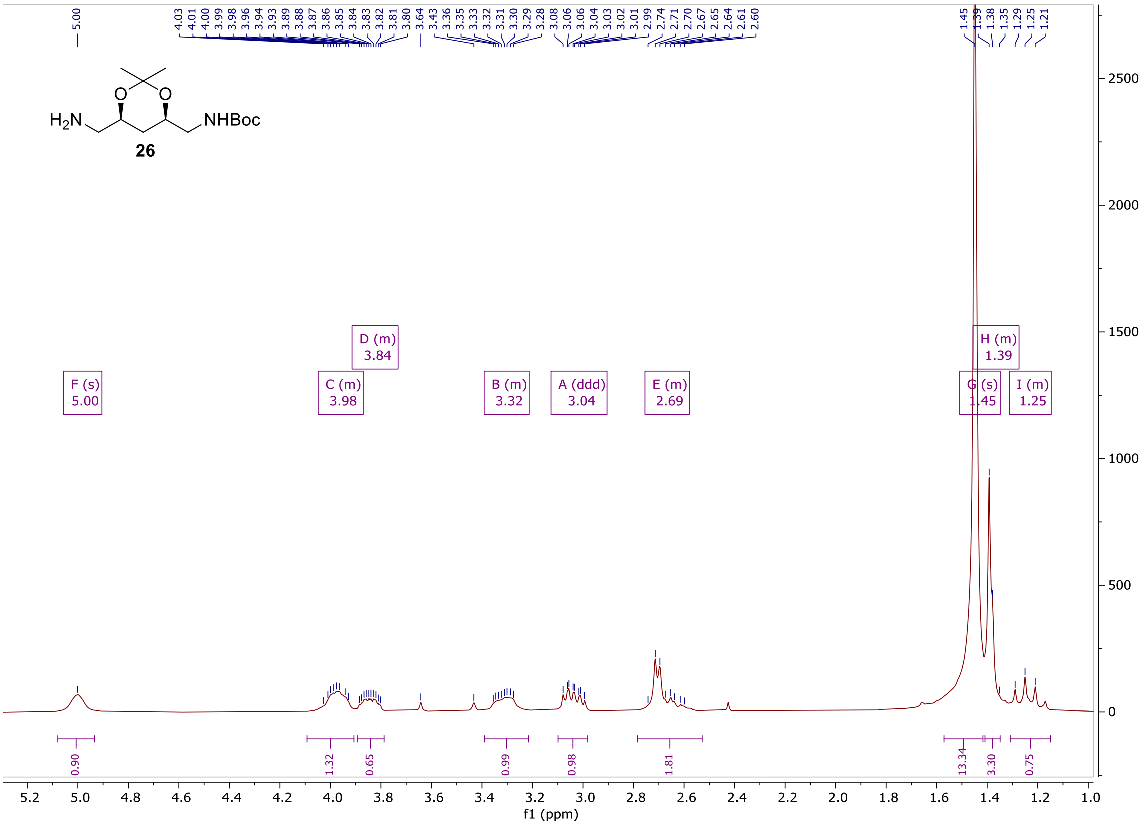

^{13}C NMR Spectrum of compound **26** (75 MHz, $CDCl_3$)

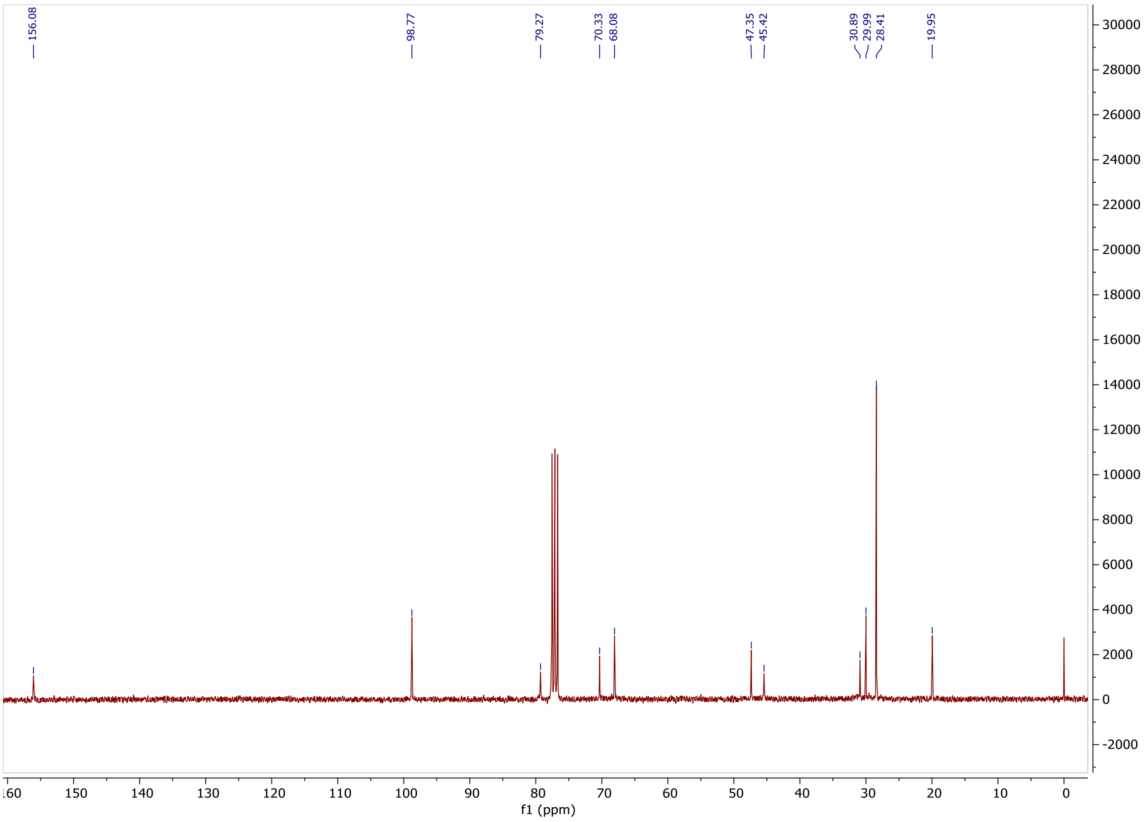

DEPT Spectrum of compound **26**

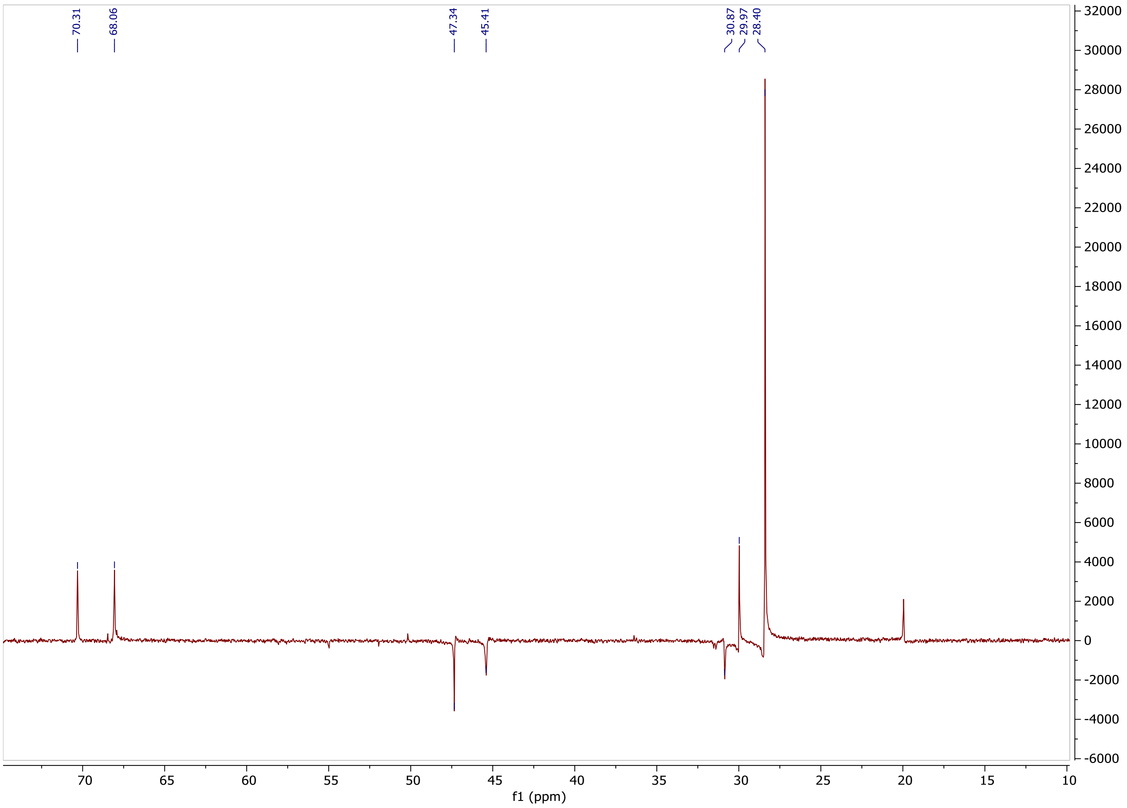

COSY Spectrum of compound **26**

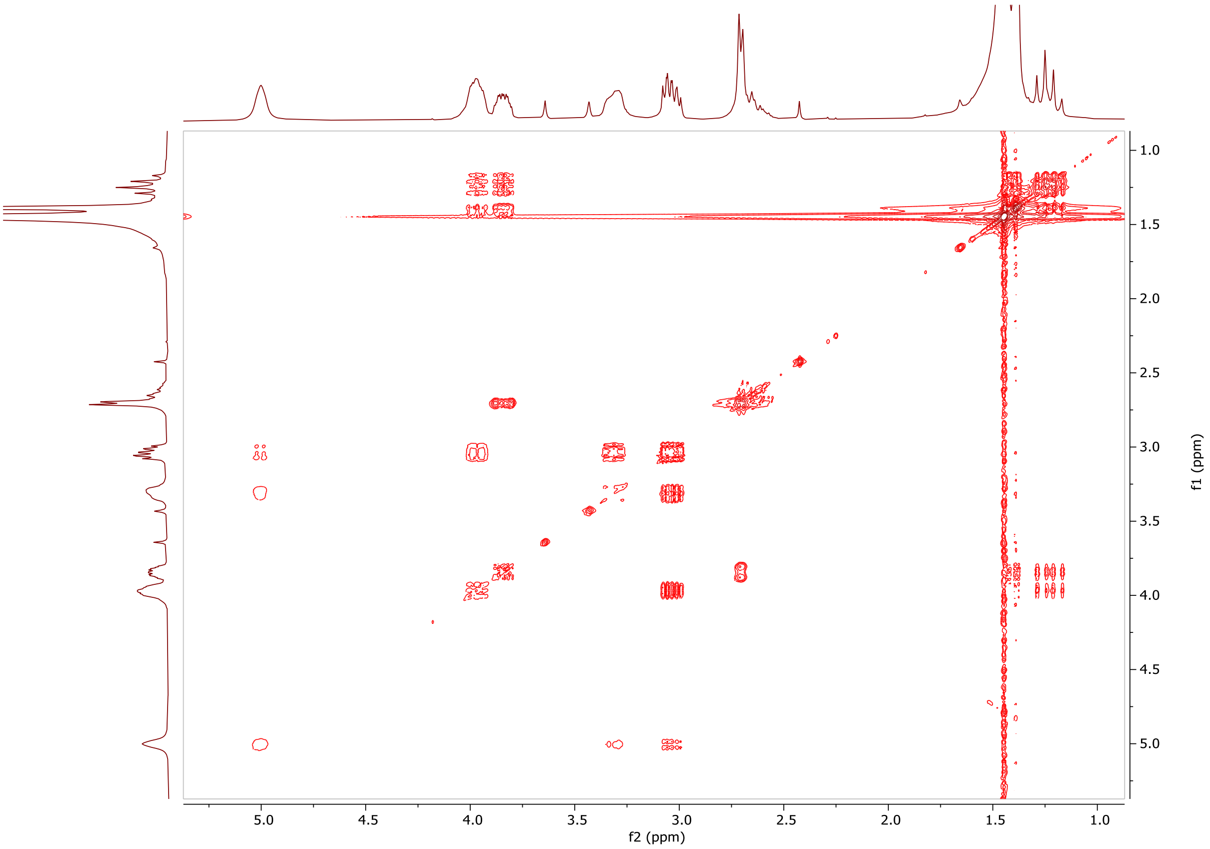

HSQC Spectrum of compound **26**

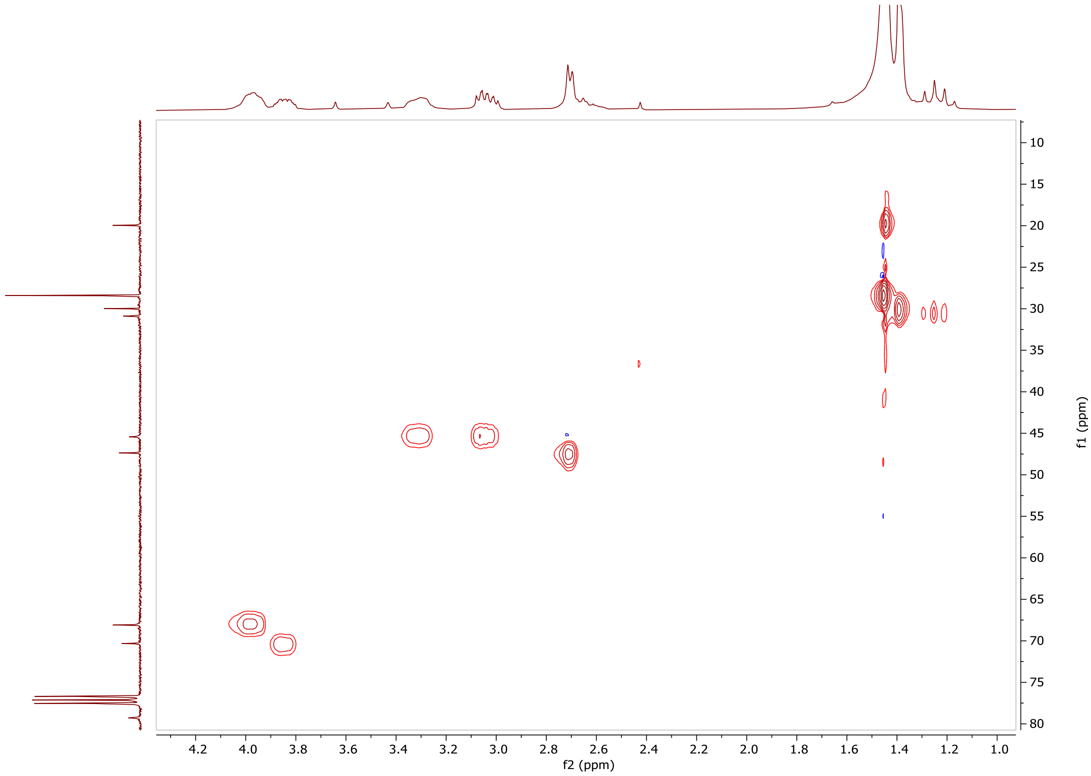

^{1}H NMR Spectrum of compound **27** (500 MHz, $CDCl_3$)

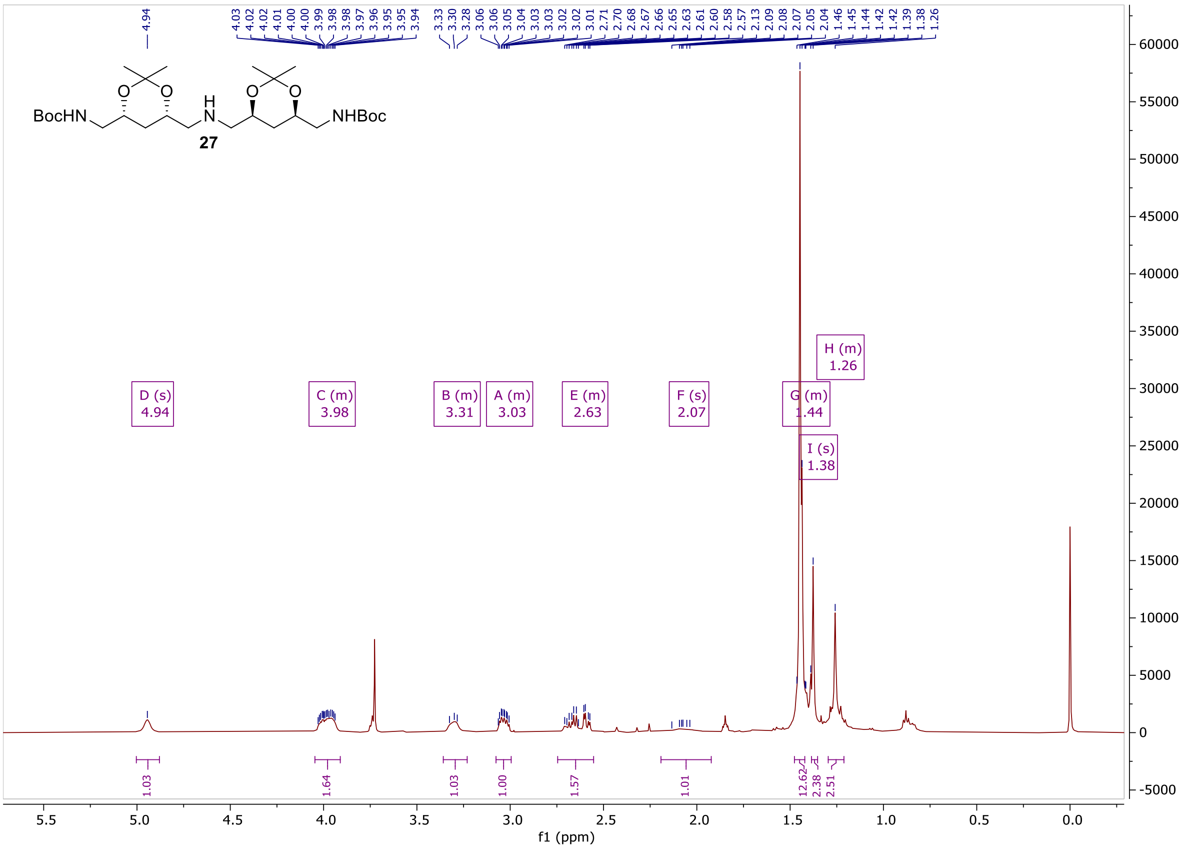

^{13}C NMR Spectrum of compound **27** (126 MHz, $CDCl_3$)

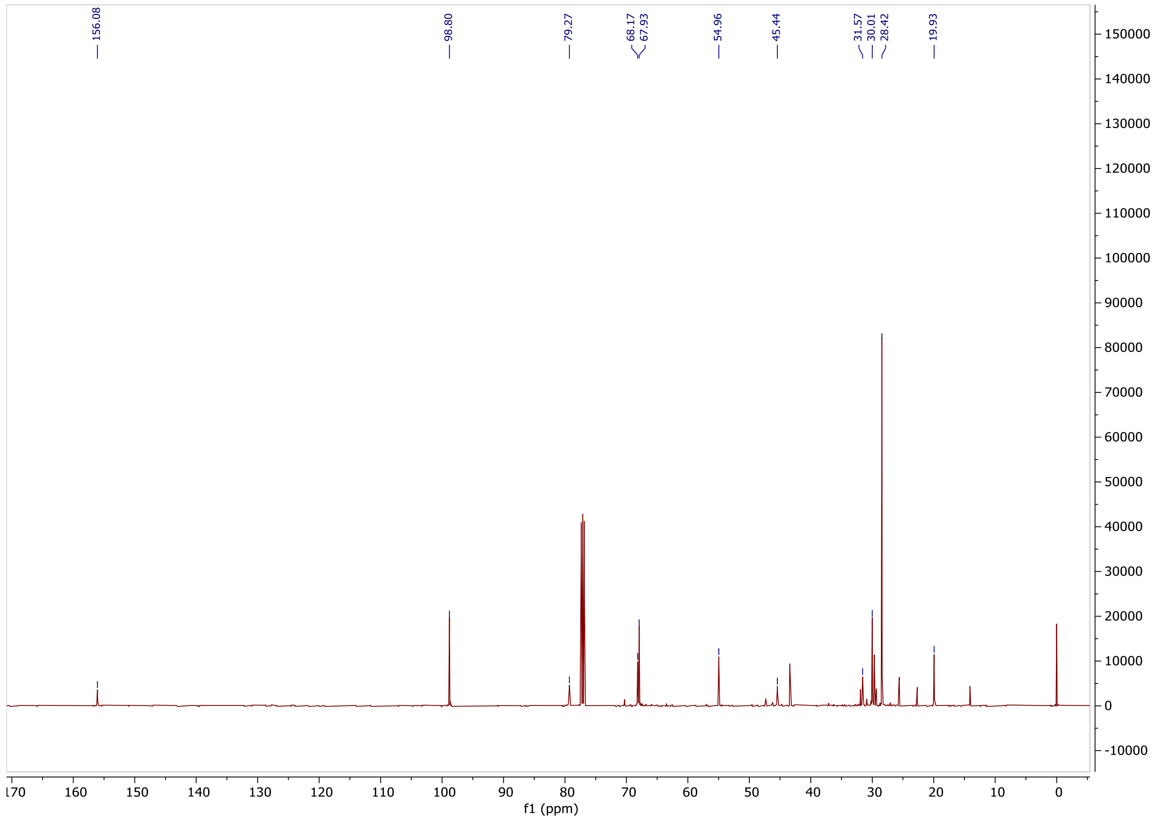

HSQC Spectrum of compound **27**

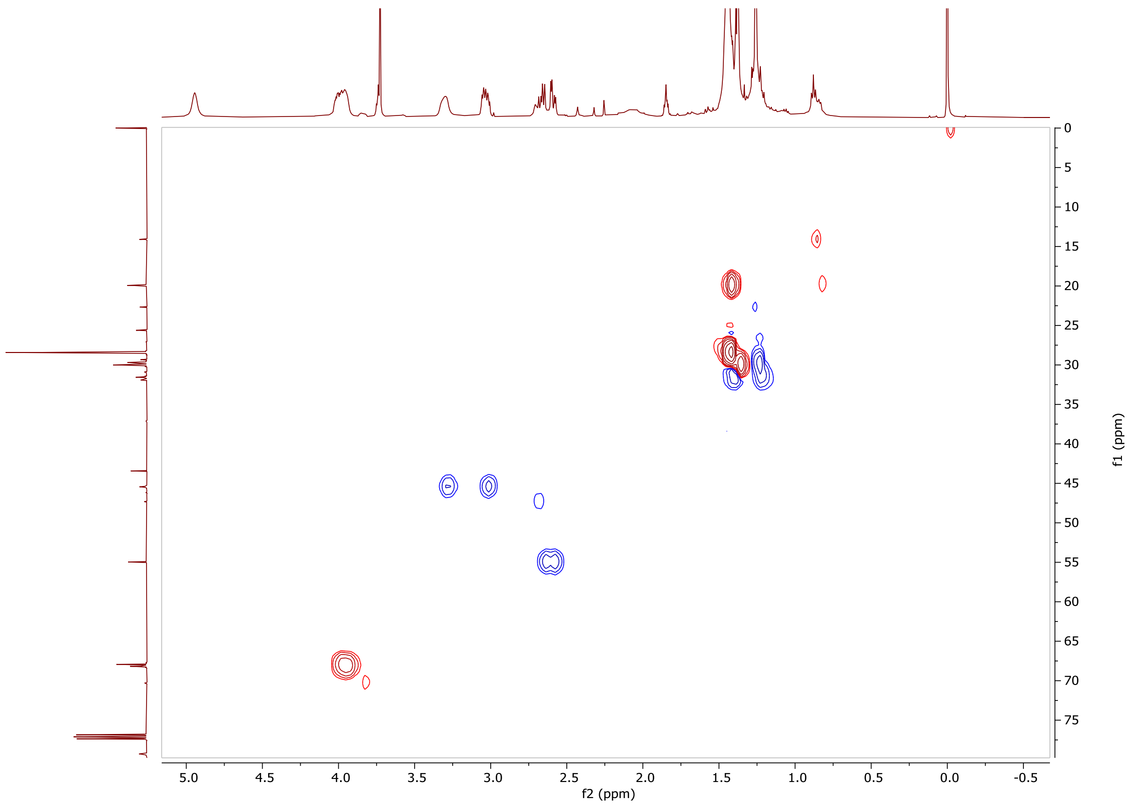

^{1}H NMR Spectrum of compound **28** (500 MHz, MeOD)

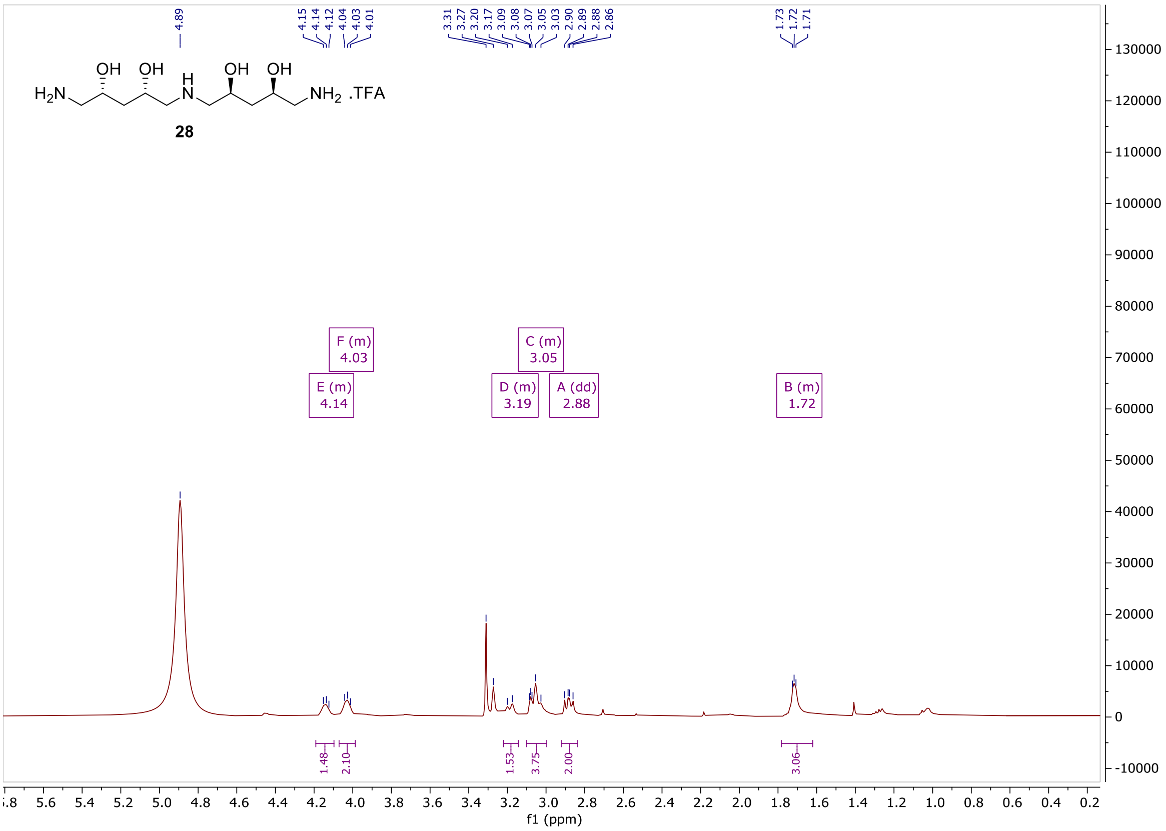

^{13}C NMR Spectrum of compound **28** (126 MHz, MeOD)

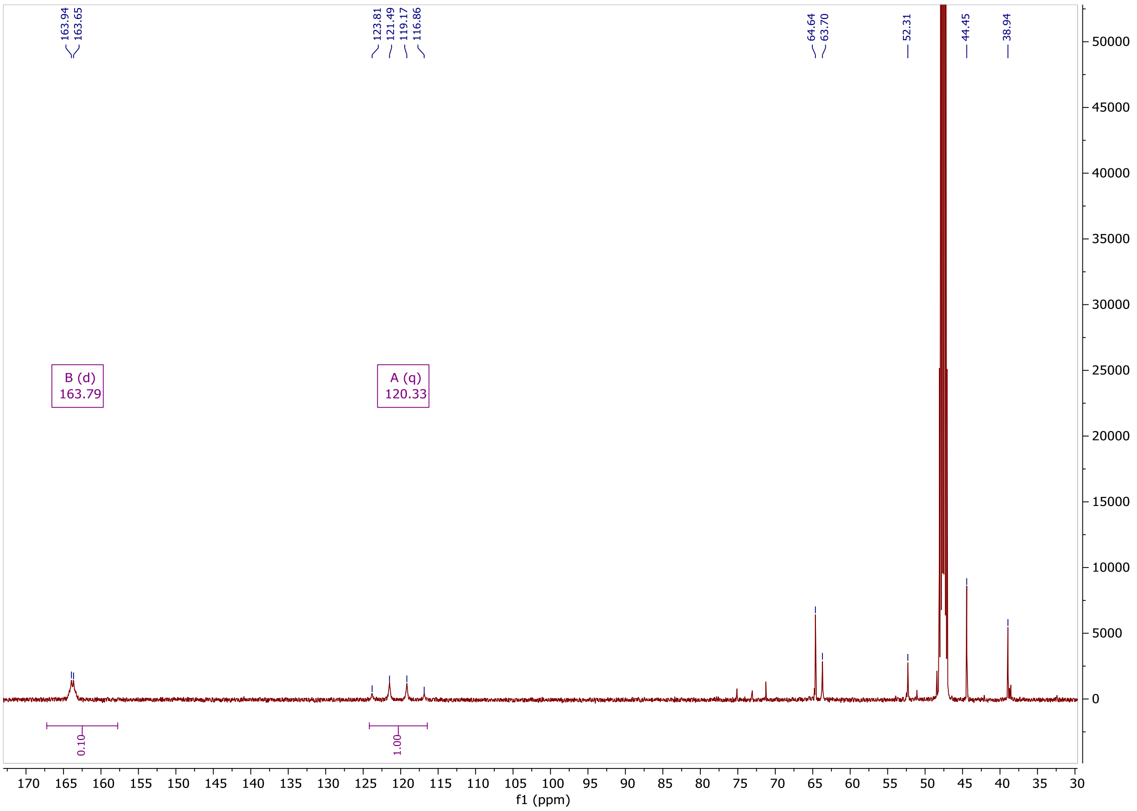

DEPT-135 Spectrum of compound **28**

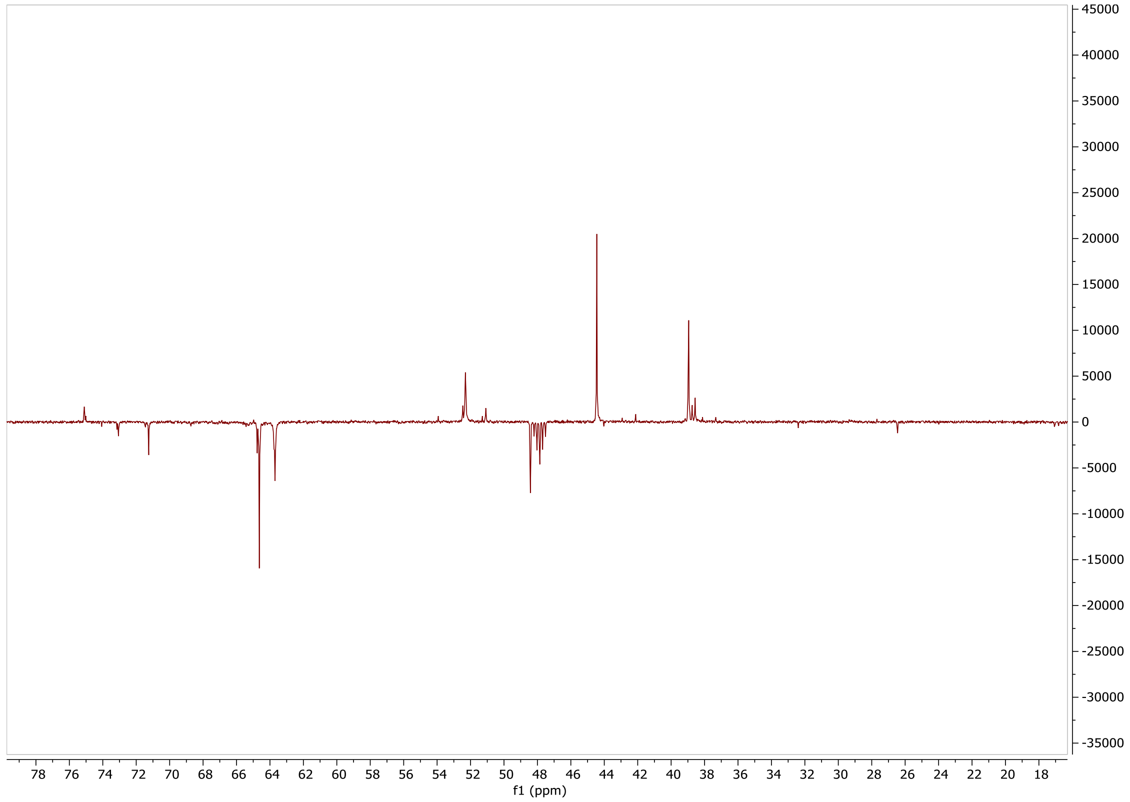

HSQC Spectrum of compound **28**

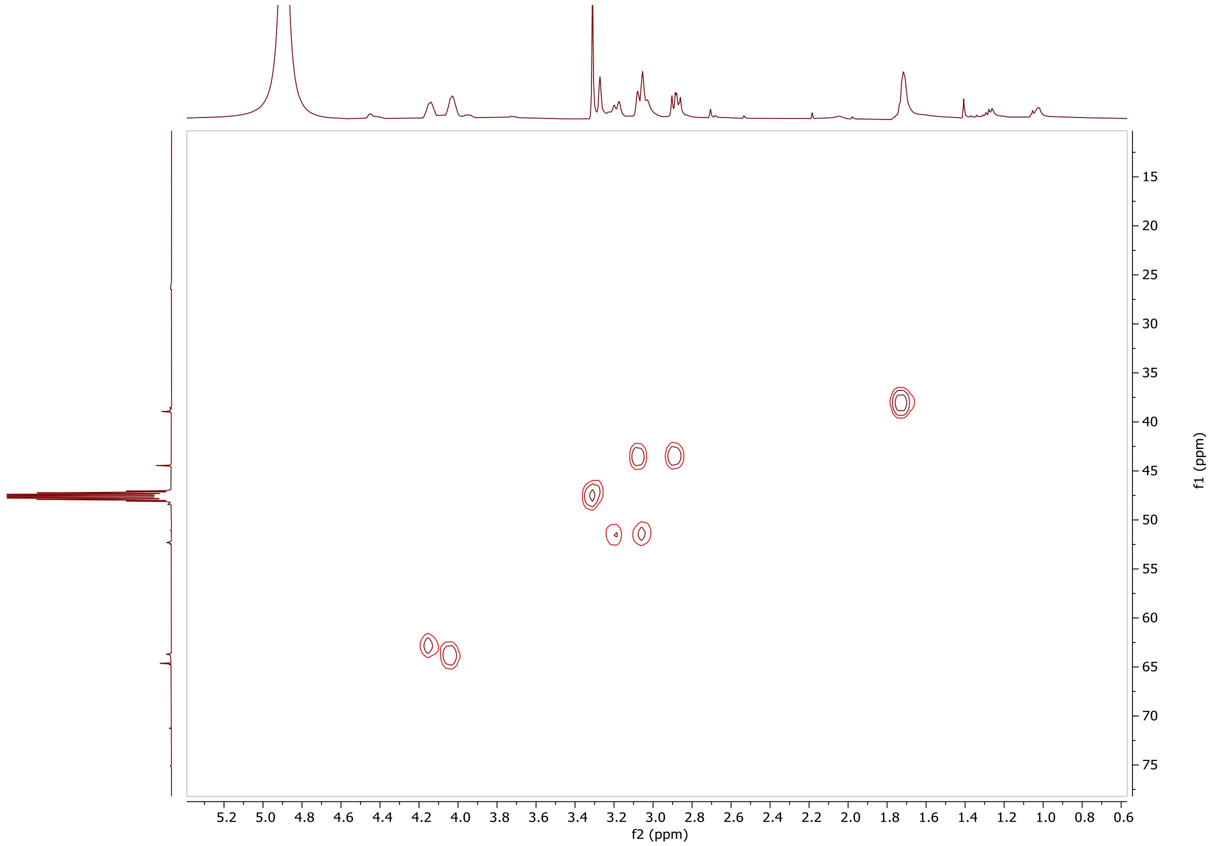

Supplement: File 1 — NMR data of all compounds. [file Beilstein_J_Org_Chem-17-1440-s001.pdf]
